# Data on molecular docking simulations of quaternary complexes 'Bst exo- polymerase-DNA-dCTP-metal cations'

**DOI:** 10.1016/j.dib.2020.106549

**Published:** 2020-11-19

**Authors:** Ravil R. Garafutdinov, Olga Yu. Kupova, Aidar R. Gilvanov, Assol R. Sakhabutdinova

**Affiliations:** a Institute of Biochemistry and Genetics, Ufa Federal Research Center of the Russian Academy of Sciences, Ufa, Russia; b Ufa State Petroleum Technological University, Ufa, Russia

**Keywords:** Bst exo- DNA polymerase, Cofactors, Molecular docking simulations, Ligand interaction diagrams, Polymerase active site

## Abstract

This article reports data related to the research article entitled “Effect of metal ions on isothermal amplification with Bst exo- DNA polymerase” (R.R. Garafutdinov, A.R. Gilvanov, O.Y. Kupova, A.R. Sakhabutdinova, 2020) [1]. Here, the results of molecular simulations of the complexes of Bst exo- DNA polymerase with dCTP triphosphate, double-stranded DNA and divalent metal cations are presented. Energetic parameters, number and type of chemical bonds formed by dCTP with the environment are given.

## Specifications Table

**Table:** 

Subject	Biochemistry, Molecular Biology, Molecular docking
Specific subject area	Protein structure, enzyme-substrate interaction
Type of data	Tables, Figures
How data were acquired	Molecular docking simulations of the quaternary complexes, consisted of triphosphate, DNA polymerase, DNA and metal cations were performed
Data format	Raw and analyzed
Parameters for data collection	The closed form of Bst exo- DNA polymerase (large fragment of DNA polymerase I from *Geobacillus stearothermophilus*) crystallized with short DNA duplex and triphosphate (dCTP) was taken (PDB: 1LV5). Modeling was performed with the cations Ca^2+^, Cd^2+^, Co^2+^, Cu^2+^, Mg^2+^, Mn^2+^, Ni^2+^, and Zn^2+^, which were placed into the positions A and B in different combinations of the initial structure followed by further optimization.
Description of data collection	Energetic parameters, number and type of chemical bonds formed by dCTP triphosphate with Bst exo- polymerase, DNA, cations and water molecules were found from ligand interaction diagrams.
Data source location	Institute of Biochemistry and Genetics Ufa Federal Research Center Russian Academy of Sciences, Ufa, Russia
Data accessibility	Raw data are provided in Supplementary file. All other data is with this article.
Related research article	R.R. Garafutdinov, A.R. Gilvanov, O.Y. Kupova, A.R. Sakhabutdinova Effect of metal ions on isothermal amplification with Bst exo- DNA polymerase Int. J. Biol. Macromol. 161 (2020) 1447-1455. DOI: 10.1016/j.ijbiomac.2020.08.028.

## Value of the Data

• The current data provide an important reference to enhance our understanding of the molecular mechanisms of DNA polymerases functioning.
• The obtained results can be claimed by researchers when studying the activity of polymerases or other enzymes using molecular dynamics methods.
• The results allow to roughly estimate the stability of the complexes and conclude about the ability of certain metal cations or cation pairs to be an effective cofactor of Bst exo- DNA polymerase.

## 1 Data Description

Bst exo- DNA polymerase (large fragment of DNA polymerase I from *Geobacillus stearothermophilus*) is an enzyme with strong strand-displacement activity, moderate thermal stability and high processivity, which is the most commonly used for isothermal amplification of nucleic acids. Unfortunately, this polymerase frequently results in non-specific amplification that leads to multimeric product formation [2,3]. Although the hypothesis on DNA multimerization [4] and its prevention strategies have been proposed [5,6], the search for methods that provide high specific amplification with Bst exo- polymerase is still relevant. The substitution of common cofactor Mg^2+^ for Mn^2+^ or other metal ions can modulate the activity of DNA polymerases [7]. It was shown that full Bst polymerase can utilize Mn^2+^, Co^2+^, and Cd^2+^ cations as alternative cofactors [8]. In this regard, the study of the structure of the complexes formed by polymerase with substrates and metal cofactors is of great interest.

Here, we present data on the optimized structure of the quaternary complexes containing a large fragment of Bst DNA polymerase, short DNA duplex, 2′-deoxycytidine-5′-triphosphate (dCTP), and cations Ca^2+^, Cd^2+^, Co^2+^, Cu^2+^, Mg^2+^, Mn^2+^, Ni^2+^, Zn^2+^. Molecular docking studies were performed using Schrödinger Suite 2016-4 software.

Calculations performed after optimization allowed to obtain three energetic parameters of the complexes: potential energy, docking score and glide emodel. Potential energy shows overall energy of the complexes, and the latter two values characterize the binding energy of triphosphate with the protein.

Ligand interaction diagrams (LIDs) for the complexes with all combinations of the cations placed on the positions A and B were obtained. Amino acids are represented at the LIDs as spheres colored according to their chemical properties. Chemical interactions between amino acids and ligands are shown as colored lines and arrows, depending on the type of interaction. LIDs do not show distances between ligand and receptor and are used only to display the number and type of bonds.

**Fig. 1:**
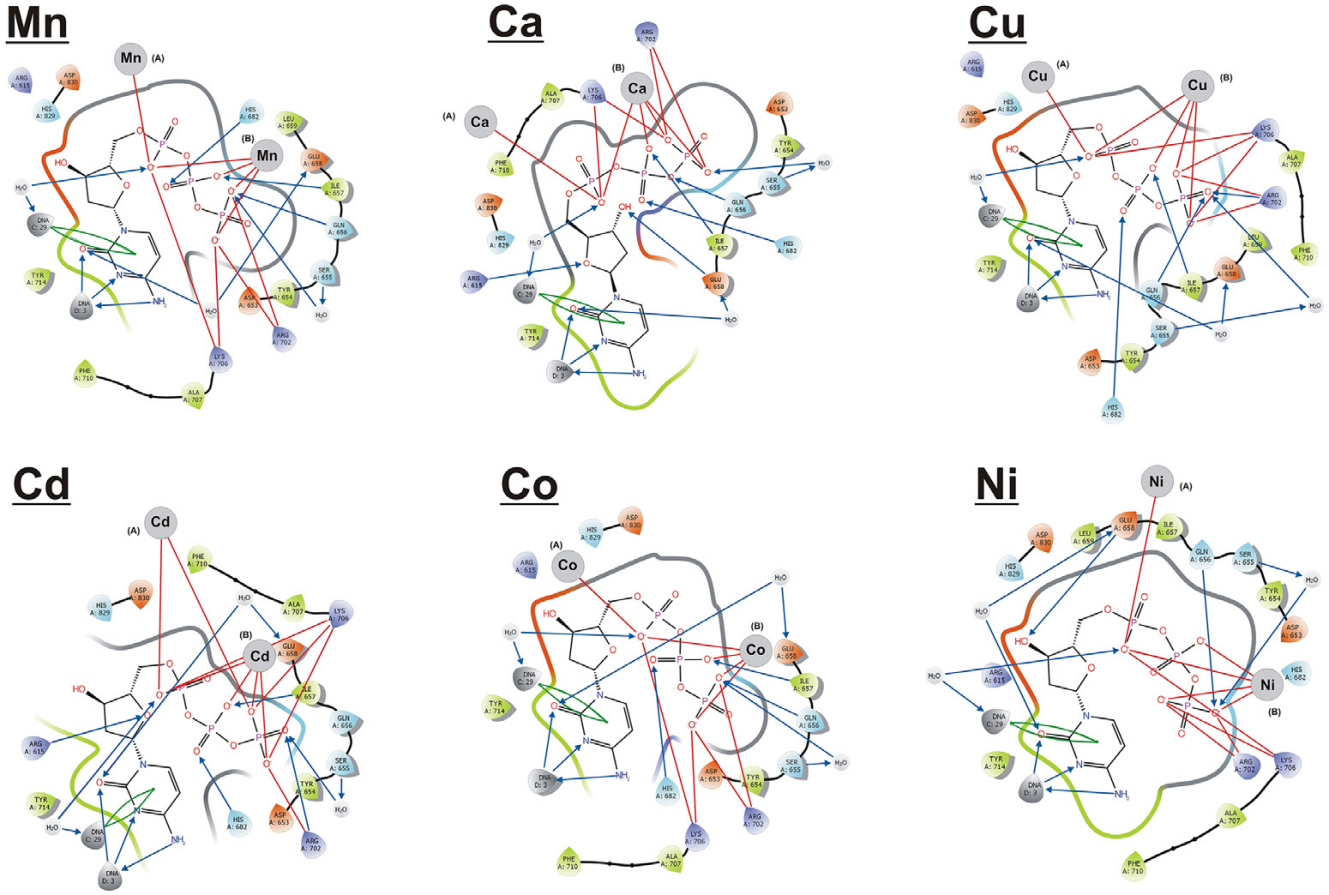
Ligand interaction diagrams (LIDs) for enzyme-substrate complexes with different metal ions: red lines - salt bridges, blue arrows - hydrogen bonds, green arrows - π-π interactions (LIDs for complexes with Mn^2+^ and Zn^2+^ have been previously published [1]).

Triphosphate binds by ionic (salt bridges, sb) and hydrogen (H) bonds with the environment: amino acids, cations, and DNA. LIDs showed that triphosphate binds by hydrogen and ionic bonds with a few amino acids, as well as by hydrogen bonds and π-π interactions (stacking interaction) with DNA. Cations interact only with the oxygen atoms of α and γ phosphate residues of triphosphate forming ionic bonds. The active site also contains two or three water molecules that bind by hydrogen bonds with amino acids, triphosphate and DNA. LIDs for the complexes with the same cation in both positions are shown in Fig. 1, except for the complexes with Mn^2+^ and Zn^2+^, previously demonstrated in [1]. The LIDs for all possible complexes are provided in Supplementary file.

The number and the type of chemical bonds are given in Table 1. A simple calculation of the total number of chemical bonds in the complexes allowed to determine the relative binding values (R), which can roughly characterize the stability of the structures. Raw data are provided in Supplementary file.

**Table 1:** Energetic parameters, number and type of chemical bonds (*) formed by triphosphate (dCTP) with Bst LF polymerase, DNA, cations and water molecules (a part of the data has been previously published [1]).

					Binding of triphosphate with										Binding of H_2_O with			total number of		
Cation position		Energetic parameters			Amino acids							cations								

site A	site B	potential energy	docking score	glide emodel	Arg 615	Gln 656	Ile 657	Glu 658	His 682	Arg 702	Lys 706	A	B	DNA	TP	AA	DNA	H bonds	Salt bridges	relative binding value (R)
Ca	Ca	−2754,803	−13,404	−132,495	1 H	1 H	1 H	1 H	1 H	2 sb	2 sb	1 sb	4 sb	3 H + 2 π-π	3 H	2 H	1 H	14	9	23
Ca	Cd	−2760,229	−10,554	−172,222		1 H	1 H		1 H	2 sb	2 sb	1 sb	4 sb	3 H + 2 π-π	3 H	2 H	1 H	12	9	21
Ca	Co	−2760,415	−13,653	−153,500		1 H	1 H		1 H	1 H + 2 sb	3 sb	1 sb	4 sb	3 H + 2 π-π	3 H	2 H	1 H	13	10	23
Ca	Cu	−2760,379	−11,873	−169,529		1 H	1 H	1 H	1 H	2 sb	3 sb	1 sb	4 sb	3 H + 1 π-π	3 H	2 H	1 H	13	10	23
Ca	Mg	−2761,017	−10,468	−191,692		1 H	1 H		1 H	2 sb	1 H + 3 sb	1 sb	4 sb	3 H + 2 π-π	3 H	2 H	1 H	13	10	23
Ca	Mn	−2760,435	−10,796	−175,556		1 H	1 H		1 H	2 sb	1 H + 3 sb	1 sb	4 sb	3 H + 2 π-π	3 H	2 H	1 H	13	10	23
Ca	Ni	−2760,414	−11,315	−244,798		1 H				2 H + 2 sb	4 sb	2 sb	3 sb	3 H + 2 π-π	2 H	1 H	1 H	10	11	21
Ca	Zn	−2760,854	−10,195	−256,765		1 H	1 H	1 H	1 H	2 sb	3 sb	1 sb	4 sb	3 H + 2 π-π	3 H	2 H	1 H	13	10	23
Cd	Ca	−2761,327	−13,283	−176,465		1 H	1 H	1 H	1 H	2 sb	3 sb	1 sb	4 sb	3 H + 2 π-π	3 H	2 H	1 H	13	10	23
Cd	Cd	−2751,903	−14,699	−167,137	1 H		1 H		1 H	1 H +1 sb	3 sb	2 sb	4 sb	3 H + 2 π-π	3 H	2 H	1 H	13	10	23
Cd	Co	−2758,729	−15,425	−177,182		1 H	1 H	1 H	1 H	1 H +1 sb	3 sb	2 sb	4 sb	3 H + 2 π-π	2 H	2 H		12	10	22
Cd	Cu	−2758,933	−15,020	−169,641		1 H	1 H		1 H	1 H +1 sb	3 sb	1 sb	4 sb	3 H + 2 π-π	3 H	2 H	1 H	13	9	22
Cd	Mg	−2760,138	−14,616	−190,113		1 H	1 H	1 H	1 H	2 sb	2 sb	1 sb	4 sb	3 H + 2 π-π	3 H	2 H	1 H	13	9	22
Cd	Mn	−2759,017	−13,695	−164,174		1 H		1 H		2 sb	2 sb	1 sb	4 sb	3 H + 2 π-π	3 H	2 H	1 H	11	9	20
Cd	Ni	−2759,002	−11,115	−261,086		1 H		1 H		2 H + 1 sb	3 sb	1 sb	2 sb	3 H + 2 π-π	2 H	1 H	1 H	11	7	18
Cd	Zn	−2759,817	−12,734	−299,601	1 H			1 H		1 H +1 sb	1 H + 3 sb	1 sb	4 sb	3 H + 2 π-π	3 H	2 H	1 H	13	9	22
Co	Ca	−2761,402	−15,045	−172,764		1 H	1 H	1 H	1 H	2 sb	2 sb	1 sb	4 sb	3 H + 2 π-π	2 H	2 H		11	9	20
Co	Cd	−2759,043	−15,086	−170,407		1 H	1 H		1 H	1 H + 1 sb	1 H + 3 sb	2 sb	4 sb	3 H + 2 π-π	3 H	2 H	1 H	14	10	24
Co	Co	−2759,089	−15,742	−171,832		1 H	1 H		1 H	2 sb	2 sb	1 sb	4 sb	3 H + 2 π-π	3 H	2 H	1 H	12	9	21
Co	Cu	−2759,175	−14,563	−165,880		1 H	1 H		1 H	1 H +1 sb	1 H + 2 sb	1 sb	4 sb	3 H + 2 π-π	3 H	2 H	1 H	14	8	22
Co	Mg	−2760,150	−14,940	−179,882	1 H	1 H	1 H		1 H	2 sb	2 sb	1 sb	4 sb	3 H + 2 π-π	3 H	2 H	1 H	13	9	22
Co	Mn	−2759,118	−13,830	−177,349		1 H	1 H		1 H	2 sb	2 sb	1 sb	4 sb	3 H + 2 π-π	3 H	2 H	1 H	12	9	21
Co	Ni	−2759,108	−12,829	−294,608	1 H					1 H +1 sb	1 H + 2 sb	1 sb	4 sb	3 H + 2 π-π	3 H	2 H	1 H	12	8	20
Co	Zn	−2759,908	−12,535	−324,081	1 H		1 H	1 H	1 H	1 H +1 sb	1 H + 3 sb	1 sb	4 sb	3 H + 2 π-π	3 H	2 H	1 H	15	9	24
Cu	Ca	−2742,302	−14,255	−196,373		1 H	1 H		1 H	1 H + 2 sb	1 H + 3 sb	1 sb	4 sb	3 H + 2 π-π	3 H	2 H	1 H	14	10	24
Cu	Cd	−2757,518	−15,442	−189,664		1 H	1 H		1 H	2 sb	3 sb	1 sb	4 sb	3 H + 2 π-π	3 H	2 H	1 H	12	10	22
Cu	Co	−2757,657	−15,470	−170,079		2 H		1 H	1 H	2 sb	2 sb	1 sb	4 sb	3 H + 2 π-π	3 H	2 H	1 H	13	9	22
Cu	Cu	−2757,612	−14,938	−198,117		1 H	1 H		1 H	1 H + 2 sb	3 sb	1 sb	4 sb	3 H + 2 π-π	3 H	2 H	1 H	13	10	23
Cu	Mg	−2758,378	−10,466	−254,488		1 H		1 H		2 H + 2 sb	4 sb	2 sb	3 sb	3 H + 2 π-π	2 H	1 H	1 H	11	11	22
Cu	Mn	−2757,645	−15,371	−173,488			1 H	1 H	1 H	2 sb	2 sb	1 sb	4 sb	3 H + 2 π-π	3 H	2 H	1 H	12	9	21
Cu	Ni	−2757,662	−11,496	−269,814	1 H	2 H			1 H	1 H +1 sb	3 sb	2 sb	4 sb	1 H + 1 π-π	2 H	1 H	1 H	10	10	20
Cu	Zn	−2758,193	−12,853	−298,672	1 H					2 sb	2 sb	1 sb	4 sb	3 H + 2 π-π	3 H	2 H	1 H	10	9	19
Mg	Ca	−2765,108	−13,039	−175,300				1 H	1 H	1 H +1 sb	3 sb	2 sb	4 sb	3 H + 2 π-π	3 H	2 H	1 H	12	10	22
Mg	Cd	−2763,203	−12,843	−201,032		1 H	1 H		1 H	1 H +1 sb	3 sb	2 sb	4 sb	3 H + 2 π-π	3 H	2 H	1 H	13	10	23
Mg	Co	−2763,333	−12,606	−197,915		1 H	1 H		1 H	1 H +1 sb	3 sb	2 sb	4 sb	3 H + 2 π-π	3 H	2 H	1 H	13	10	23
Mg	Cu	−2763,292	−12,386	−189,971		1 H	1 H		1 H	2 sb	2 sb	1 sb	4 sb	3 H + 2 π-π	3 H	2 H	1 H	12	9	21
Mg	Mg	−2763,952	−11,609	−203,648		1 H	1 H	1 H	1 H	1 H +1 sb	1 H + 2 sb	2 sb	4 sb	3 H + 2 π-π	3 H	2 H	1 H	15	9	24
Mg	Mn	−2763,350	−11,580	−191,424		1 H	1 H		1 H	1 H +1 sb	1 H + 3 sb	2 sb	4 sb	3 H + 2 π-π	3 H	2 H	1 H	14	10	24
Mg	Ni	−2763,334	−12,373	−284,370				1 H		2 H + 1 sb	3 sb	1 sb	4 sb	3 H + 2 π-π	3 H	2 H	1 H	12	9	21
Mg	Zn	−2763,843	−11,282	−274,003		1 H	1 H	1 H	1 H	2 sb	3 sb	1 sb	4 sb	1 H + 2 π-π	2 H	2 H	1 H	10	10	20
Mn	Ca	−2760,832	−15,234	−167,510		1 H	1 H	1 H	1 H	1 H + 1 sb	3 sb	2 sb	4 sb	3 H + 2 π-π	2 H	2 H		12	10	22
Mn	Cd	−2759,141	−14,995	−166,450						2 sb	2 sb	1 sb	4 sb	3 H + 2 π-π	3 H	2 H	1 H	9	9	18
Mn	Co	−2759,122	−14,279	−178,656		1 H	1 H	1 H	1 H	1 H +1 sb	1 H + 2 sb	1 sb	4 sb	3 H + 2 π-π	3 H	2 H	1 H	15	8	23
Mn	Cu	−2759,395	−14,307	−170,954		1 H	1 H		1 H	2 sb	3 sb	1 sb	4 sb	3 H + 2 π-π	3 H	2 H	1 H	12	10	22
Mn	Mg	−2759,589	−12,620	−305,576	1 H		1 H	1 H	1 H	1 H +1 sb	1 H + 2 sb	2 sb	4 sb	3 H + 2 π-π	3 H	2 H	1 H	15	9	24
Mn	Mn	−2751,969	−15,088	−194,809		1 H	1 H		1 H	2 sb	2 sb	1 sb	4 sb	3 H + 2 π-π	3 H	2 H	1 H	12	9	21
Mn	Ni	−2759,113	−13,466	−334,837		1 H	1 H		1 H	1 H +1 sb	3 sb	2 sb	4 sb	3 H + 2 π-π	3 H	2 H	1 H	13	10	23
Mn	Zn	−2759,452	−12,771	−298,184	1 H	1 H	1 H	1 H	1 H	1 H +1 sb	1 H + 2 sb	1 sb	4 sb	3 H + 2 π-π	3 H	2 H	1 H	16	8	24
Ni	Ca	−2744,497	−14,841	−183,120		1 H	1 H	1 H	1 H	1 H +1 sb	3 sb	1 sb	4 sb	3 H + 2 π-π	3 H	2 H	1 H	14	9	23
Ni	Cd	−2759,130	−13,827	−150,450	1 H					1 H	1 H + 3 sb	3 sb	4 sb	3 H + 2 π-π	2 H	1 H	1 H	10	10	20
Ni	Co	−2759,243	−15,352	−164,046	1 H	1 H	1 H		1 H	1 H +1 sb	3 sb	2 sb	4 sb	3 H + 2 π-π	3 H	2 H	1 H	14	10	24
Ni	Cu	−2759,212	−15,133	−176,972		1 H	1 H	1 H	1 H	1 H +1 sb	3 sb	2 sb	4 sb	3 H + 2 π-π	2 H	2 H		12	10	22
Ni	Mg	−2760,289	−11,068	−264,131		1 H		1 H		3 H	1 H + 3 sb	2 sb	3 sb	3 H + 2 π-π	2 H	1 H	1 H	13	8	21
Ni	Mn	−2759,257	−13,123	−162,354	1 H	1 H	1 H	1 H	1 H	1 H +1 sb	1 H + 3 sb	1 sb	4 sb	3 H + 2 π-π	3 H	2 H	1 H	16	9	25
Ni	Ni	−2759,252	−14,913	−176,985		1 H		1 H		2 sb	2 sb	1 sb	4 sb	3 H + 2 π-π	3 H	2 H	1 H	11	9	20
Ni	Zn	−2760,034	−11,135	−268,373		1 H		1 H		2 H + 1 sb	3 sb	2 sb	3 sb	3 H + 2 π-π	2 H	1 H	1 H	11	9	20
Zn	Ca	−2763,363	−12,208	−179,541		1 H	1 H		1 H	2 sb	2 sb	1 sb	4 sb	3 H + 2 π-π	3 H	2 H	1 H	12	9	21
Zn	Cd	−2761,390	−12,431	−174,903						2 sb	2 sb	1 sb	4 sb	3 H + 2 π-π	3 H	2 H	1 H	9	9	18
Zn	Co	−2761,524	−12,809	−199,065		1 H	1 H		1 H	2 sb	2 sb	1 sb	4 sb	3 H + 2 π-π	3 H	2 H	1 H	12	9	21
Zn	Cu	−2761,459	−12,261	−184,822		1 H	1 H		1 H	1 H +1 sb	3 sb	2 sb	4 sb	3 H + 2 π-π	3 H	2 H	1 H	13	10	23
Zn	Mg	−2762,151	−12,051	−293,844		1 H				2 H + 1 sb	3 sb	2 sb	3 sb	3 H + 2 π-π	2 H	1 H	1 H	10	9	19
Zn	Mn	−2761,538	−13,391	−181,460	1 H	1 H	1 H		1 H	2 sb	2 sb	1 sb	4 sb	3 H + 2 π-π	3 H	2 H	1 H	13	9	22
Zn	Ni	−2761,527	−11,276	−278,460		1 H		1 H		2 H + 1 sb	3 sb	1 sb	2 sb	3 H + 2 π-π	2 H	1 H	1 H	11	7	18
Zn	Zn	−2762,022	−12,576	−177,136	1 H					1 H + 1 sb	3 sb	1 sb	4 sb	3 H + 2 π-π	3 H	2 H	1 H	11	9	20

⁎ H – hydrogen bonds, sb - salt bridges, π-π - π-π-interactions.

## 2 Experimental Design, Materials and Methods

### 2.1 Molecular docking simulations

Molecular docking simulations were performed using Schrodinger Suite 2016-4 [9]. 2′-deoxycytidine-5′-triphosphate (dCTP) molecule was set as a ligand, and the closed form of Bst exo- DNA polymerase bound to DNA was set as a protein.

The structural coordinates of Bst exo- DNA polymerase complexed with DNA and the incoming dCTP was taken from 1LV5 crystal structure (1.95 Å resolution) [10]. Polymerase structure was prepared for docking simulations by adding missing hydrogen atoms, assigning correct bond orders and building disulfide bonds in Protein Preparation Wizard module [11]. dCTP was taken from the original crystal structure and left unchanged.

Cations Ca^2+^, Cd^2+^, Co^2+^, Cu^2+^, Mg^2+^, Mn^2+^, Ni^2+^ and Zn^2+^ were placed into the positions A and B of the initial structure, and further optimization of the structure (restrained minimization for all atoms that allows hydrogen atoms to be freely minimized while allowing for sufficient heavy-atom movement to relax strained bonds, angles, and clashes to remove steric clashes within the molecule) was carried out in the Protein Preparation Wizard module. After that metal ionization states were corrected to ensure proper formal charge and force field treatment. The protonation states for all ionizable residues in the presence of the metal ions were predicted by PROPKA provided in the Protein Preparation Wizard. An optimized structure model was energy minimized (only hydrogen atoms with converge heavy atoms to RMSD below 0.3 Å) using the OPLS2005 force field. The orientation of hydrogen atoms in the water molecules was sampled using PROPKA as well.

The receptor grid generation module of Glide [12], [13], [14], [15] was used to define the polymerase active site for docking experiments. As the protein has a bound ligand (dCTP), the ligand was set as the centroid of the grid box (the size of the active site is 40 Å from ligand position). Extra precision (XP) docking modes were used and the top 10 binding poses were analyzed after the post-minimization process (the threshold for rejecting the minimized pose was set to 0.5 kcal/mol).

## Declaration of Competing Interest

The authors declare that they have no known competing financial interests or personal relationships which have, or could be perceived to have, influenced the work reported in this article.

## Appendix Supplementary materials

Image, application 1
