# Supplementary figures and images for: Data on molecular docking simulations of quaternary complexes 'Bst exo- polymerase-DNA-dCTP-metal cations'
Source: Data Brief. 2020 Nov 19;33:106549. doi: 10.1016/j.dib.2020.106549 (PMC7704292; doi:10.1016/j.dib.2020.106549)

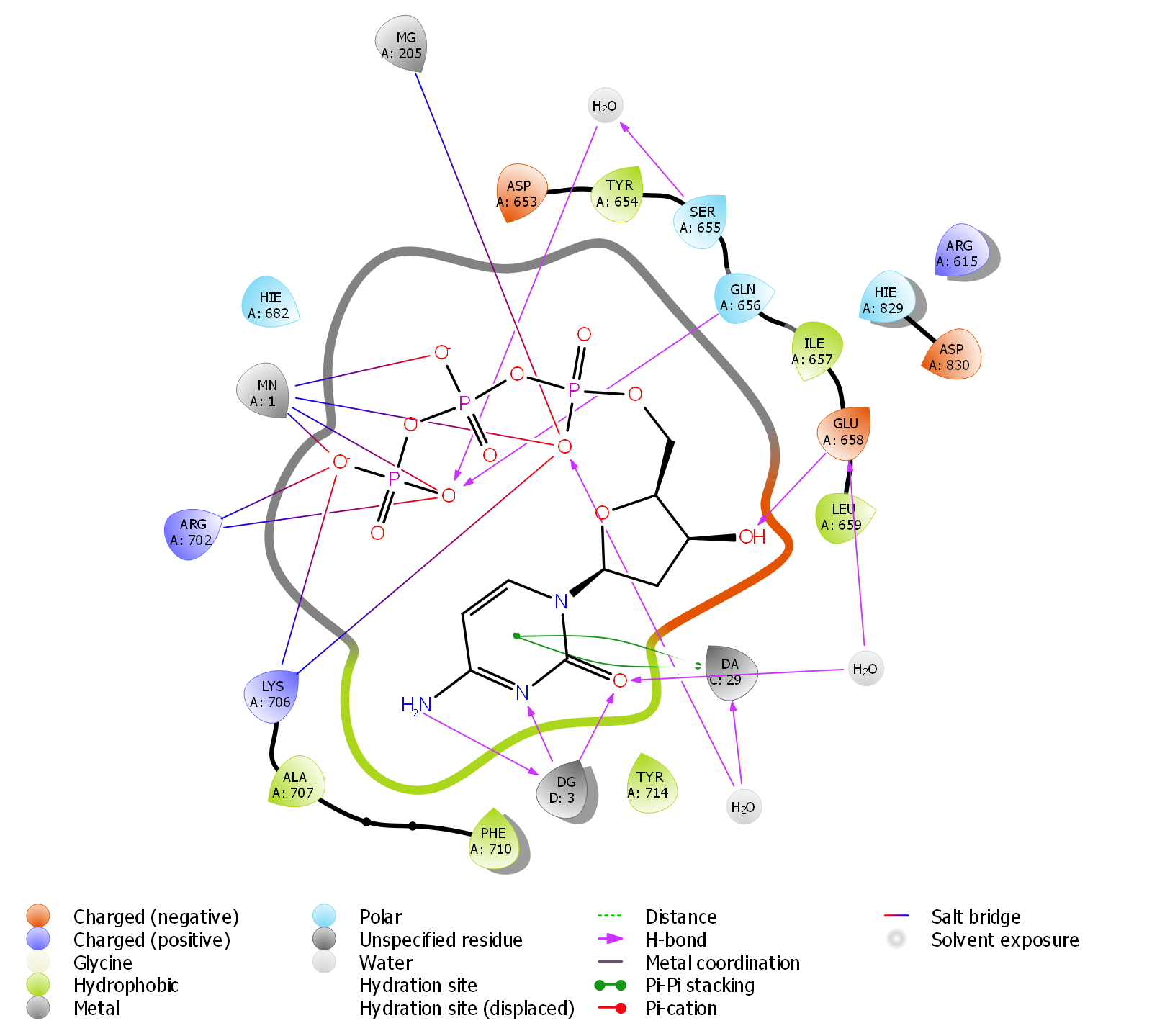

Supplement: Supplementary file 1 [file mmc1.zip › Ni_Ni.png]

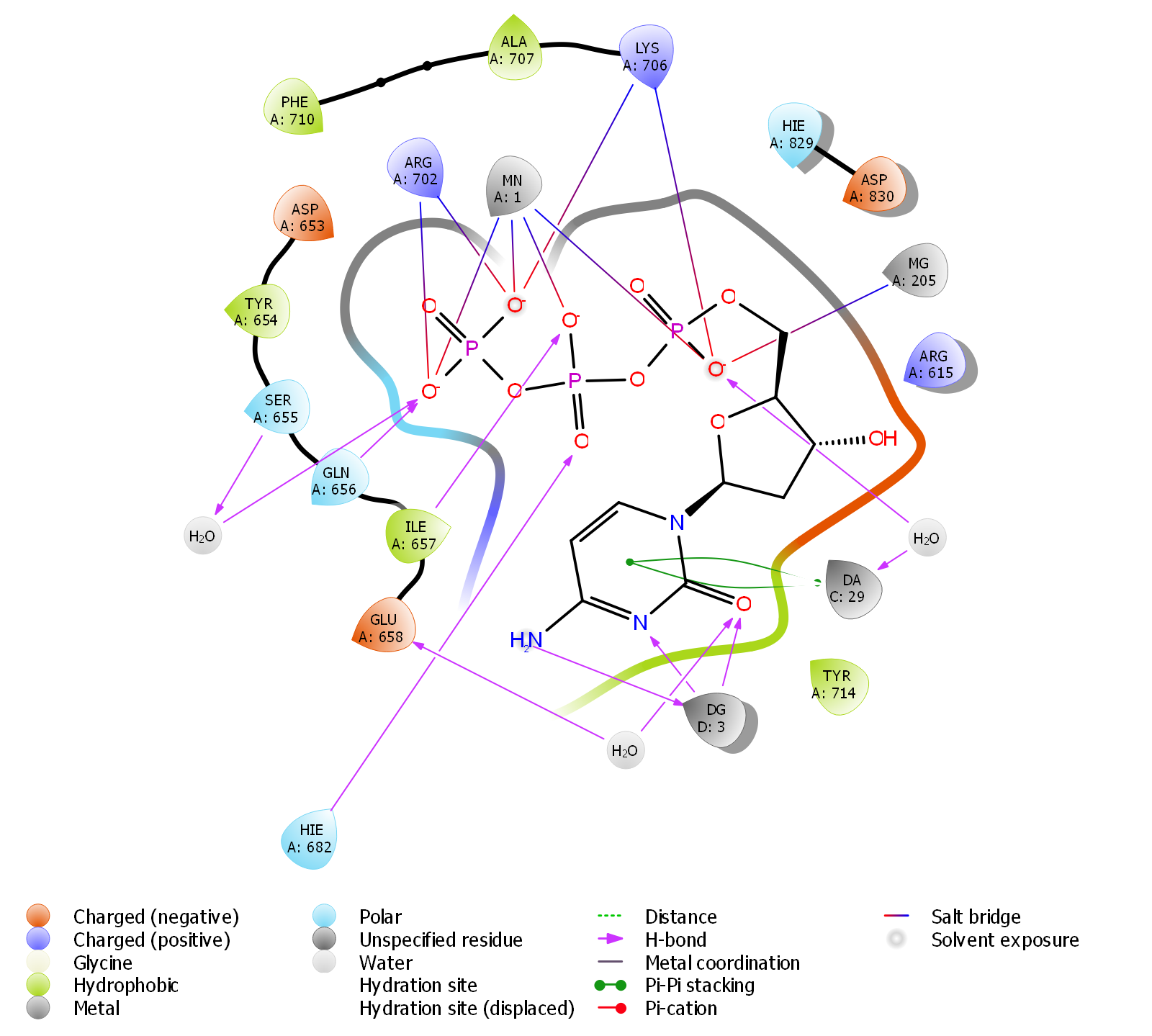

Supplement: Supplementary file 1 [file mmc1.zip › Co_Co.png]

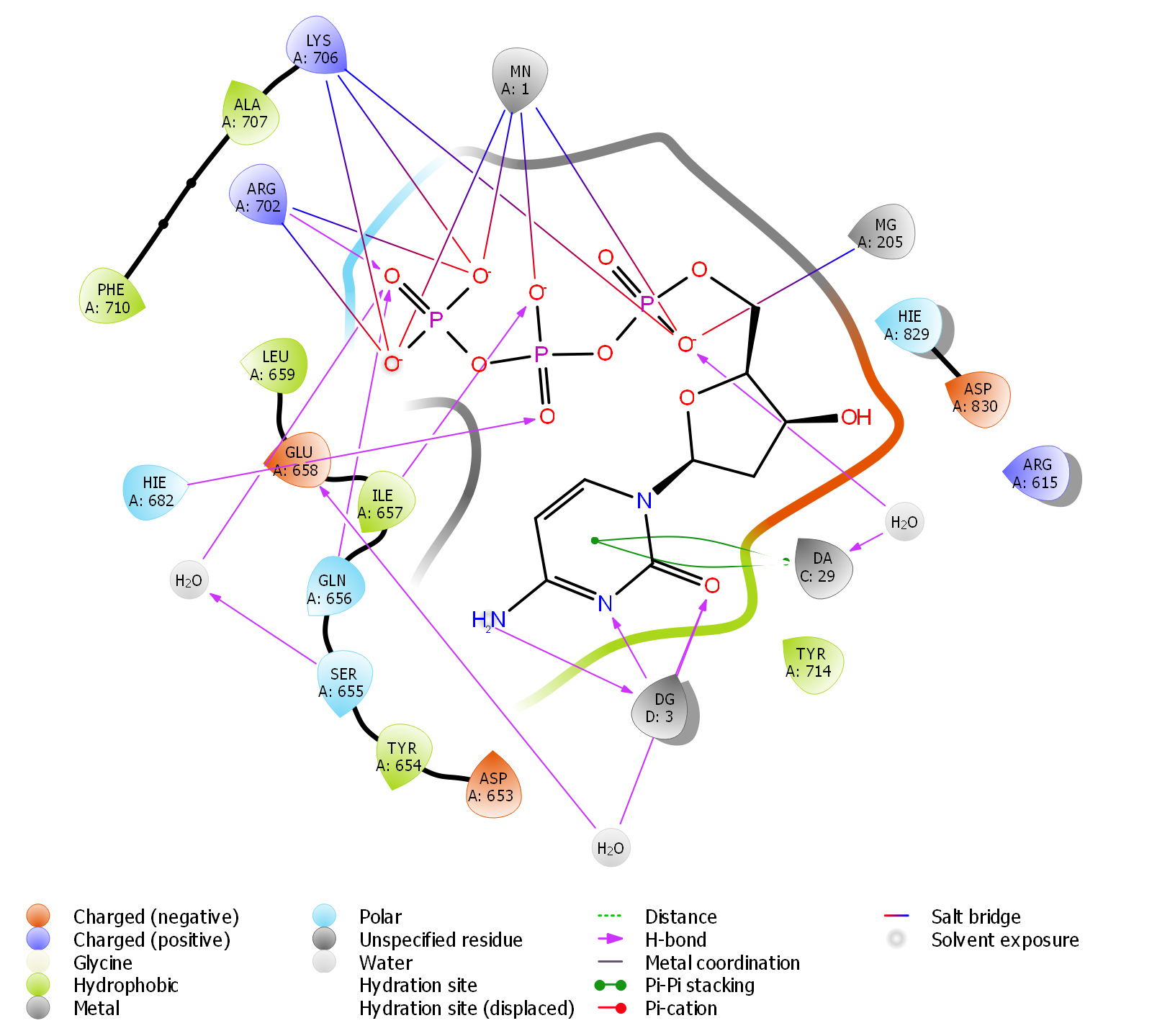

Supplement: Supplementary file 1 [file mmc1.zip › Cu_Cu.png]

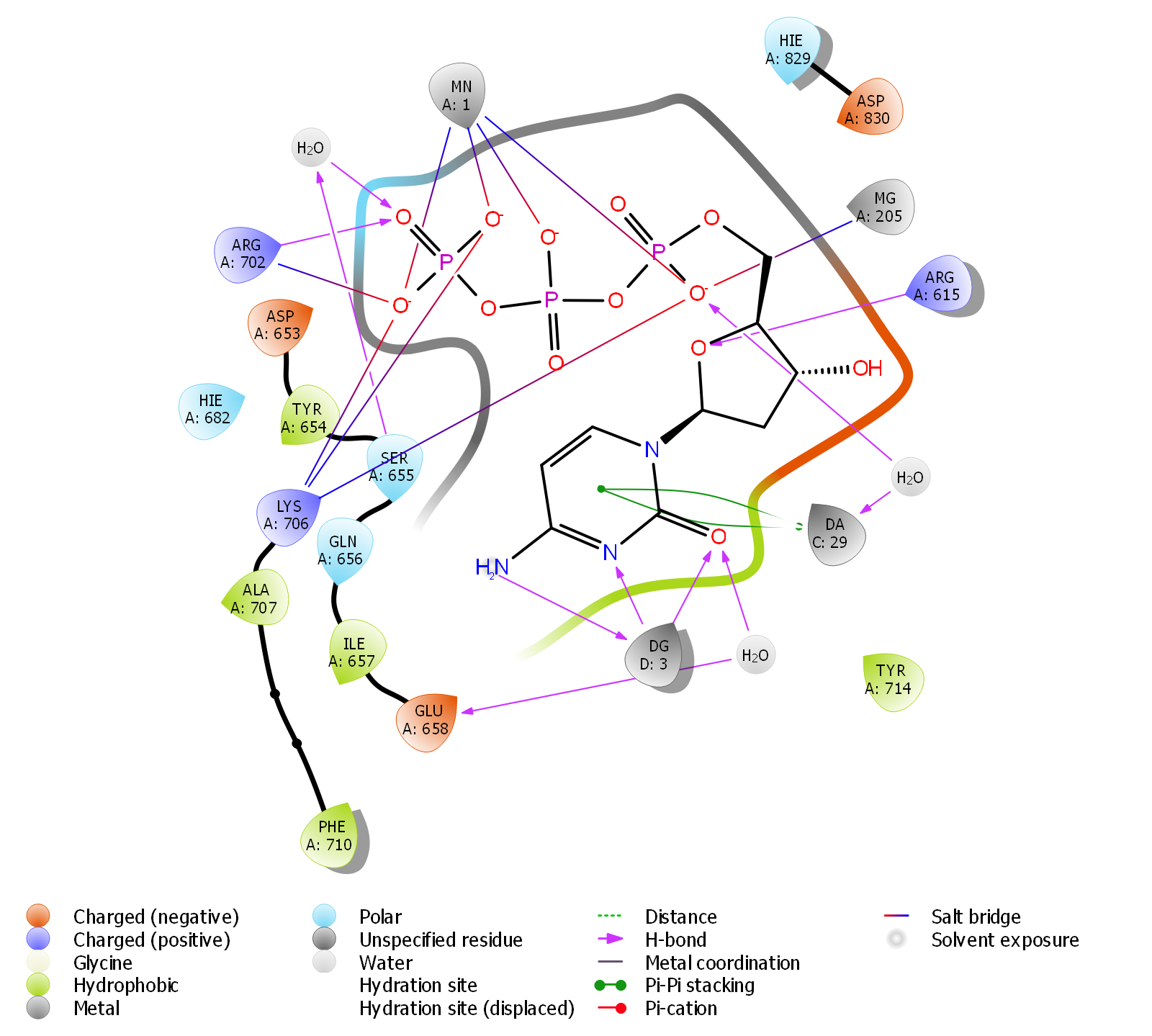

Supplement: Supplementary file 1 [file mmc1.zip › Zn_Zn.png]

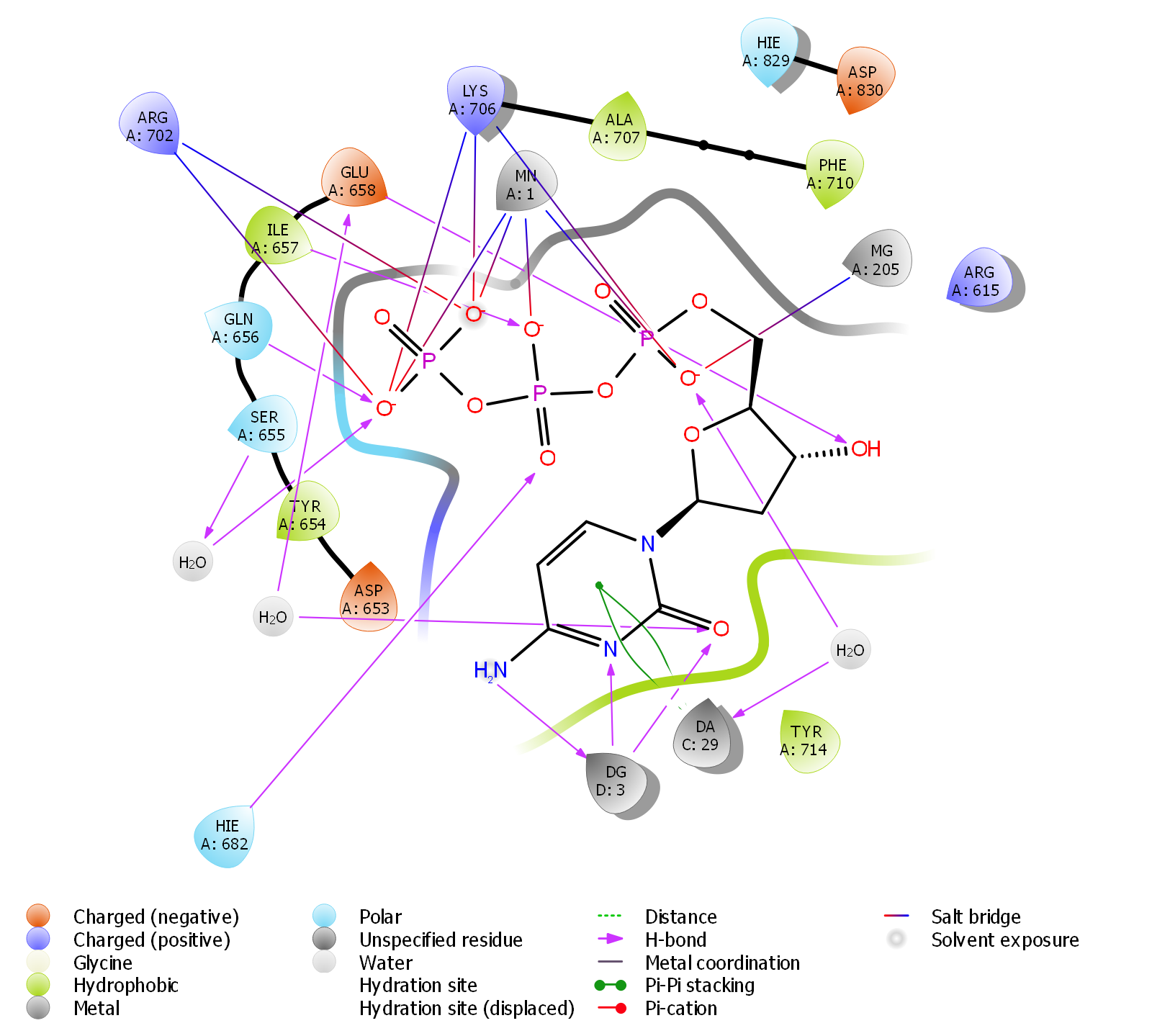

Supplement: Supplementary file 1 [file mmc1.zip › Cd_Ca.png]

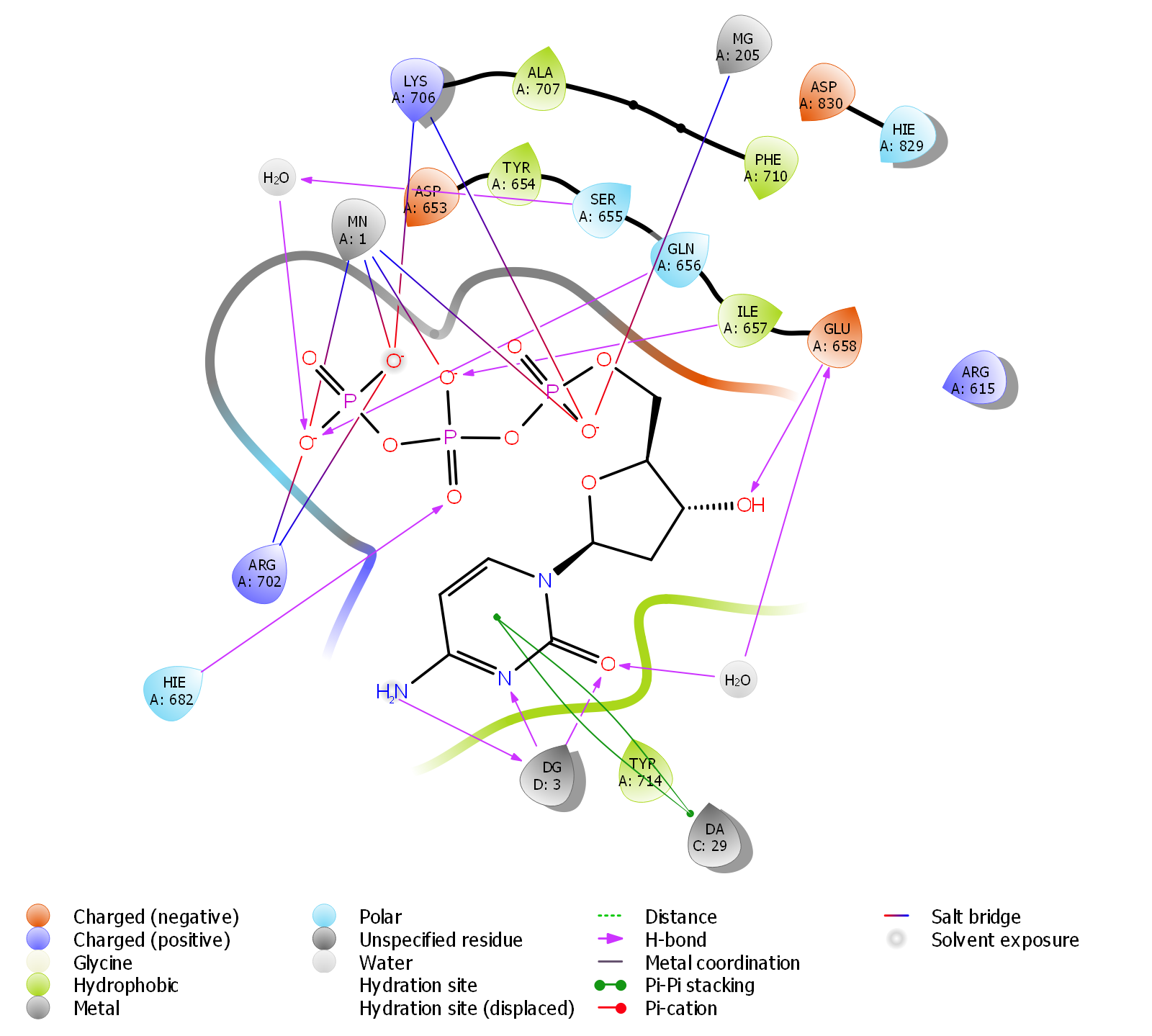

Supplement: Supplementary file 1 [file mmc1.zip › Co_Ca.png]

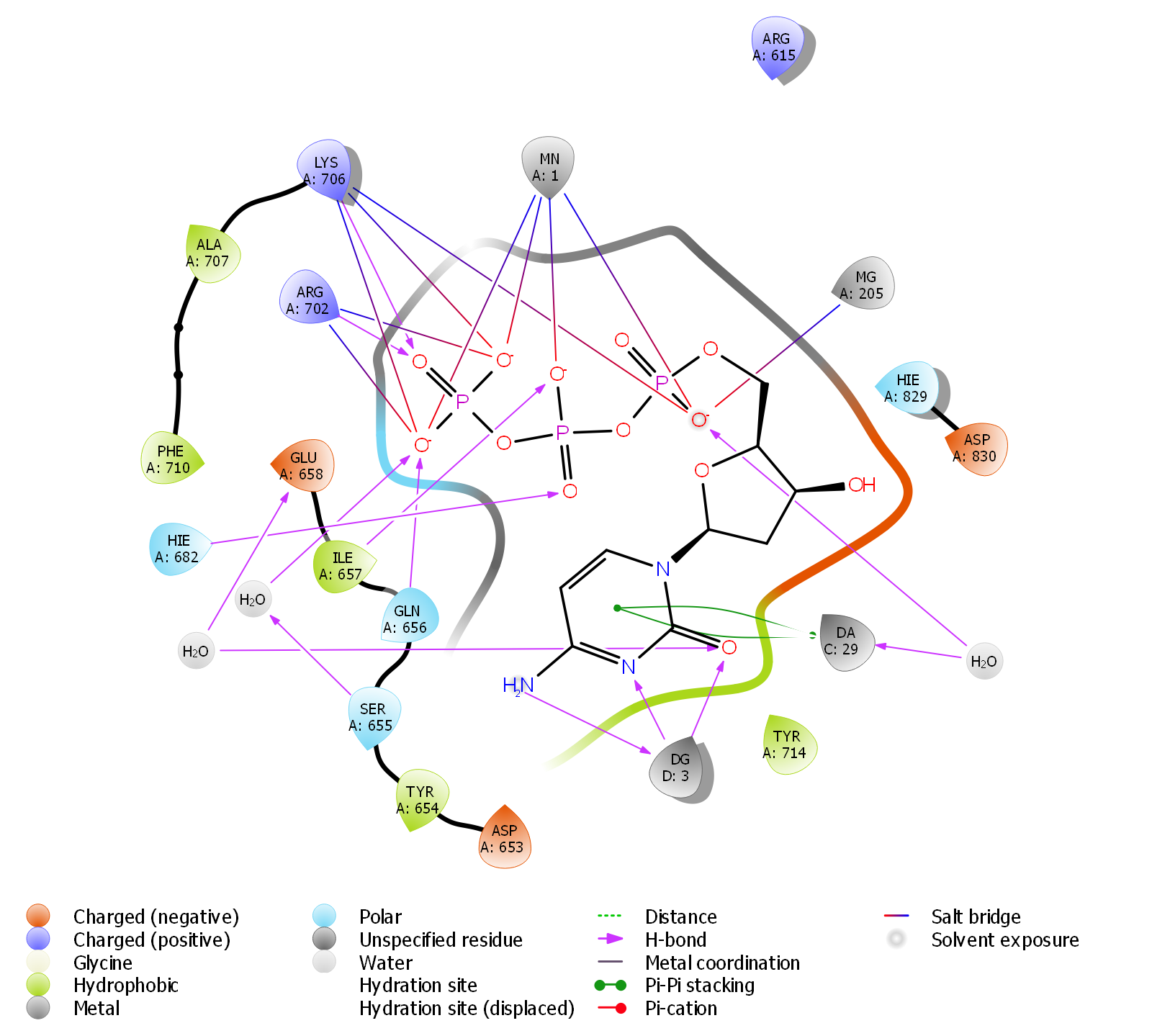

Supplement: Supplementary file 1 [file mmc1.zip › Cu_Ca.png]

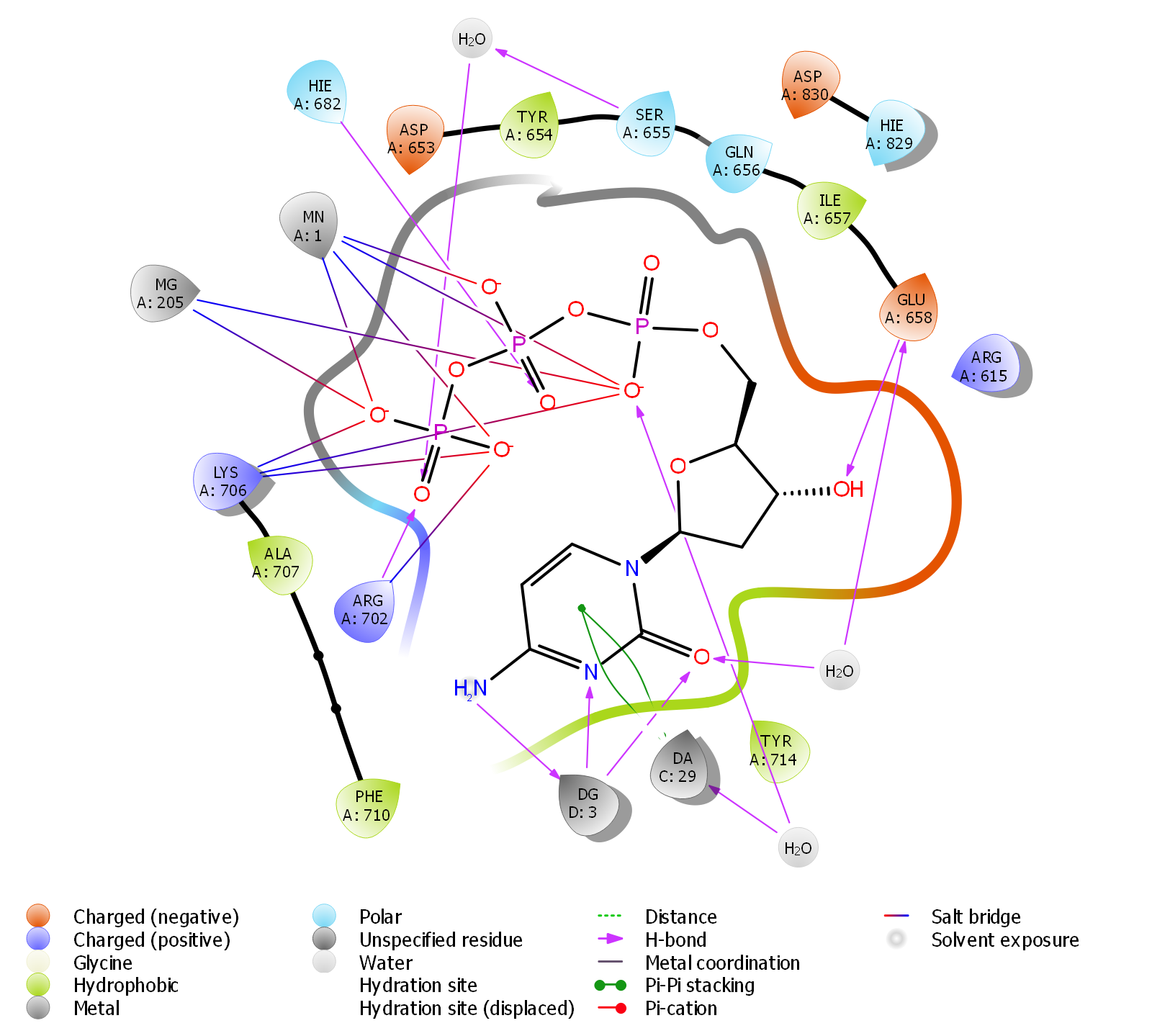

Supplement: Supplementary file 1 [file mmc1.zip › Mg_Ca.png]

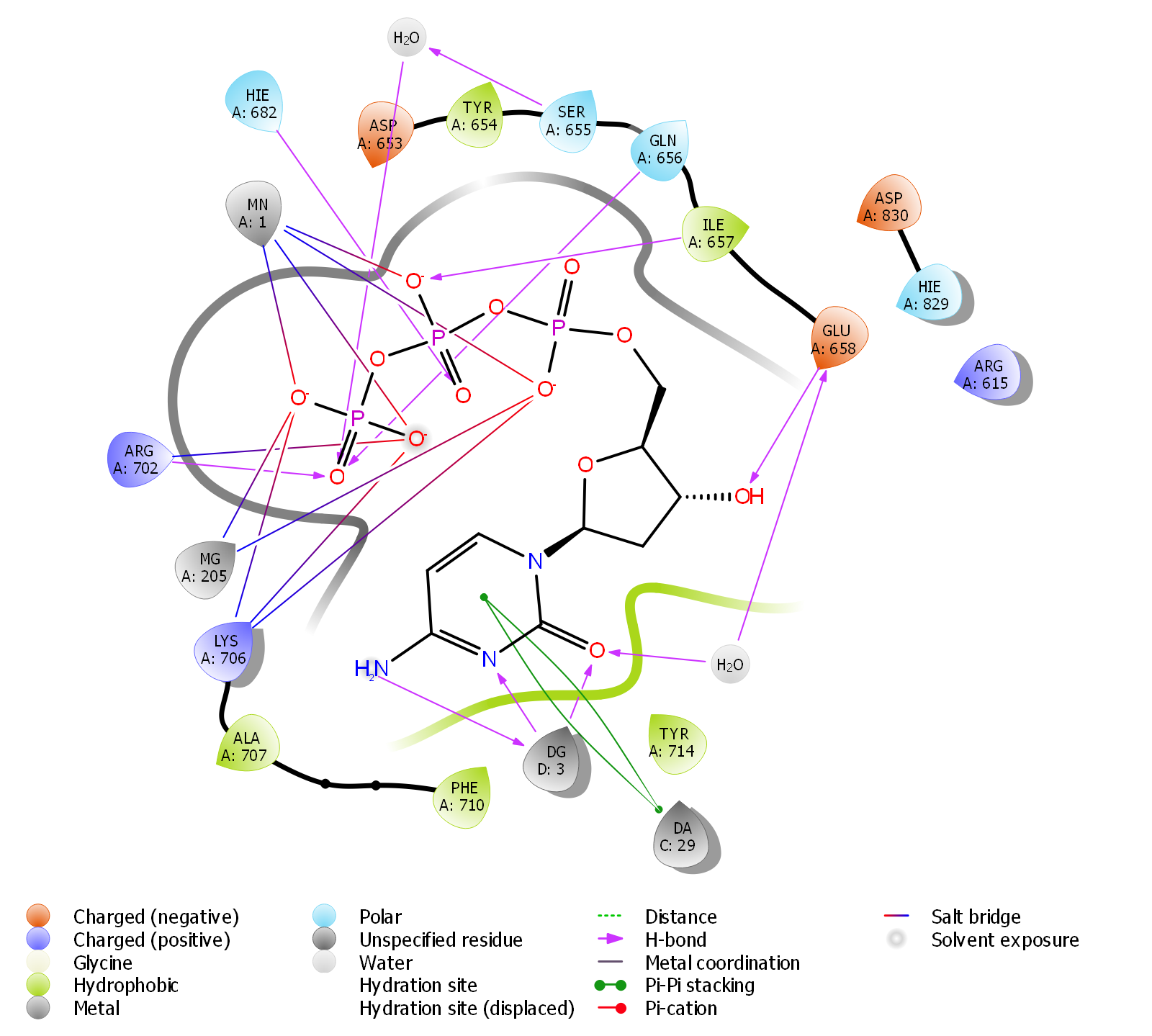

Supplement: Supplementary file 1 [file mmc1.zip › Mn_Ca.png]

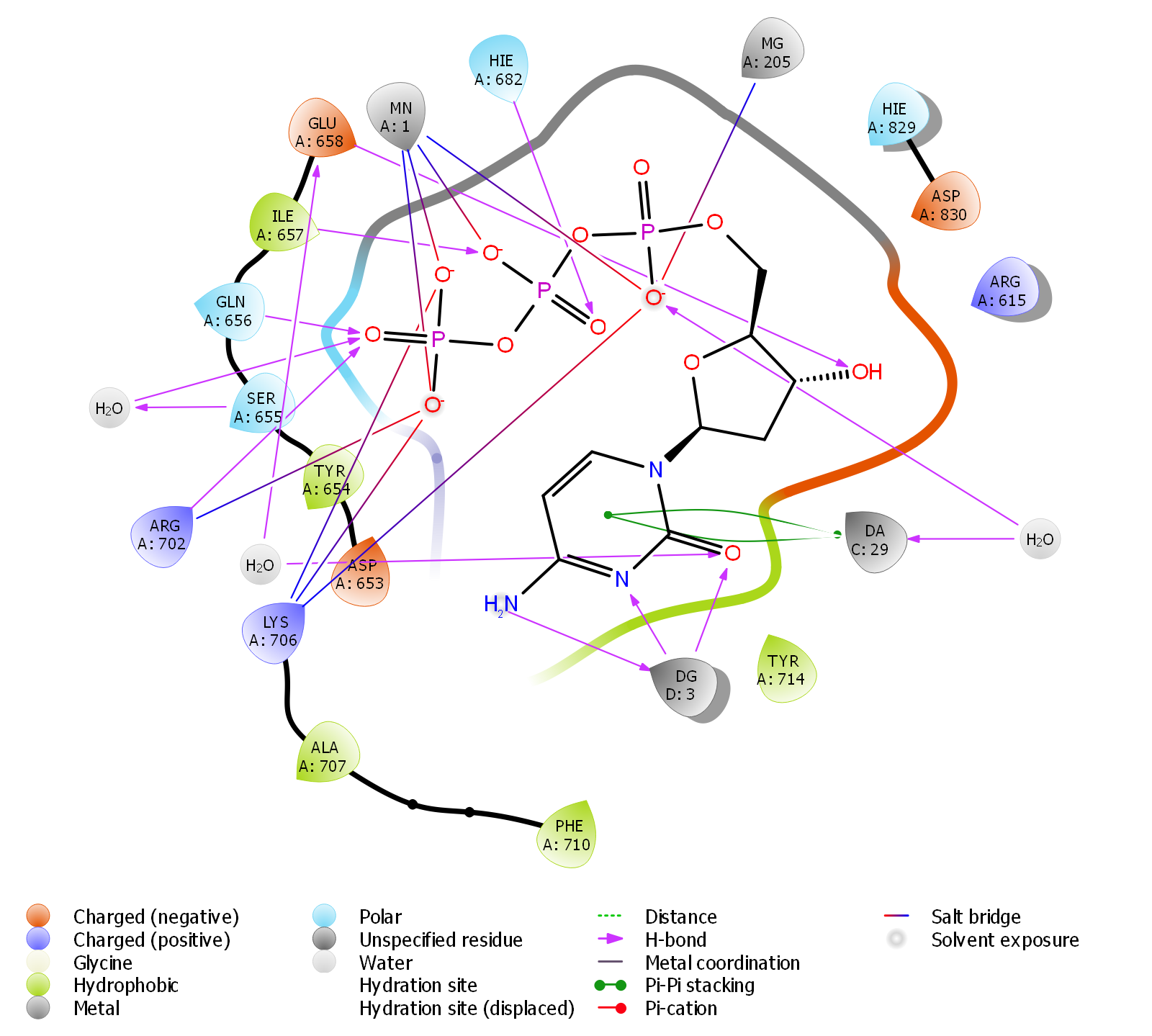

Supplement: Supplementary file 1 [file mmc1.zip › Ni_Ca.png]

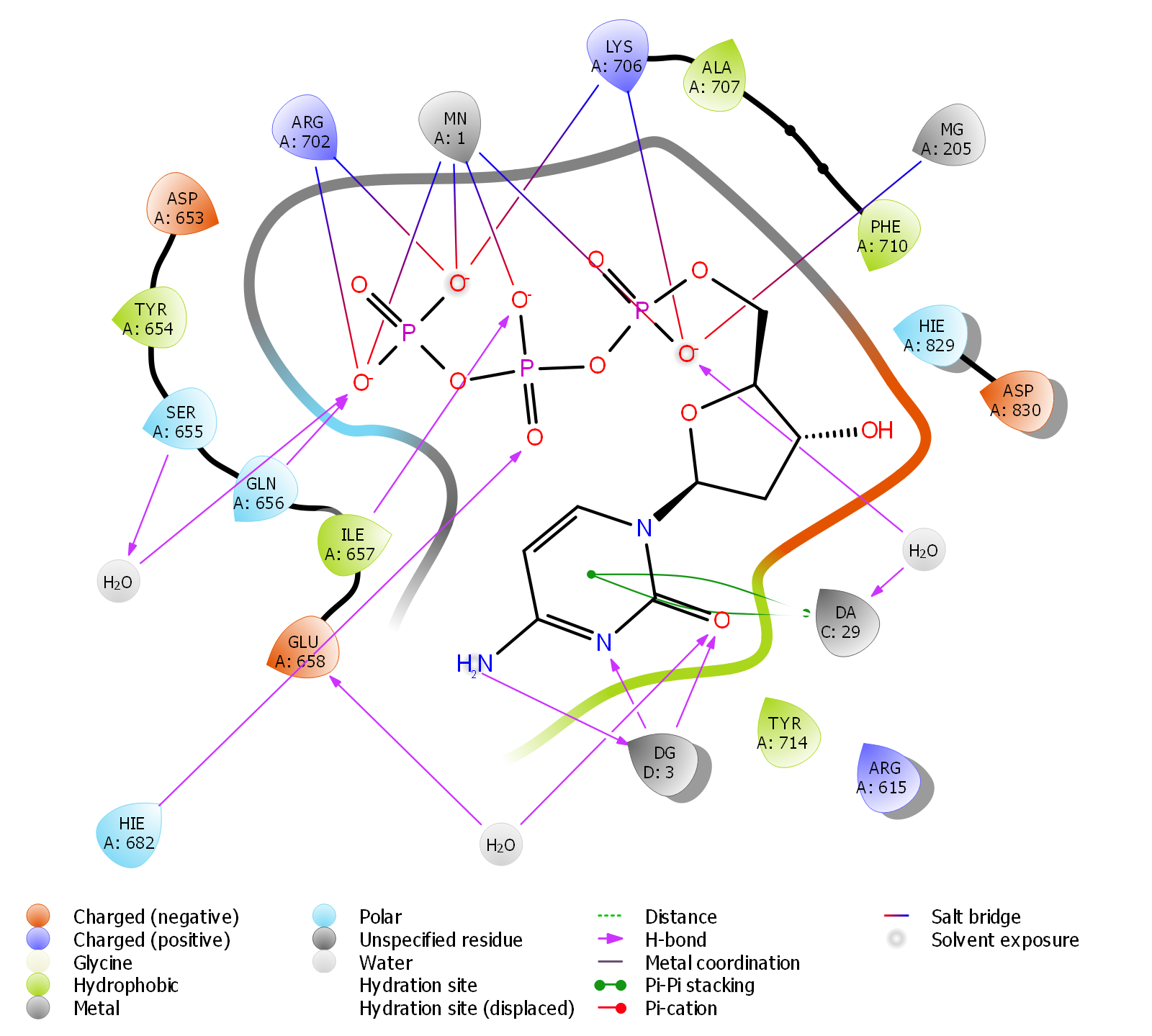

Supplement: Supplementary file 1 [file mmc1.zip › Zn_Ca.png]

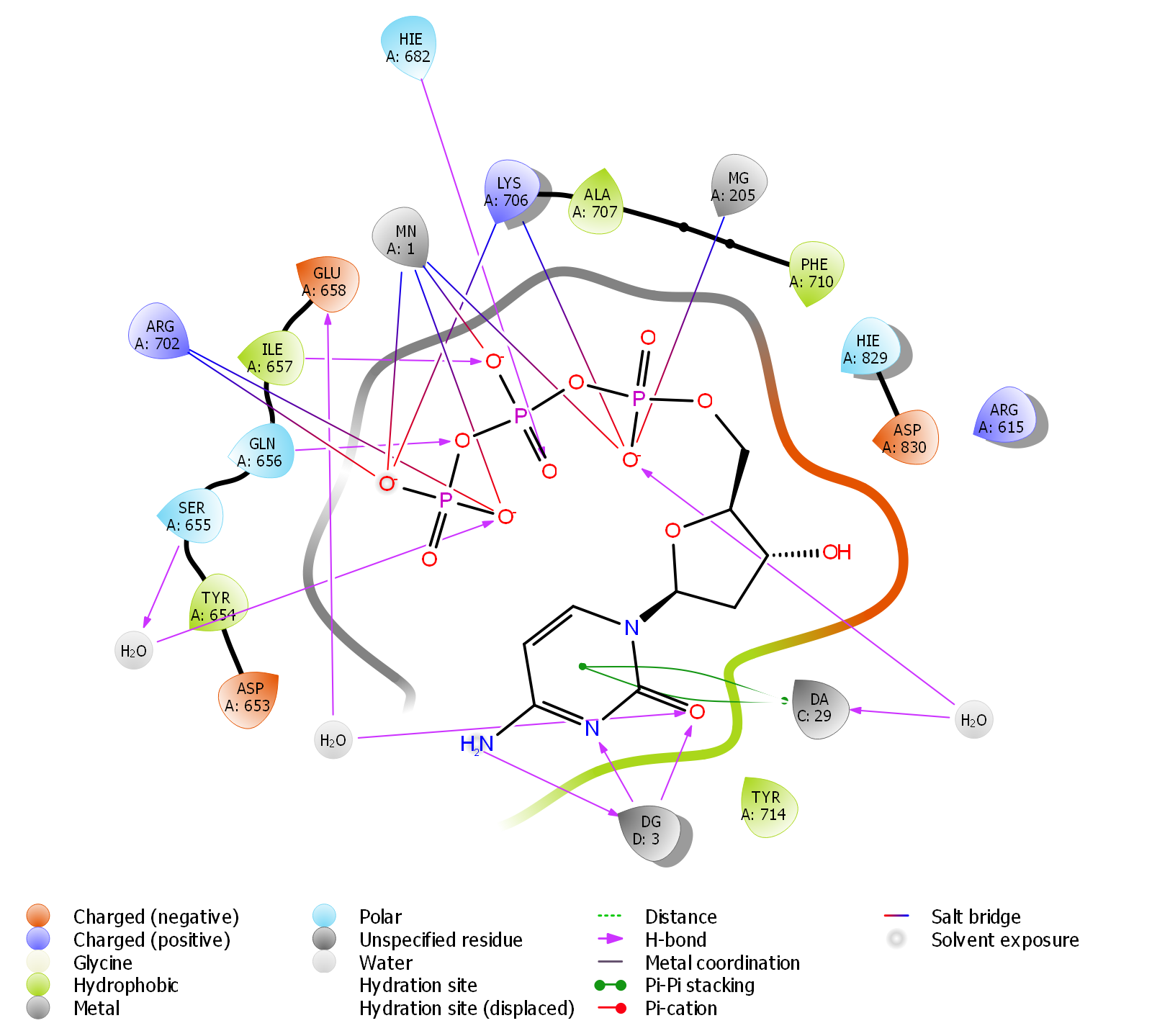

Supplement: Supplementary file 1 [file mmc1.zip › Ca_Cd.png]

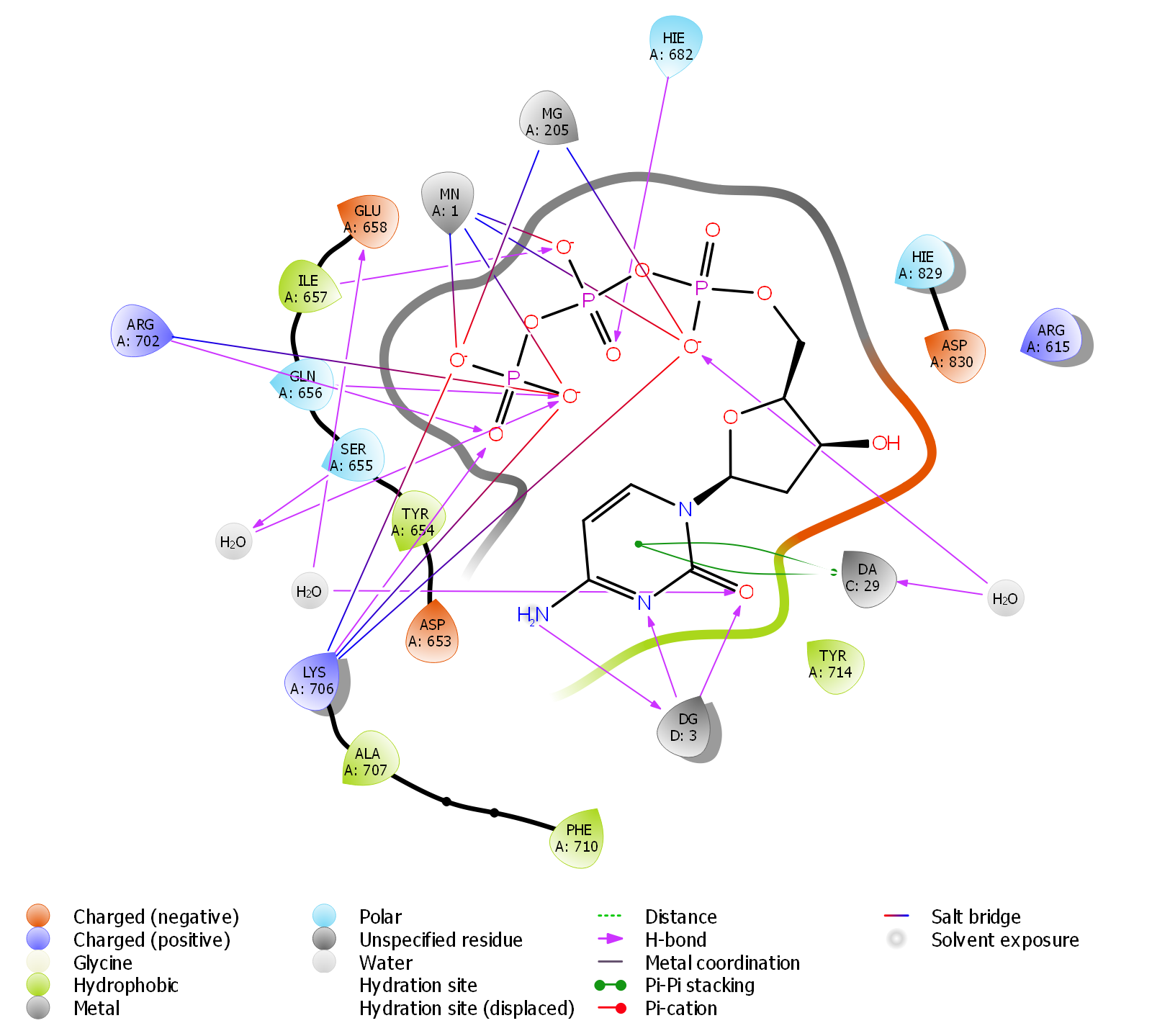

Supplement: Supplementary file 1 [file mmc1.zip › Co_Cd.png]

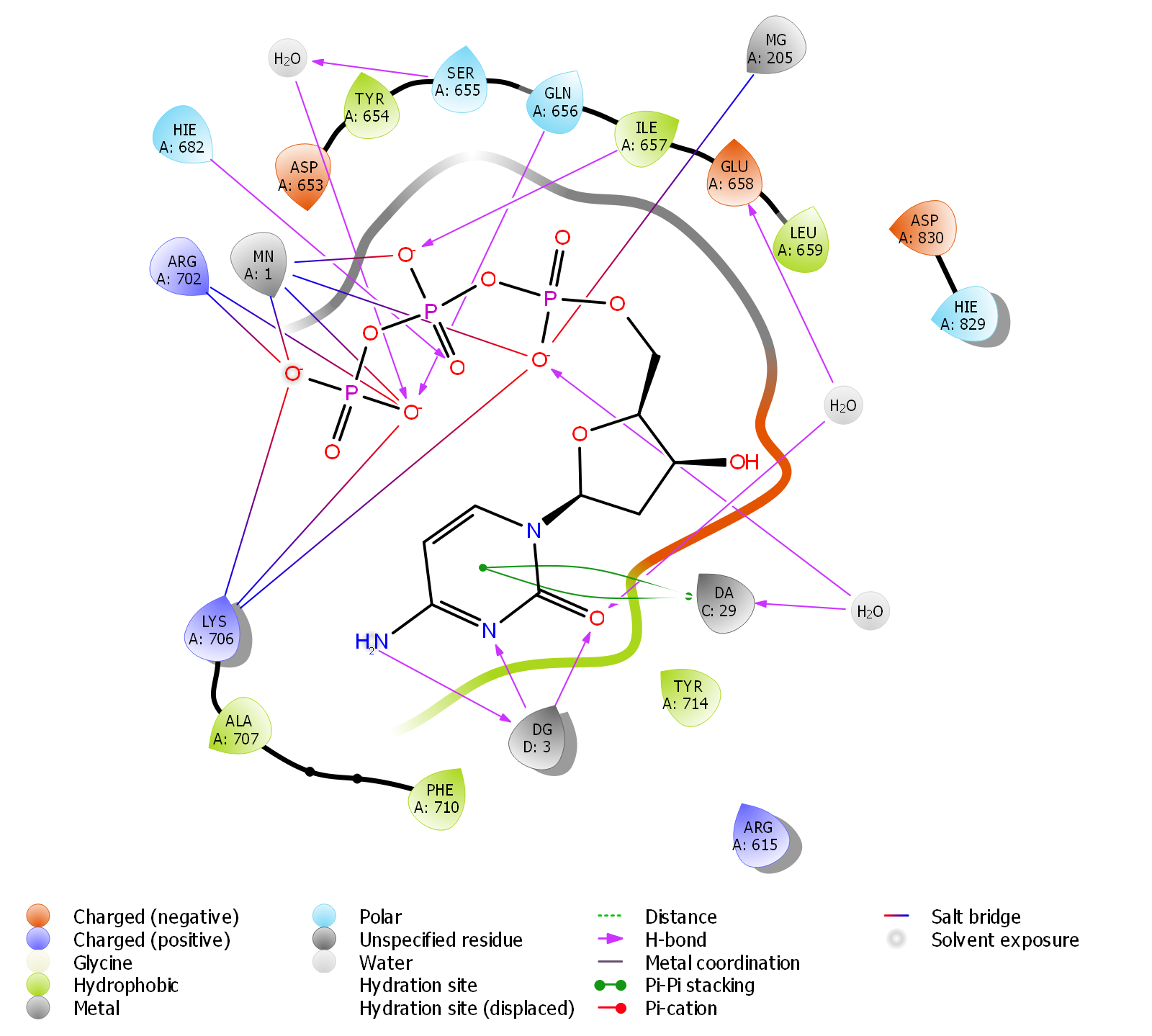

Supplement: Supplementary file 1 [file mmc1.zip › Cu_Cd.png]

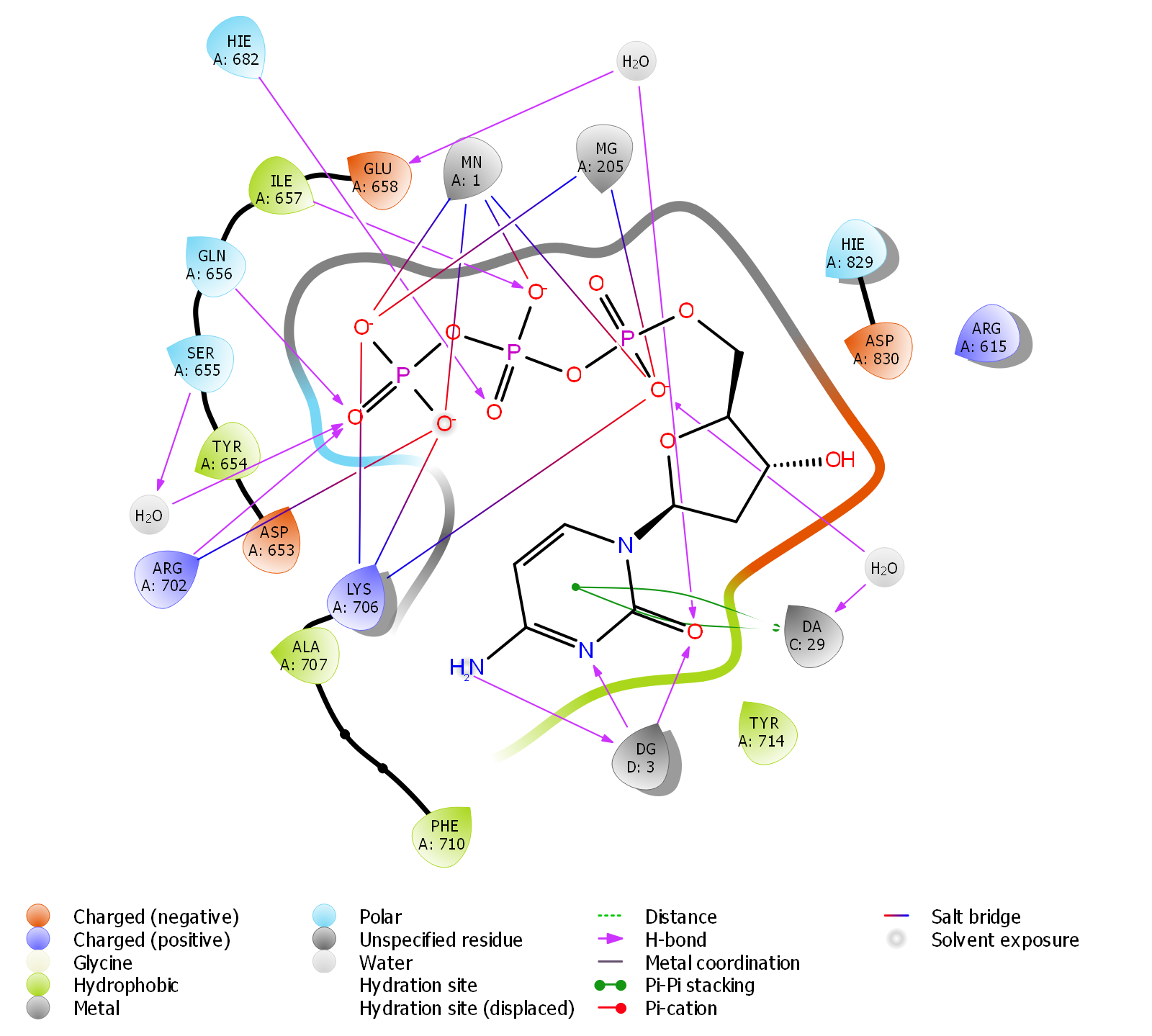

Supplement: Supplementary file 1 [file mmc1.zip › Mg_Cd.png]

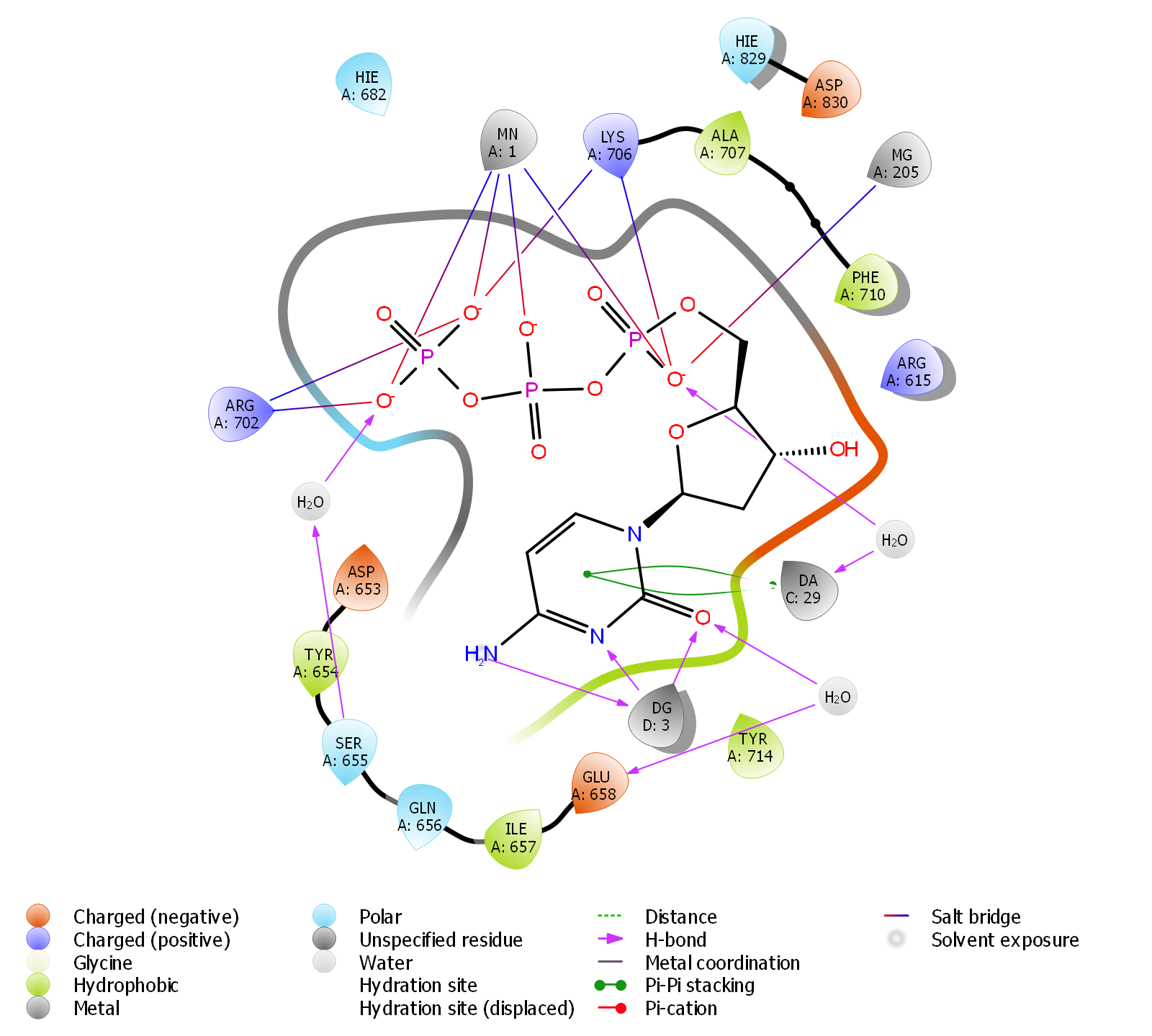

Supplement: Supplementary file 1 [file mmc1.zip › Mn_Cd.png]

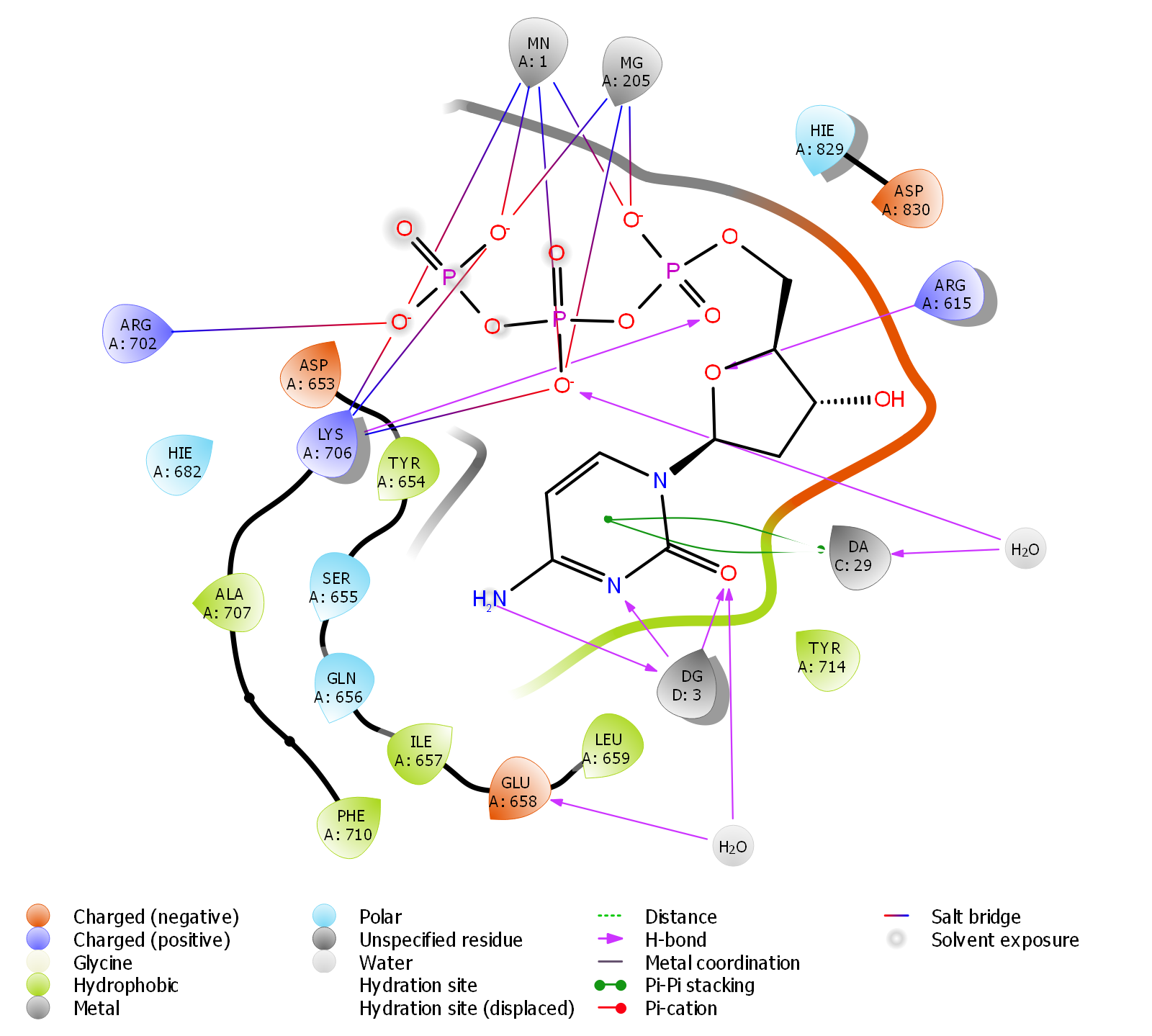

Supplement: Supplementary file 1 [file mmc1.zip › Ni_Cd.png]

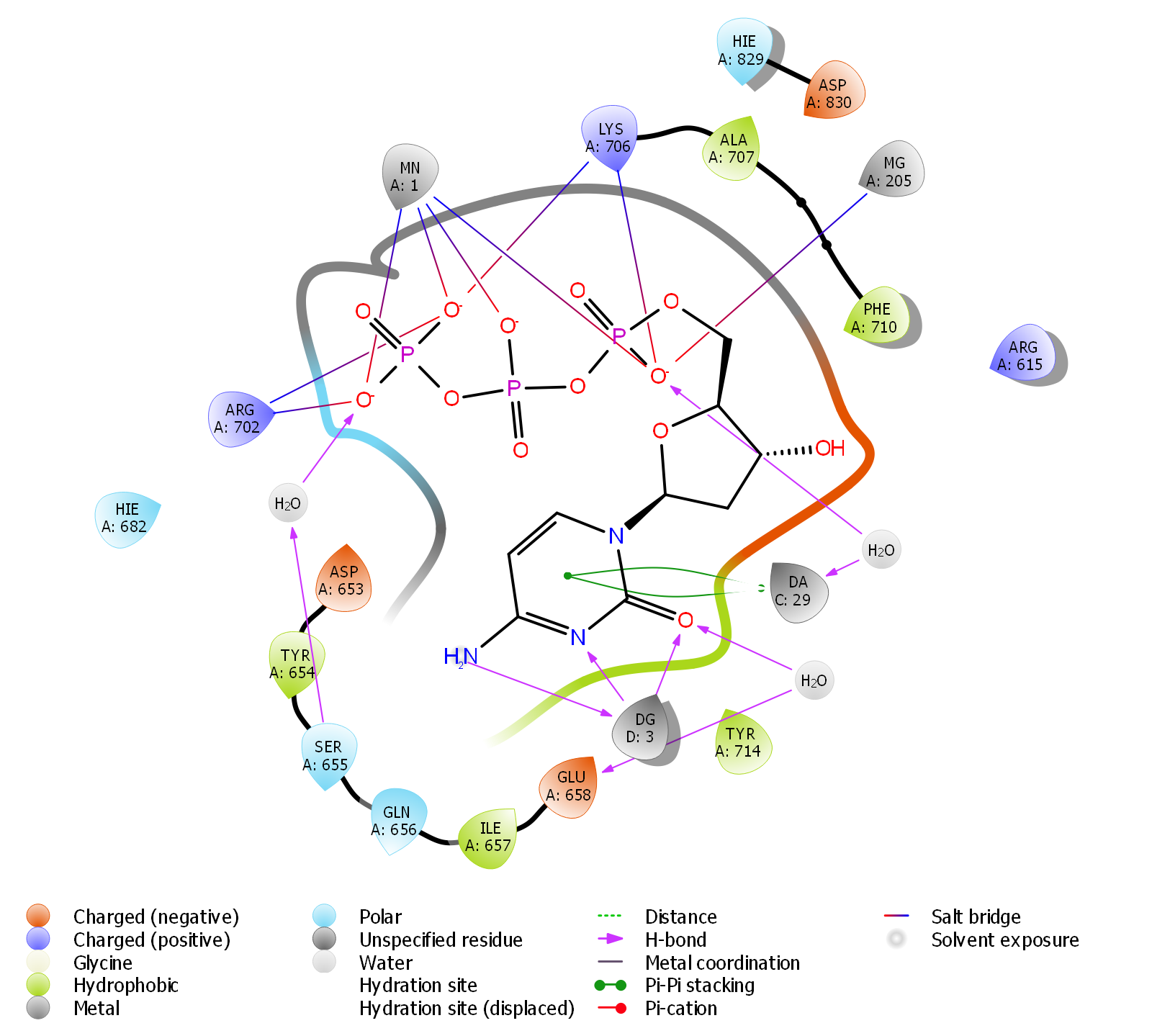

Supplement: Supplementary file 1 [file mmc1.zip › Zn_Cd.png]

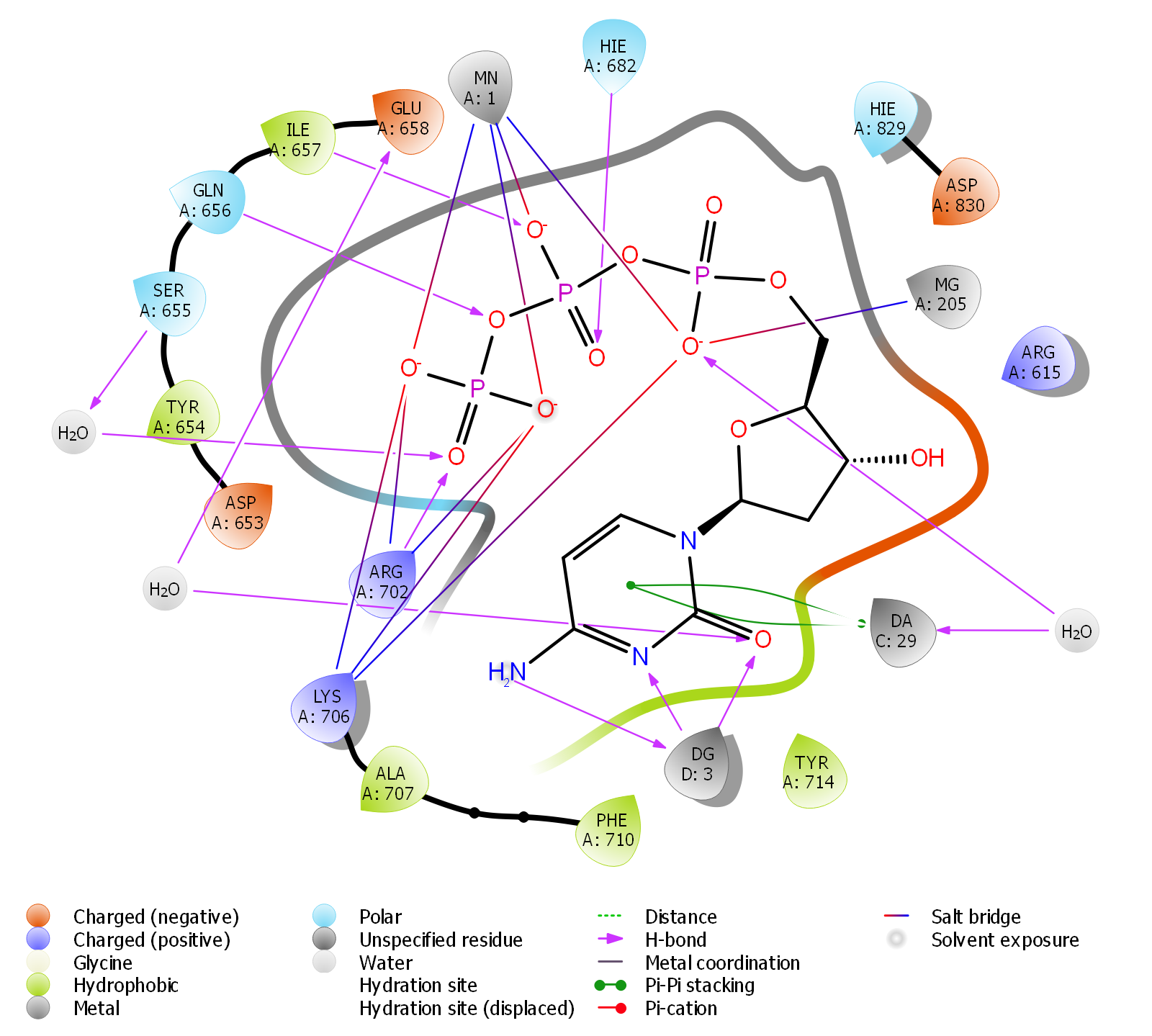

Supplement: Supplementary file 1 [file mmc1.zip › Ca_Co.png]

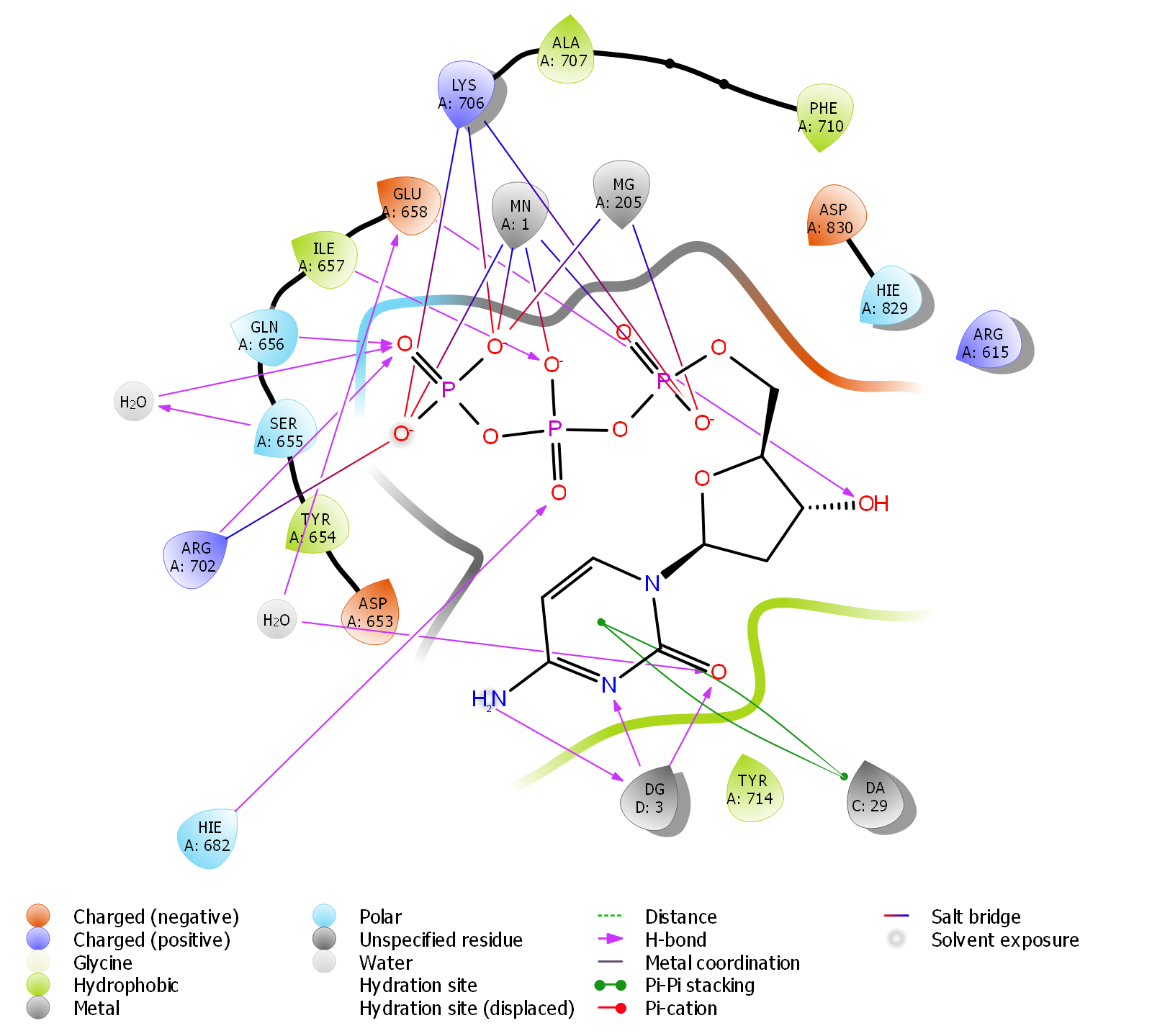

Supplement: Supplementary file 1 [file mmc1.zip › Cd_Co.png]

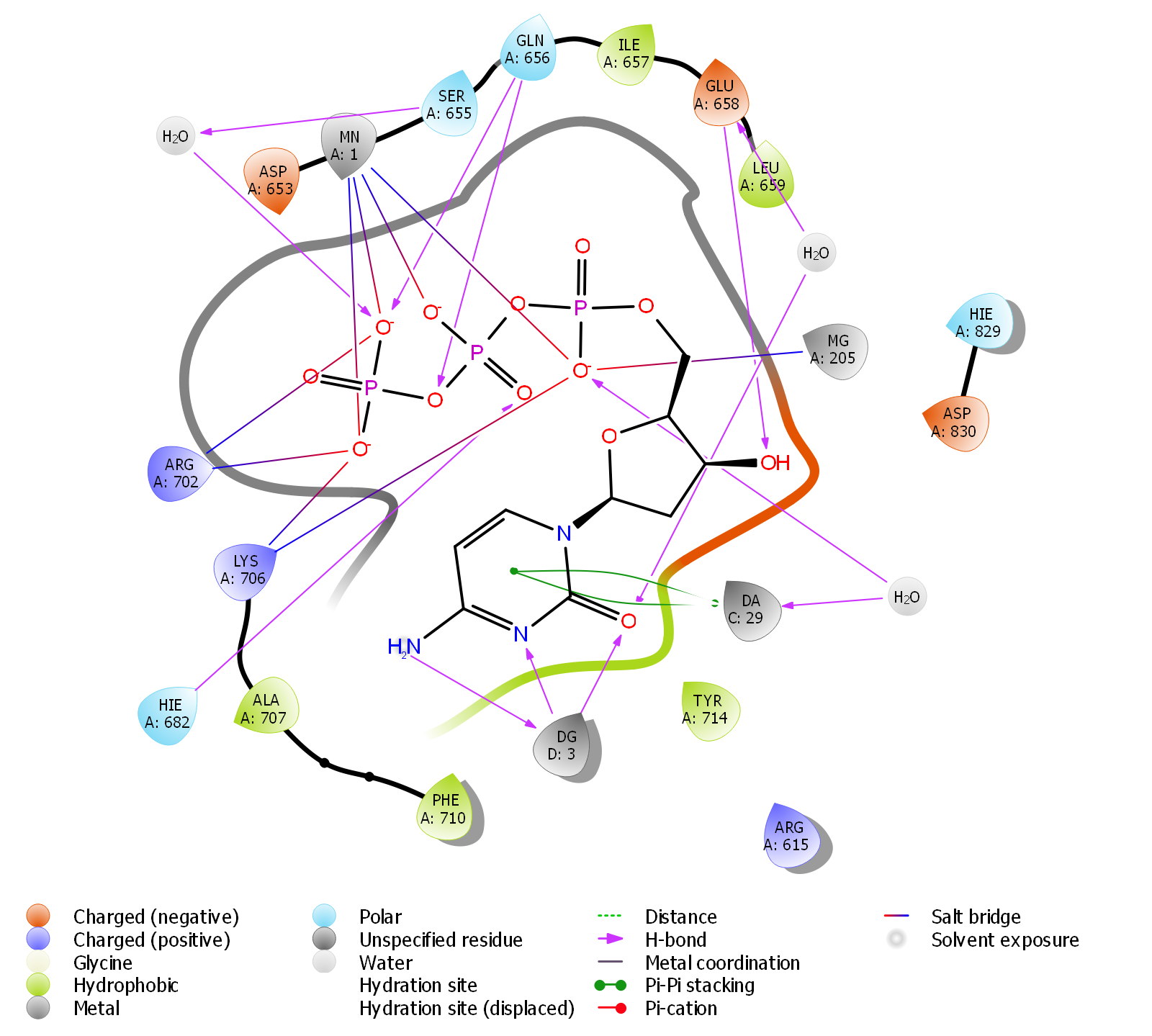

Supplement: Supplementary file 1 [file mmc1.zip › Cu_Co.png]

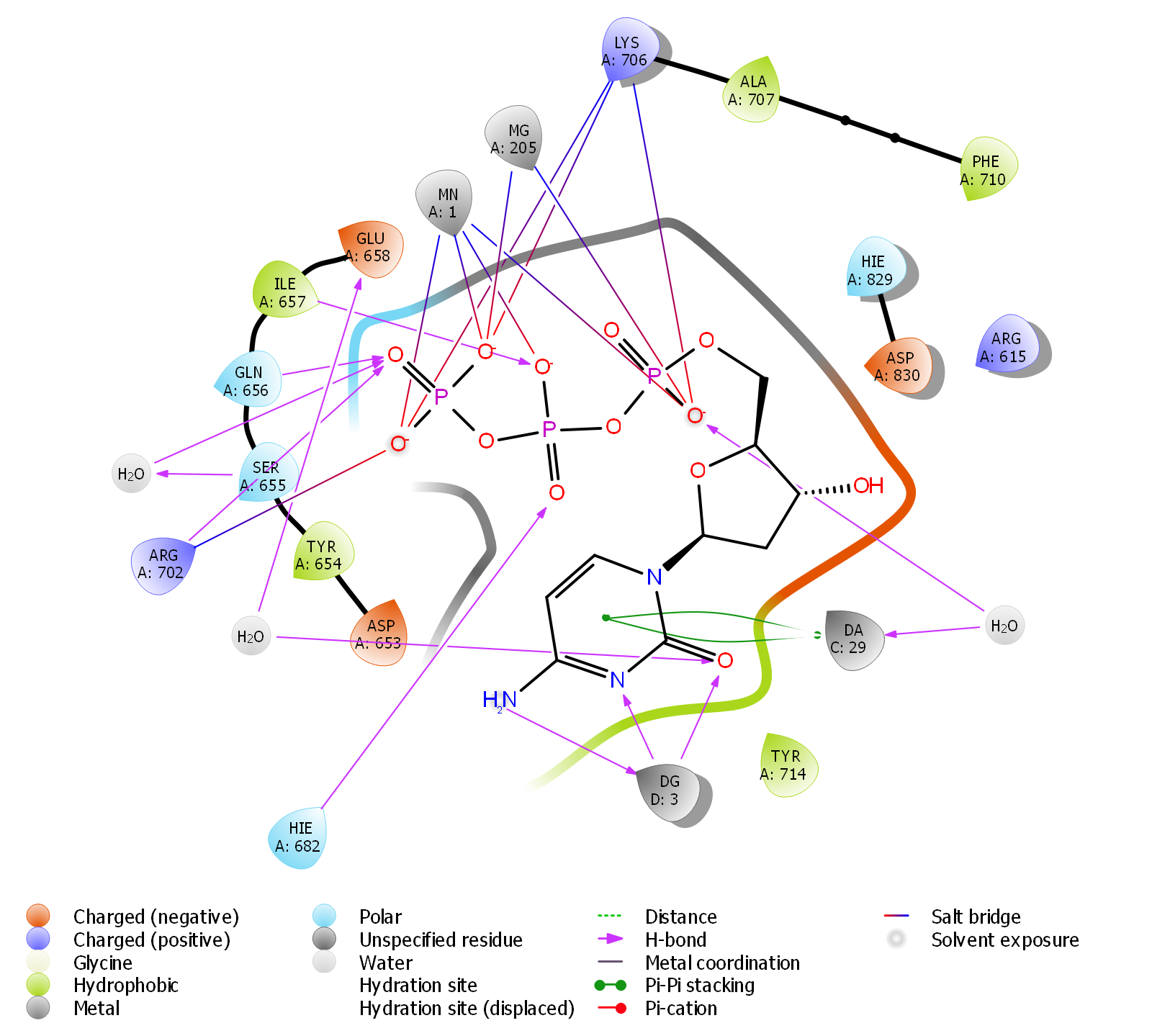

Supplement: Supplementary file 1 [file mmc1.zip › Mg_Co.png]

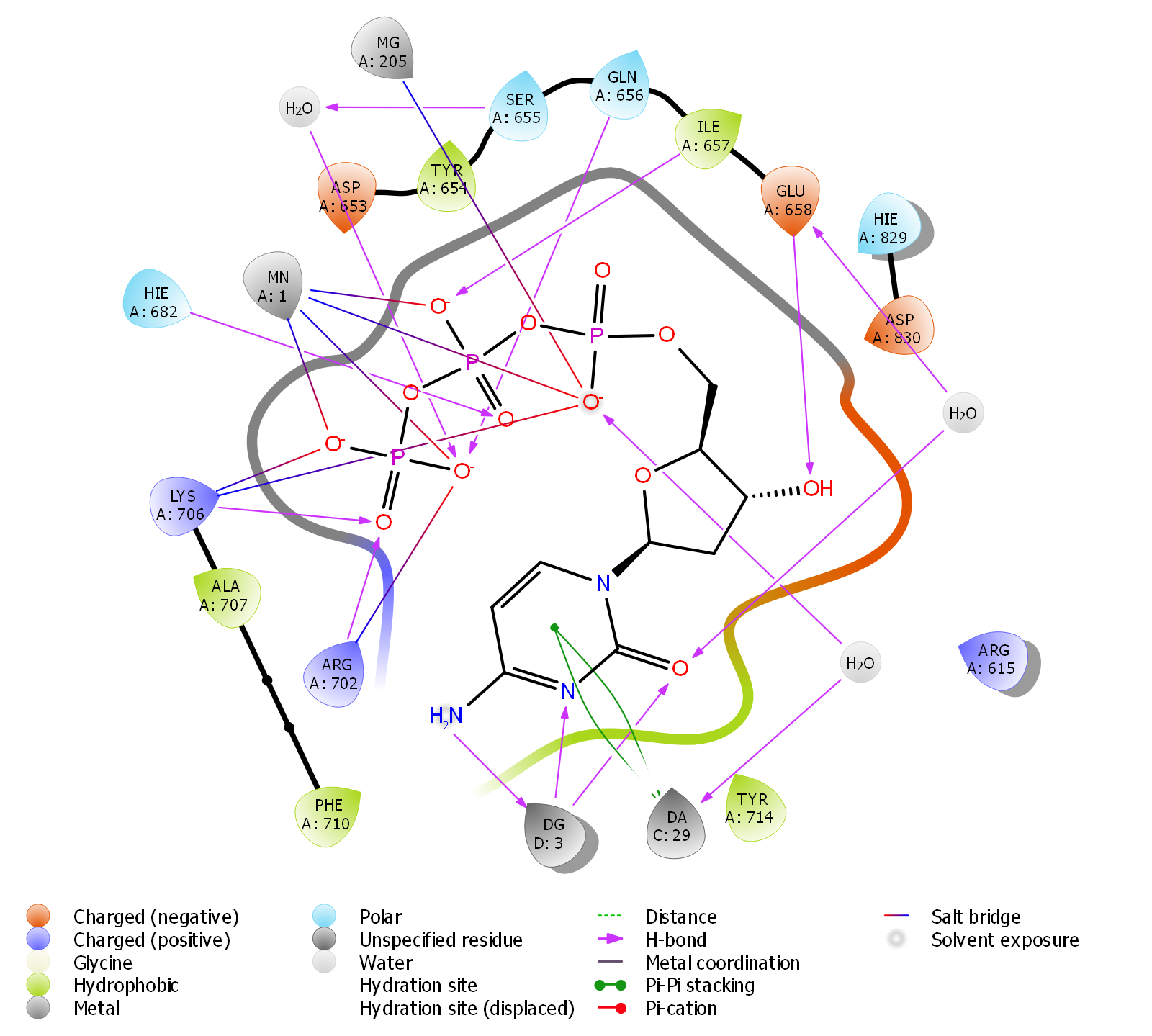

Supplement: Supplementary file 1 [file mmc1.zip › Mn_Co.png]

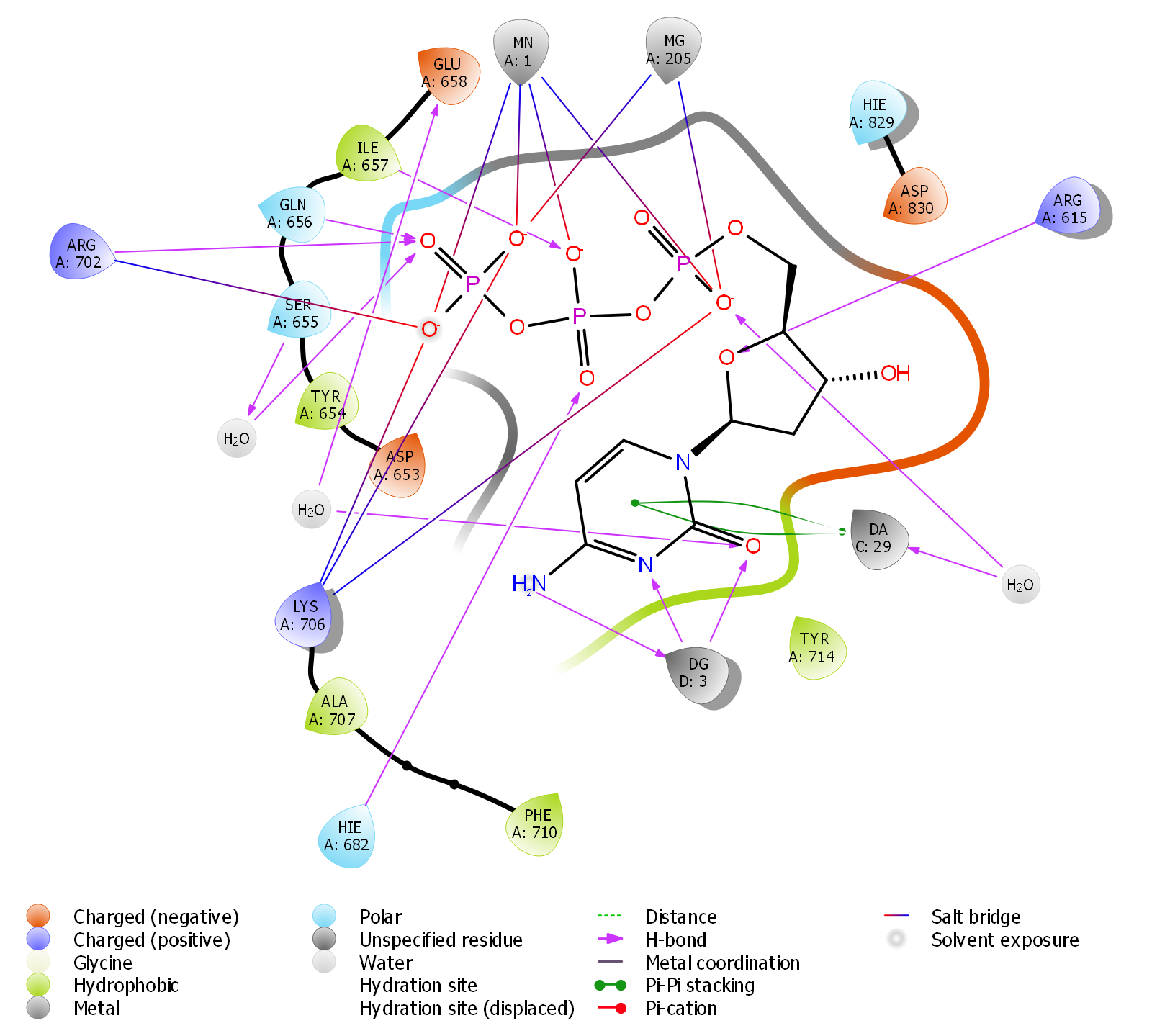

Supplement: Supplementary file 1 [file mmc1.zip › Ni_Co.png]

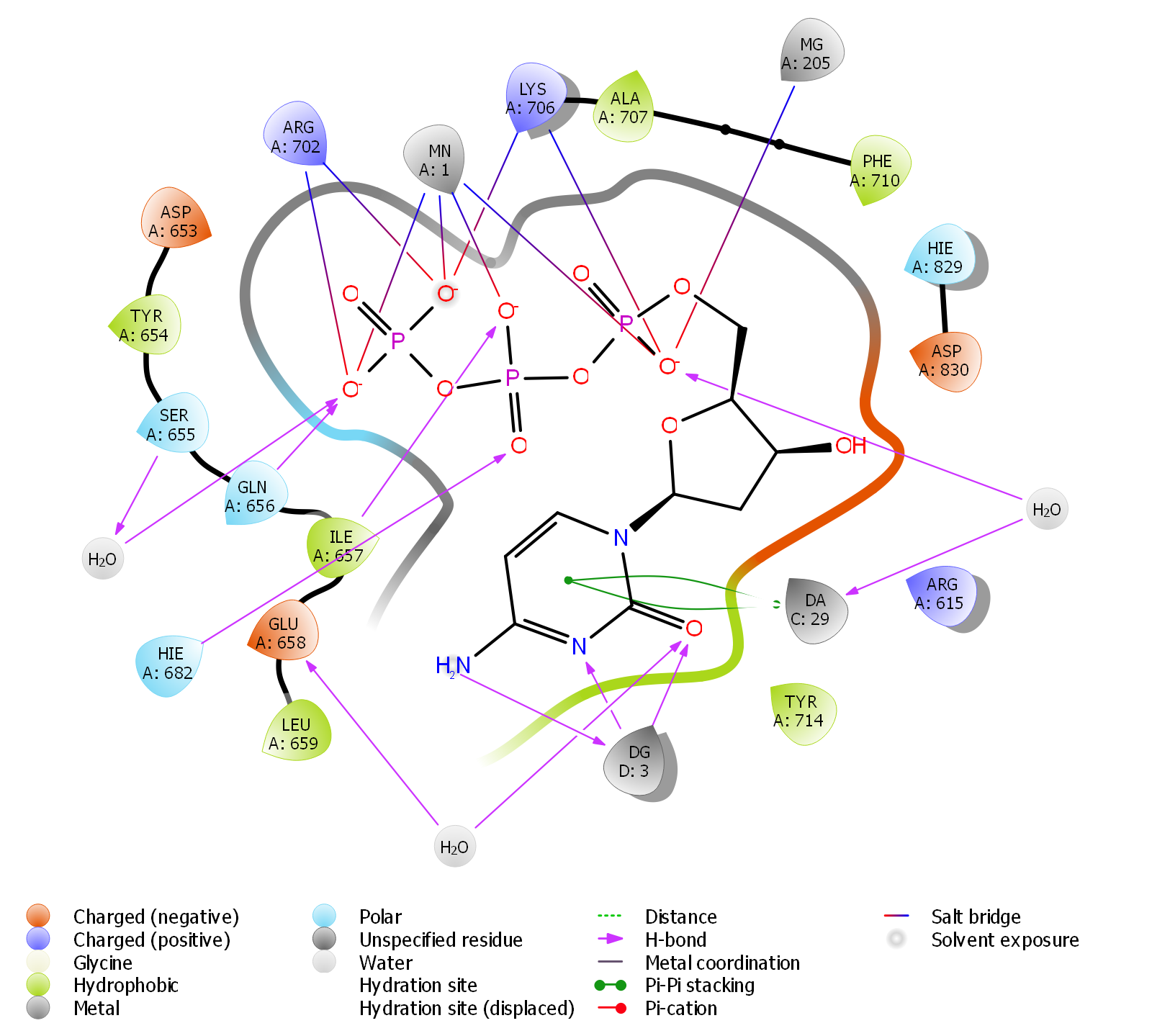

Supplement: Supplementary file 1 [file mmc1.zip › Zn_Co.png]

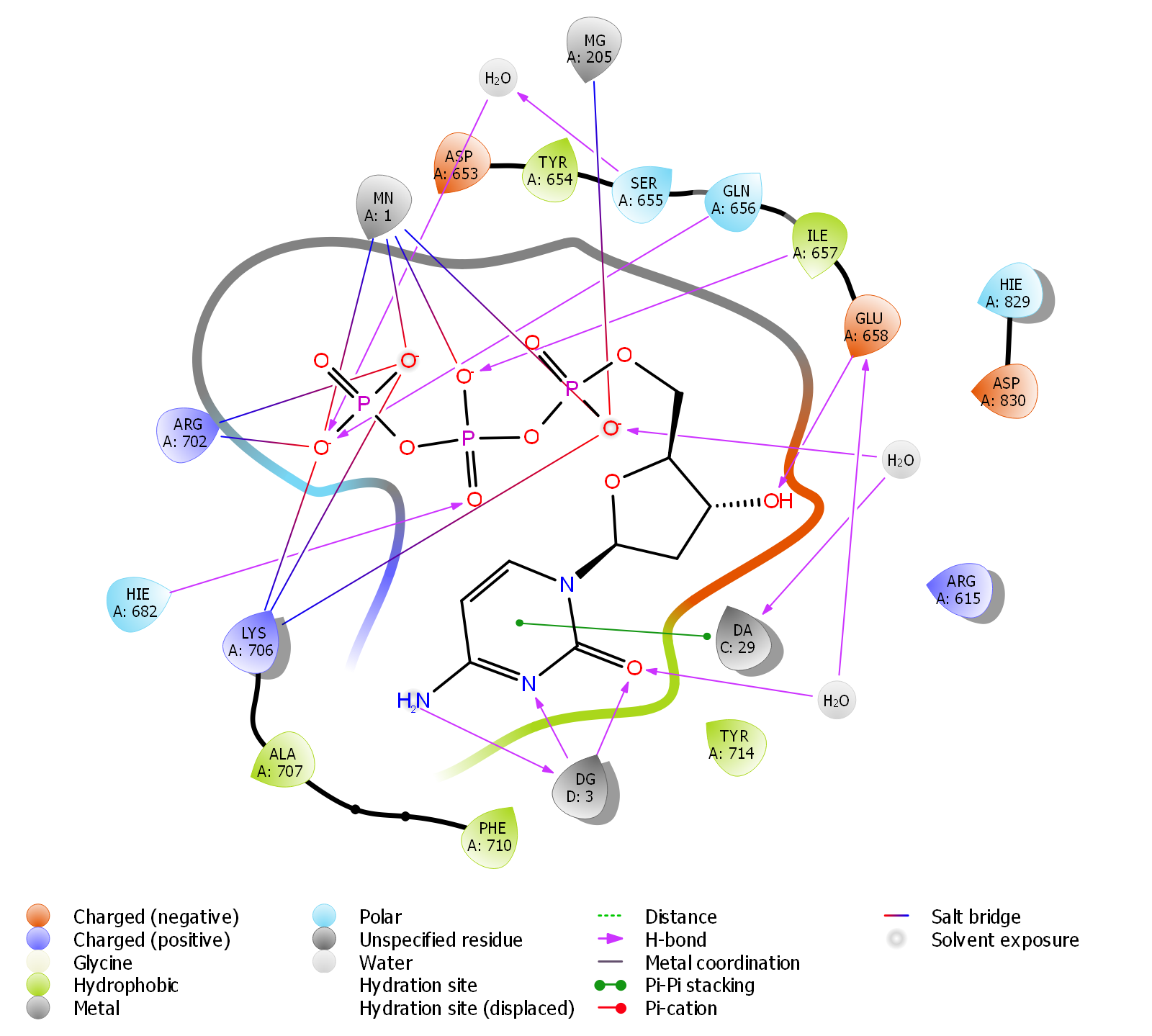

Supplement: Supplementary file 1 [file mmc1.zip › Ca_Cu.png]

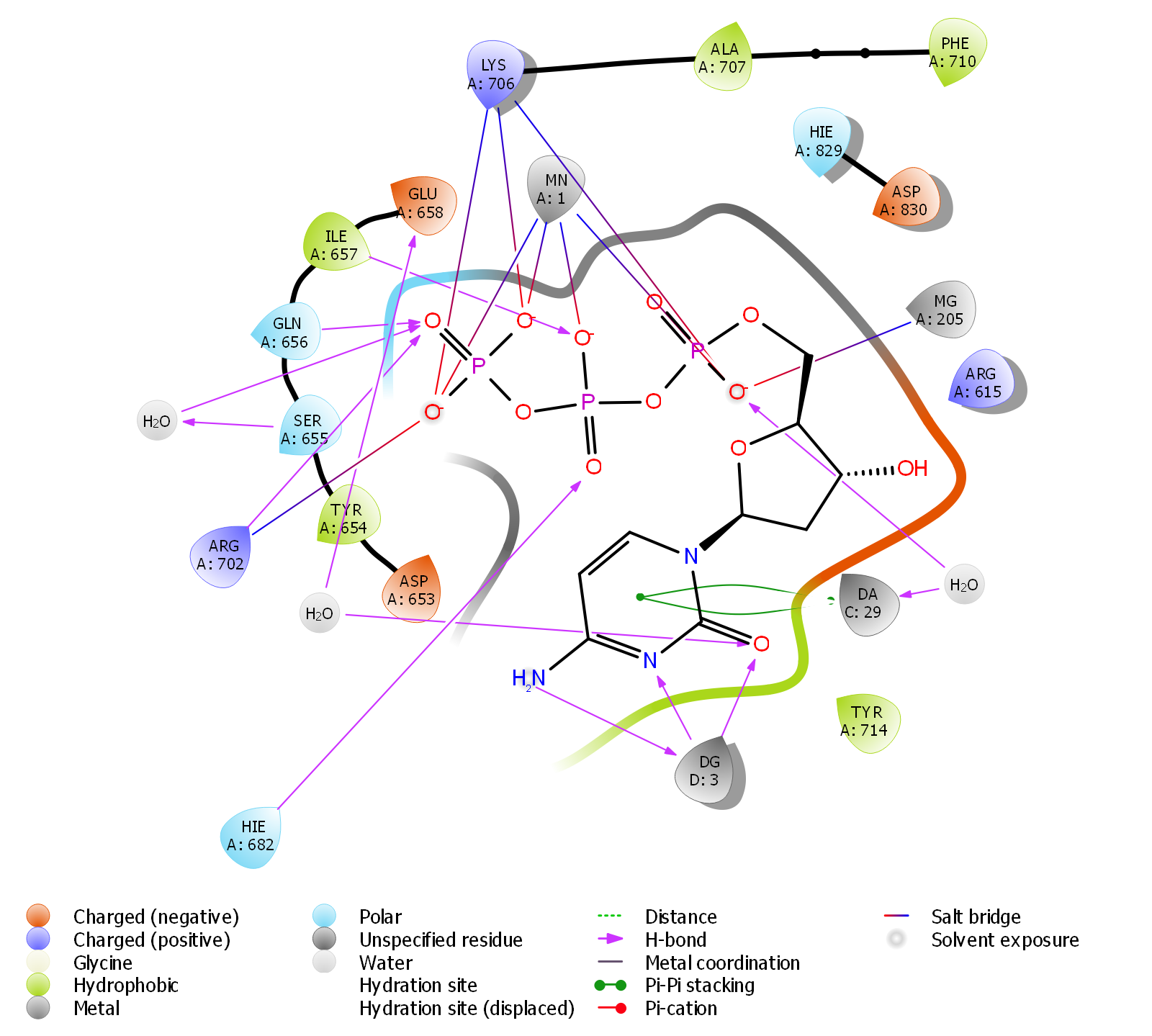

Supplement: Supplementary file 1 [file mmc1.zip › Cd_Cu.png]

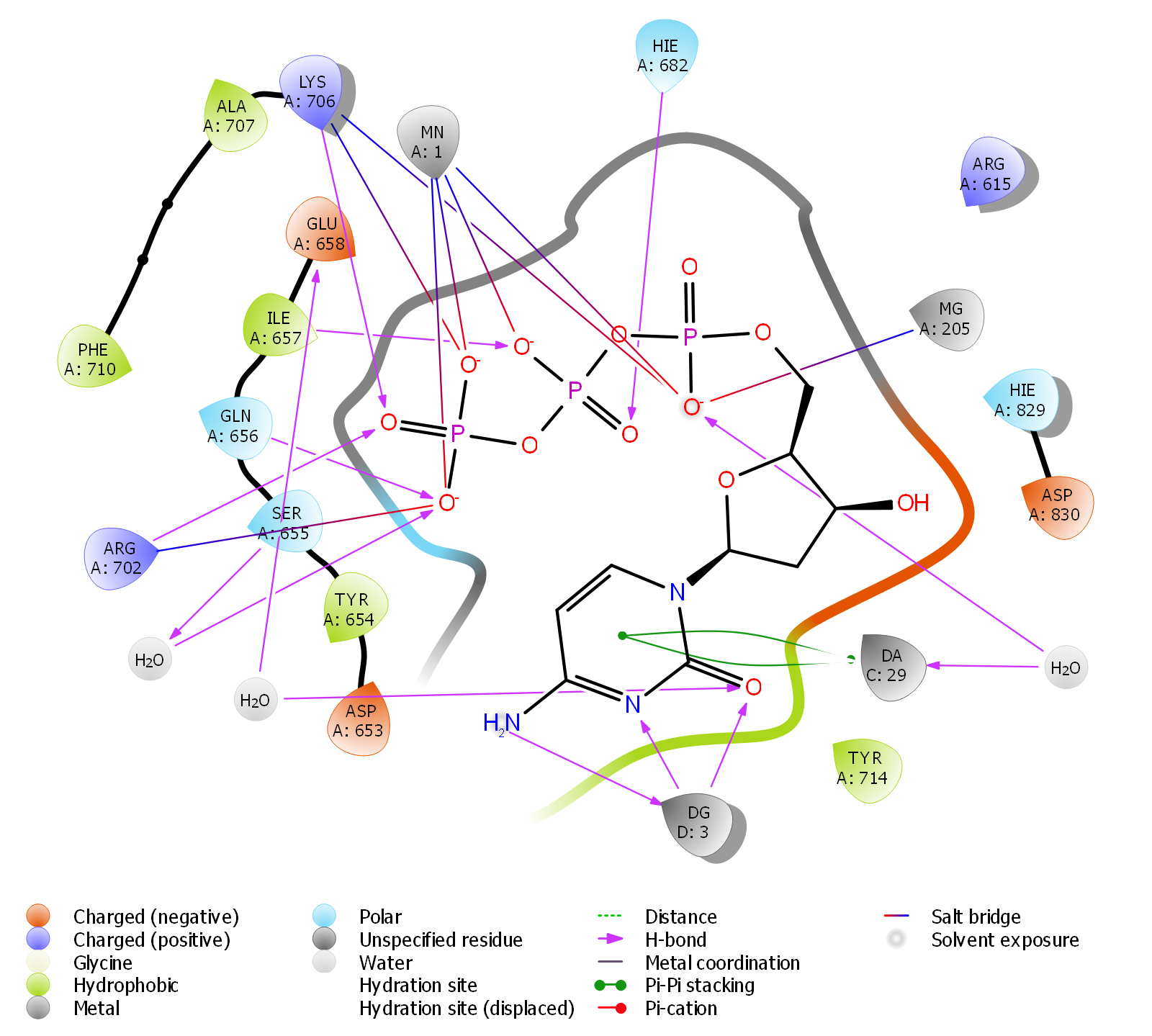

Supplement: Supplementary file 1 [file mmc1.zip › Co_Cu.png]

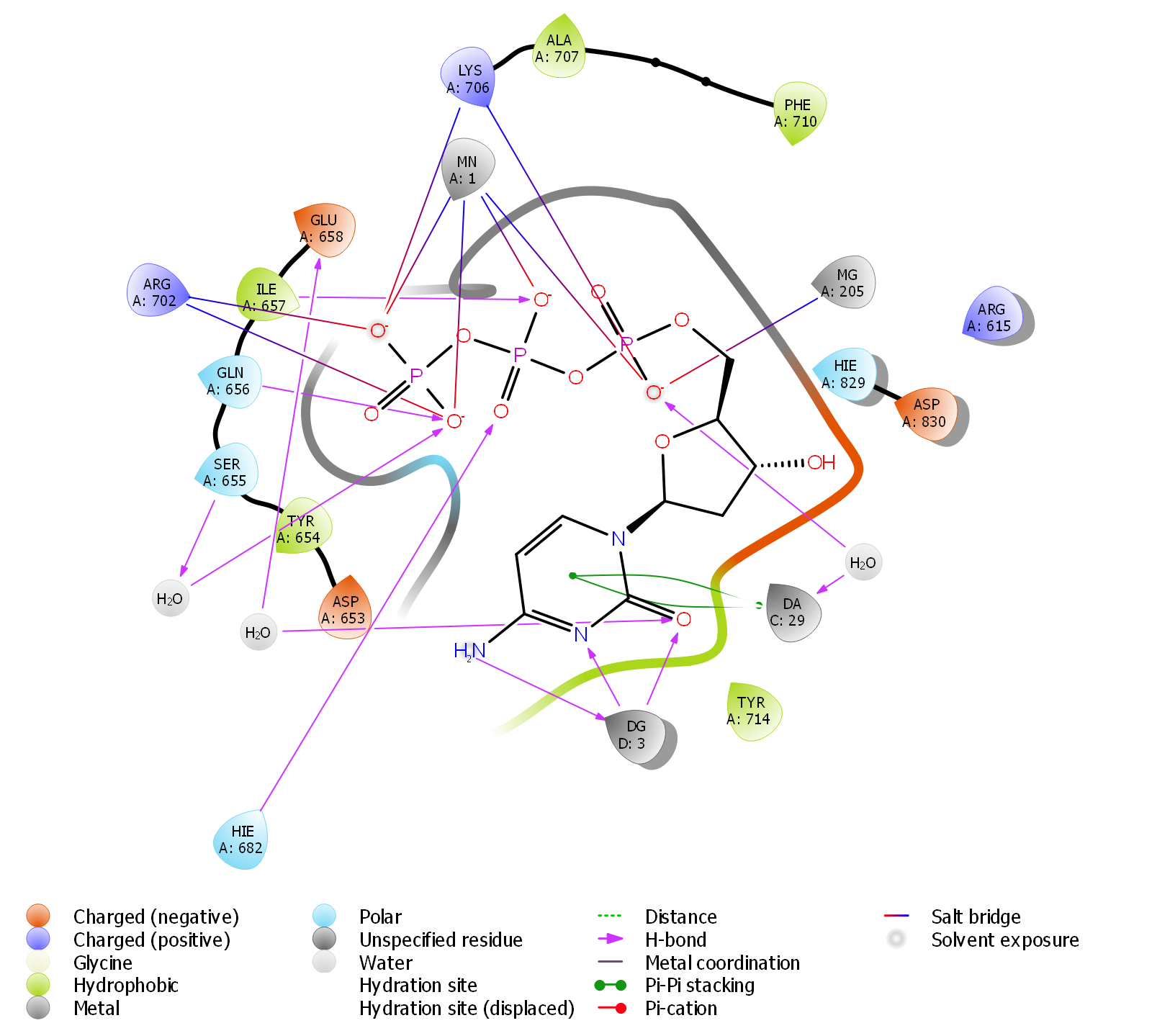

Supplement: Supplementary file 1 [file mmc1.zip › Mg_Cu.png]

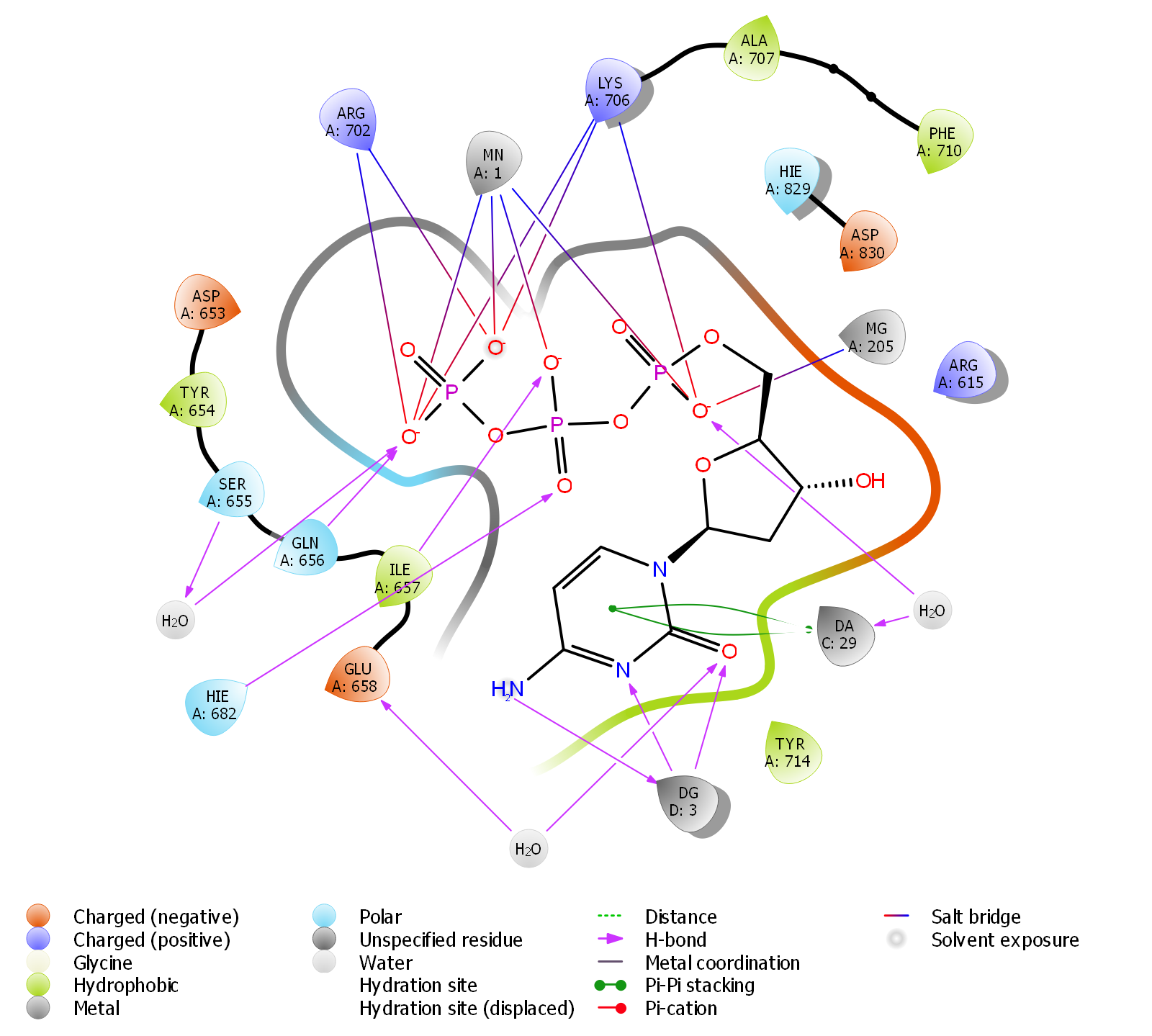

Supplement: Supplementary file 1 [file mmc1.zip › Mn_Cu.png]

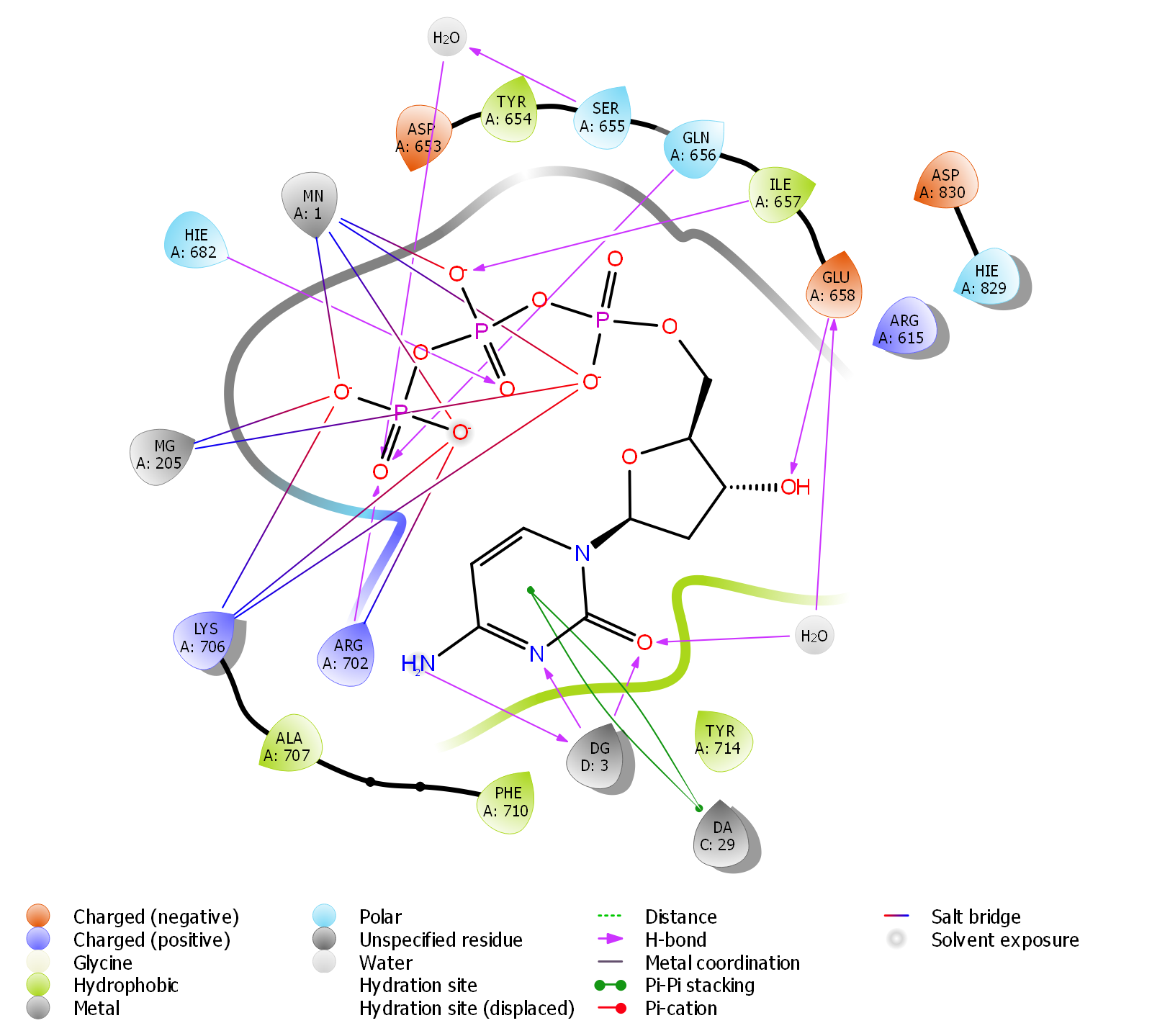

Supplement: Supplementary file 1 [file mmc1.zip › Ni_Cu.png]

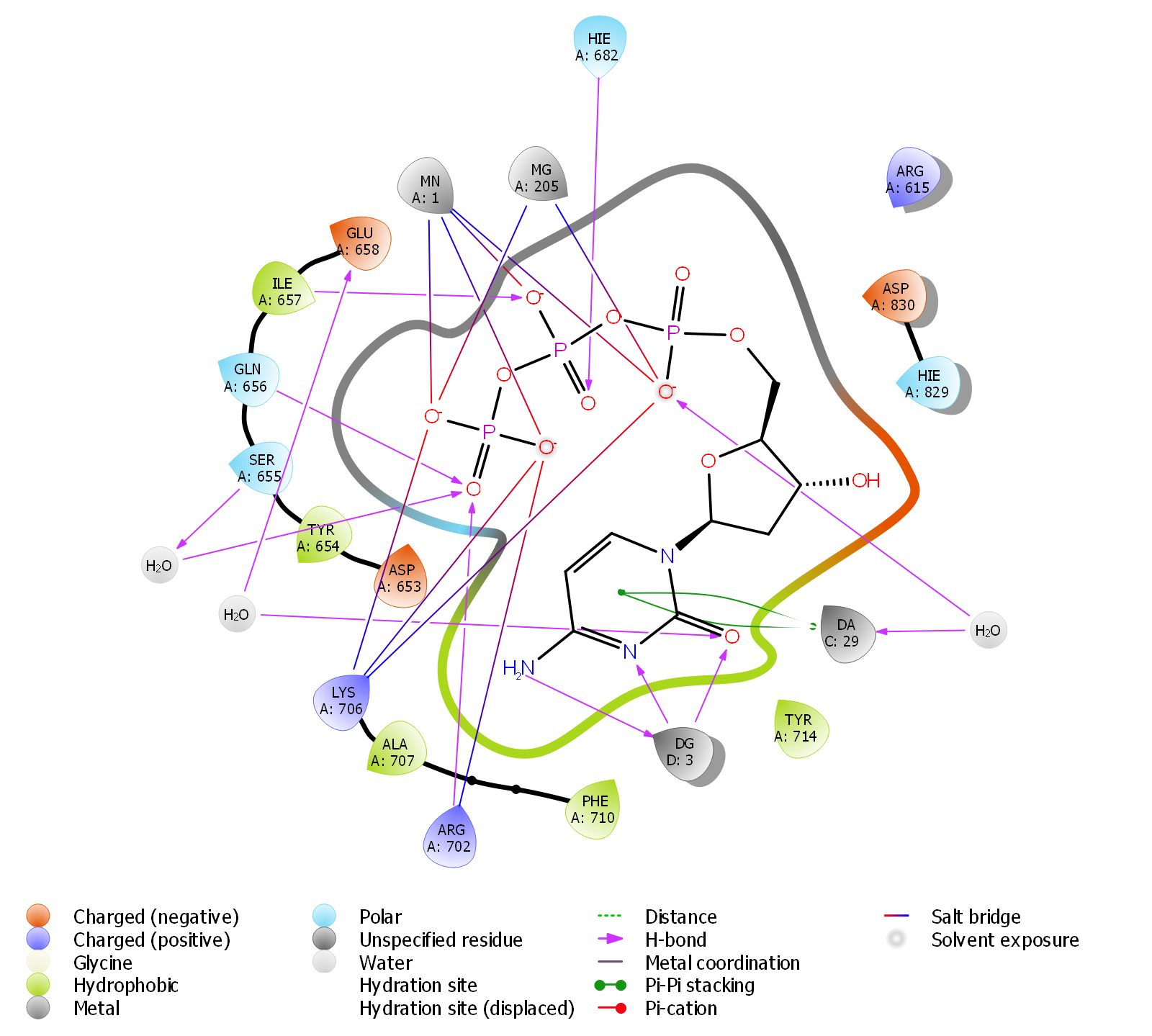

Supplement: Supplementary file 1 [file mmc1.zip › Zn_Cu.png]

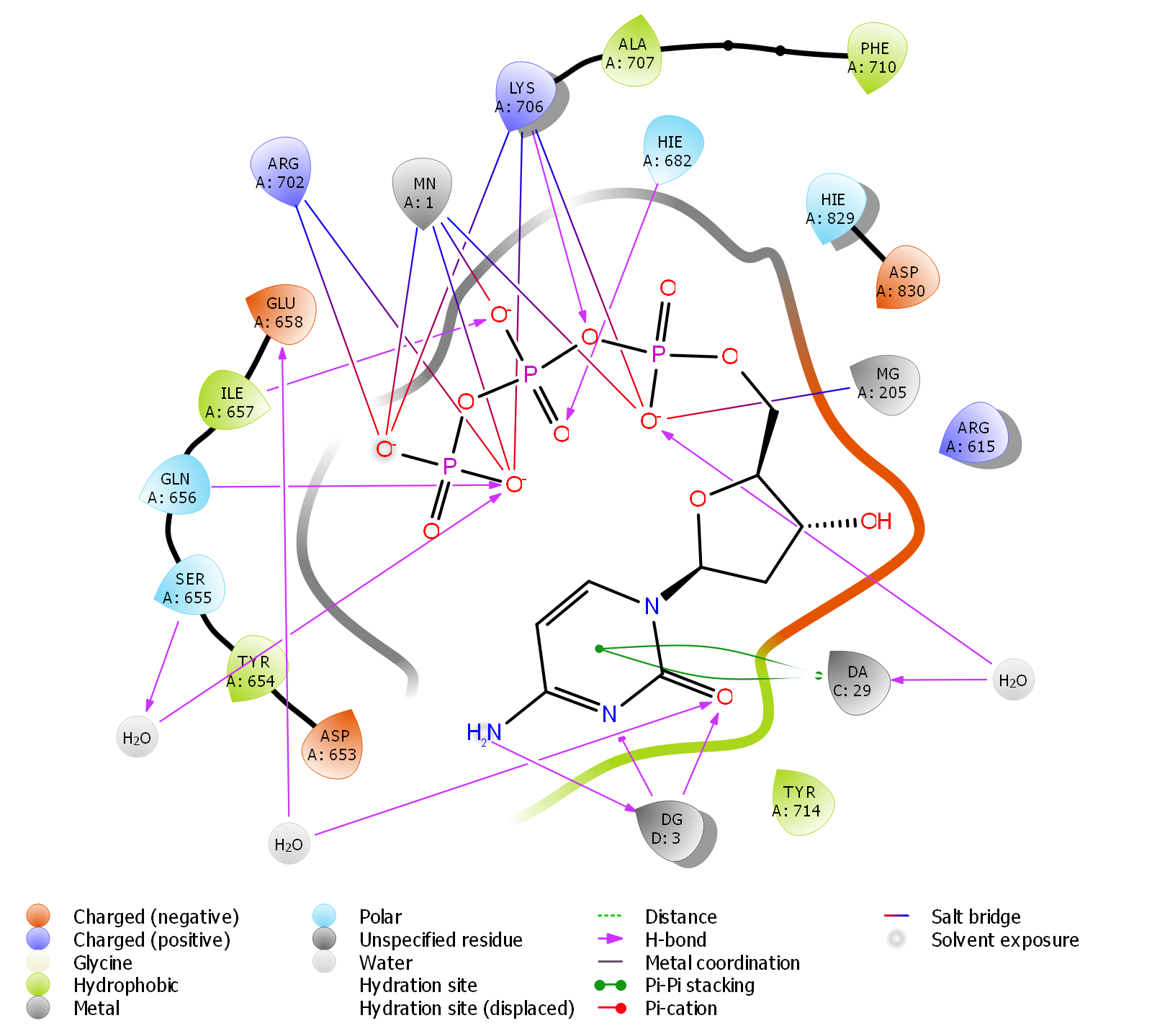

Supplement: Supplementary file 1 [file mmc1.zip › Ca_Mn.png]

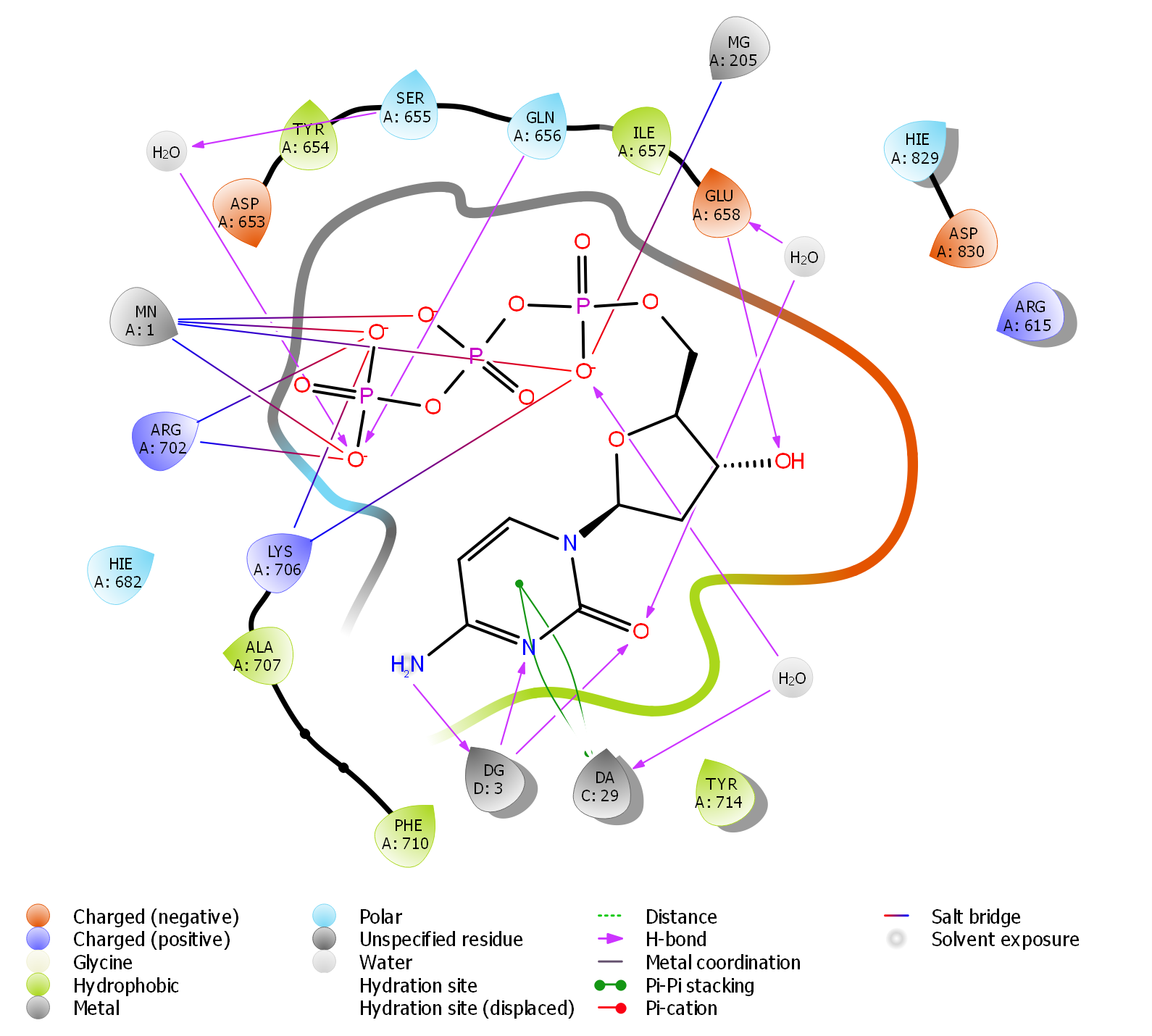

Supplement: Supplementary file 1 [file mmc1.zip › Cd_Mn.png]

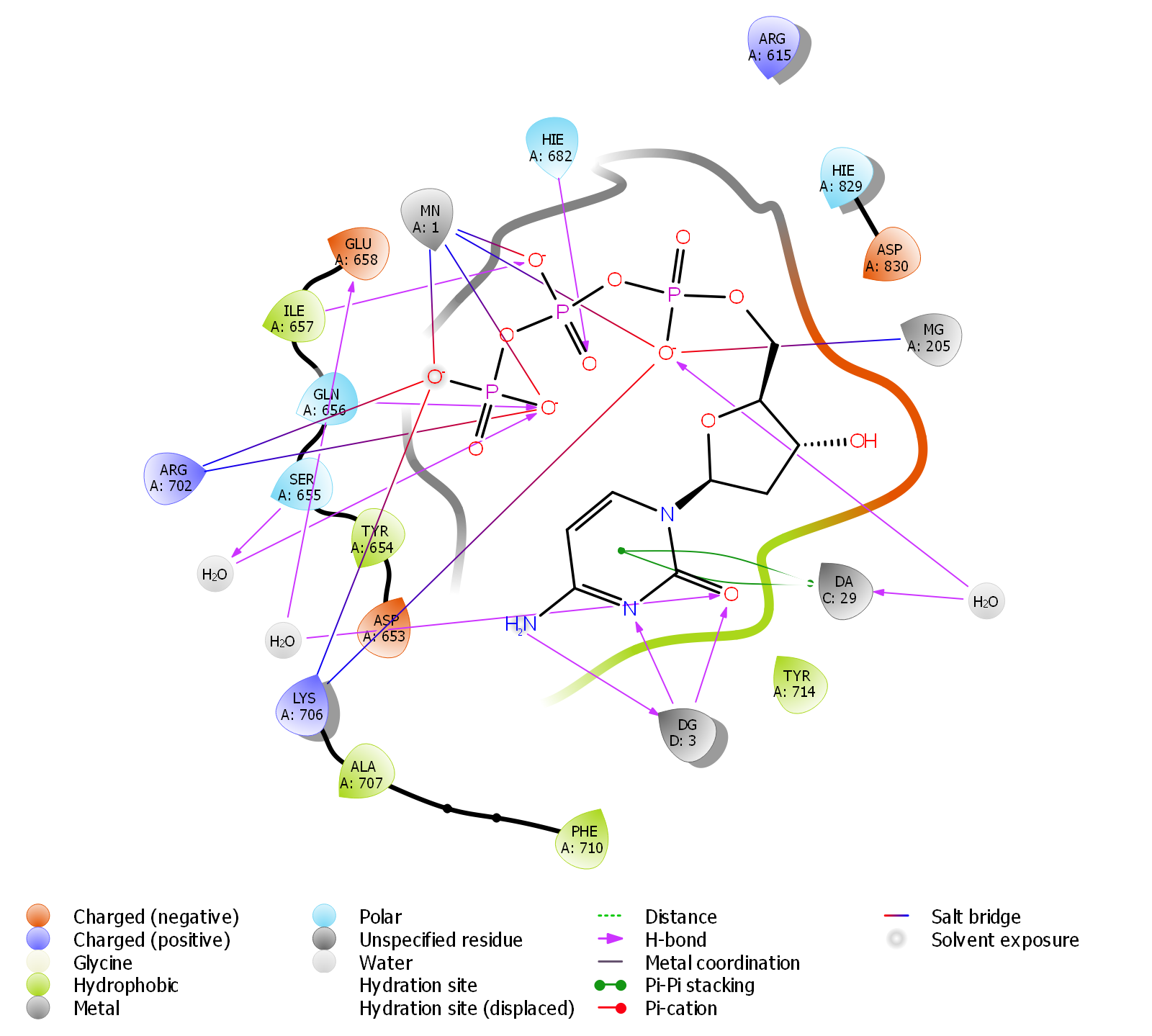

Supplement: Supplementary file 1 [file mmc1.zip › Co_Mn.png]

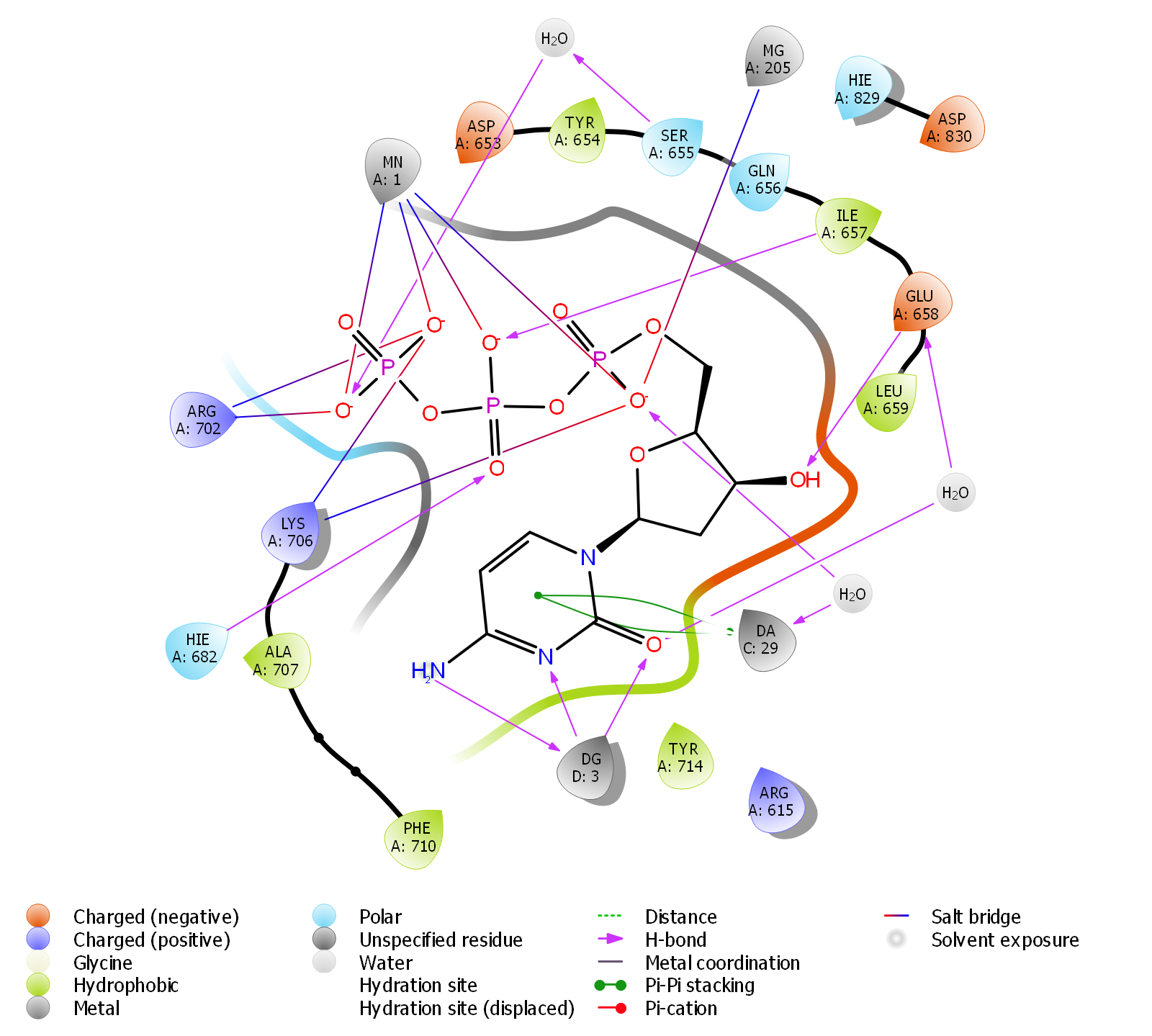

Supplement: Supplementary file 1 [file mmc1.zip › Cu_Mn.png]

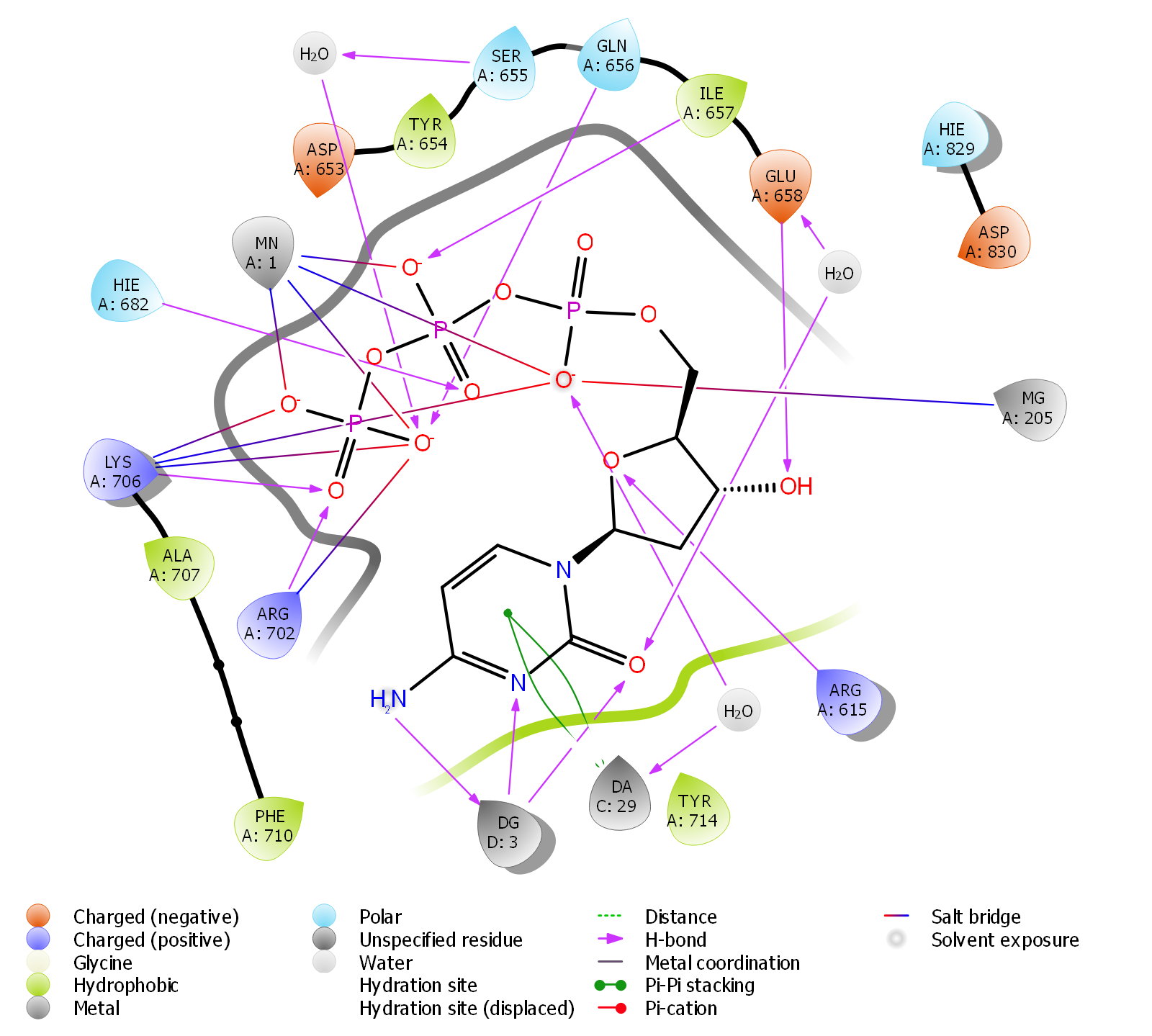

Supplement: Supplementary file 1 [file mmc1.zip › Ni_Mn.png]

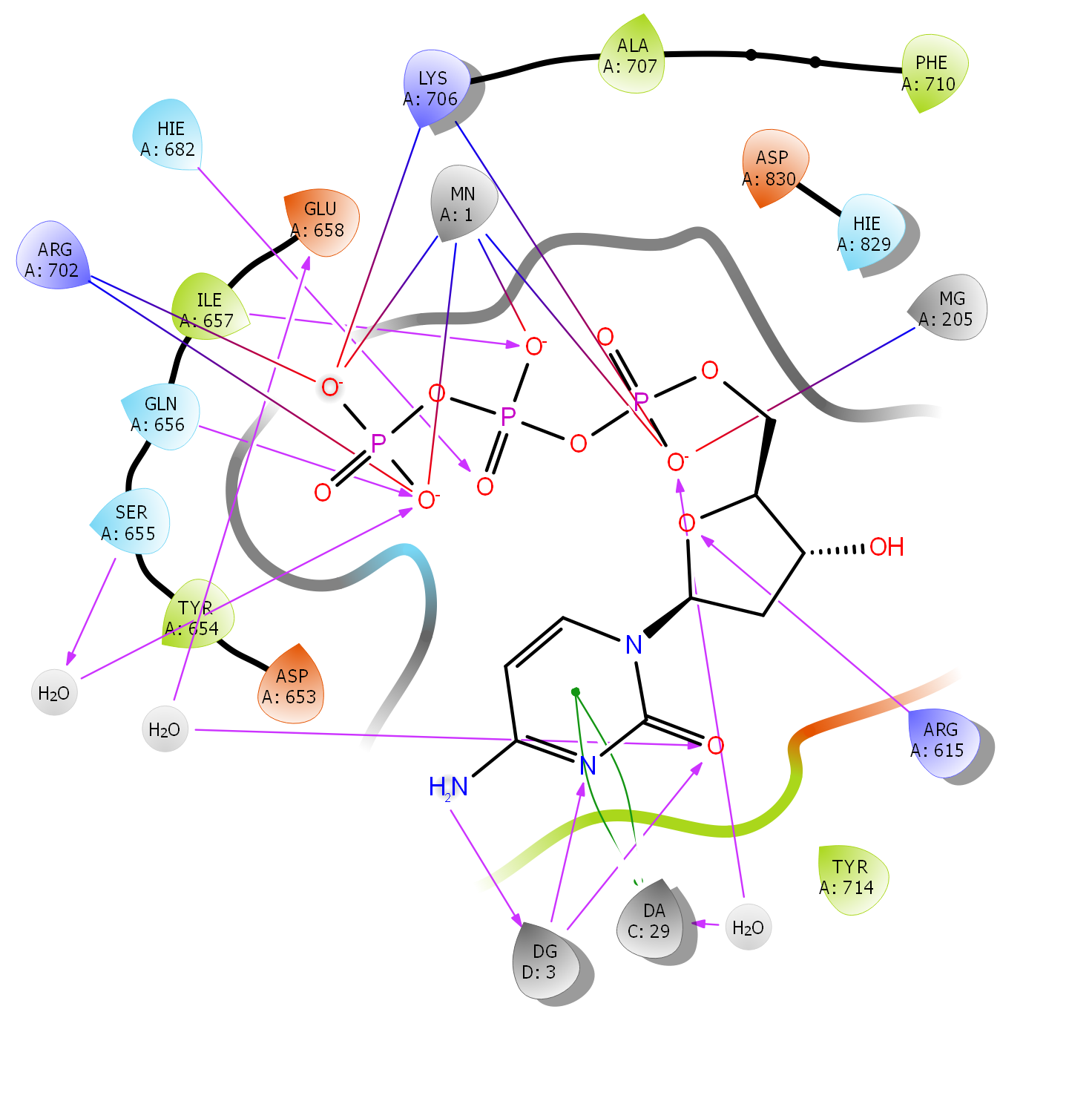

Supplement: Supplementary file 1 [file mmc1.zip › Zn_Mn.png]

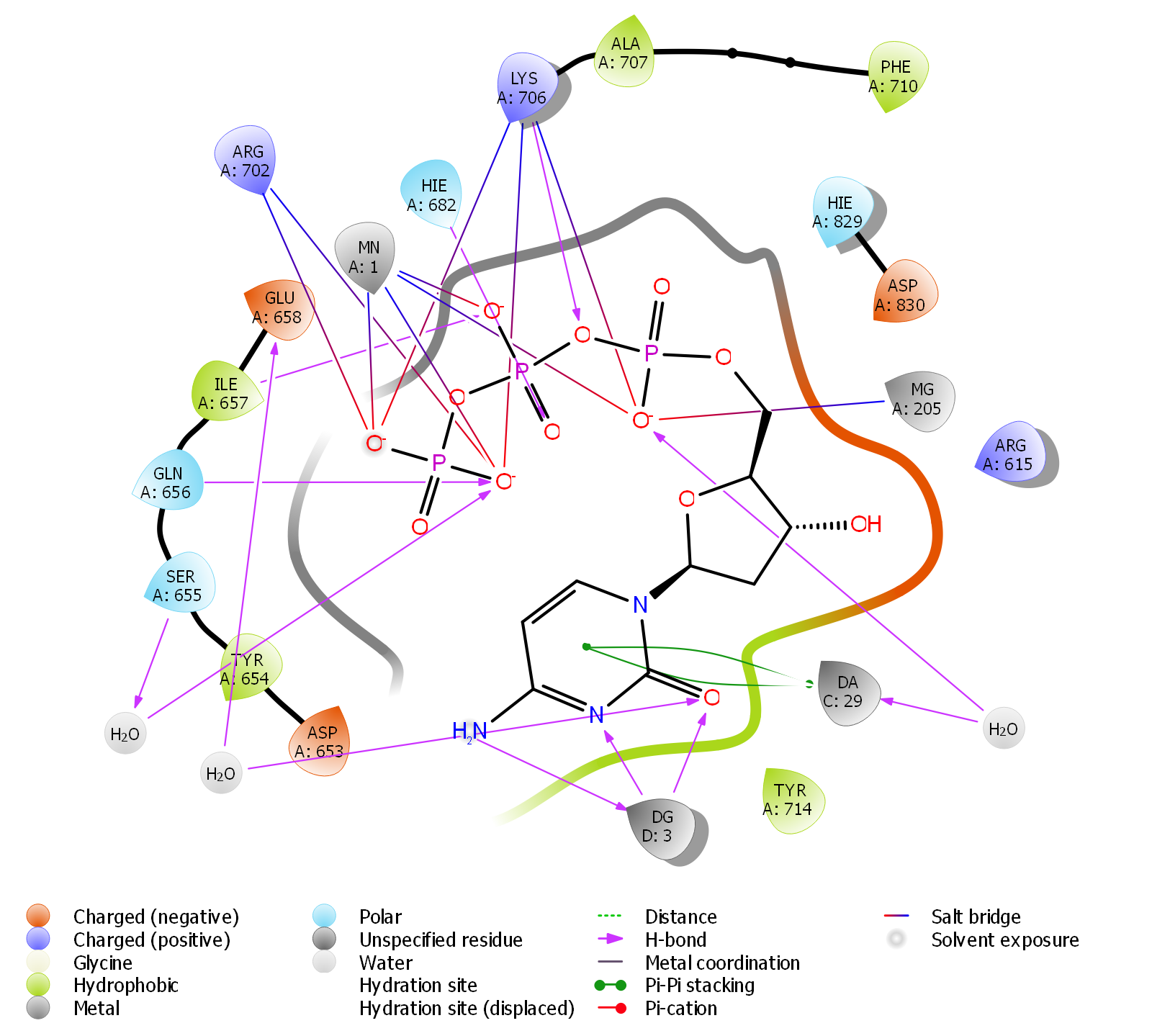

Supplement: Supplementary file 1 [file mmc1.zip › Ca_Mg.png]

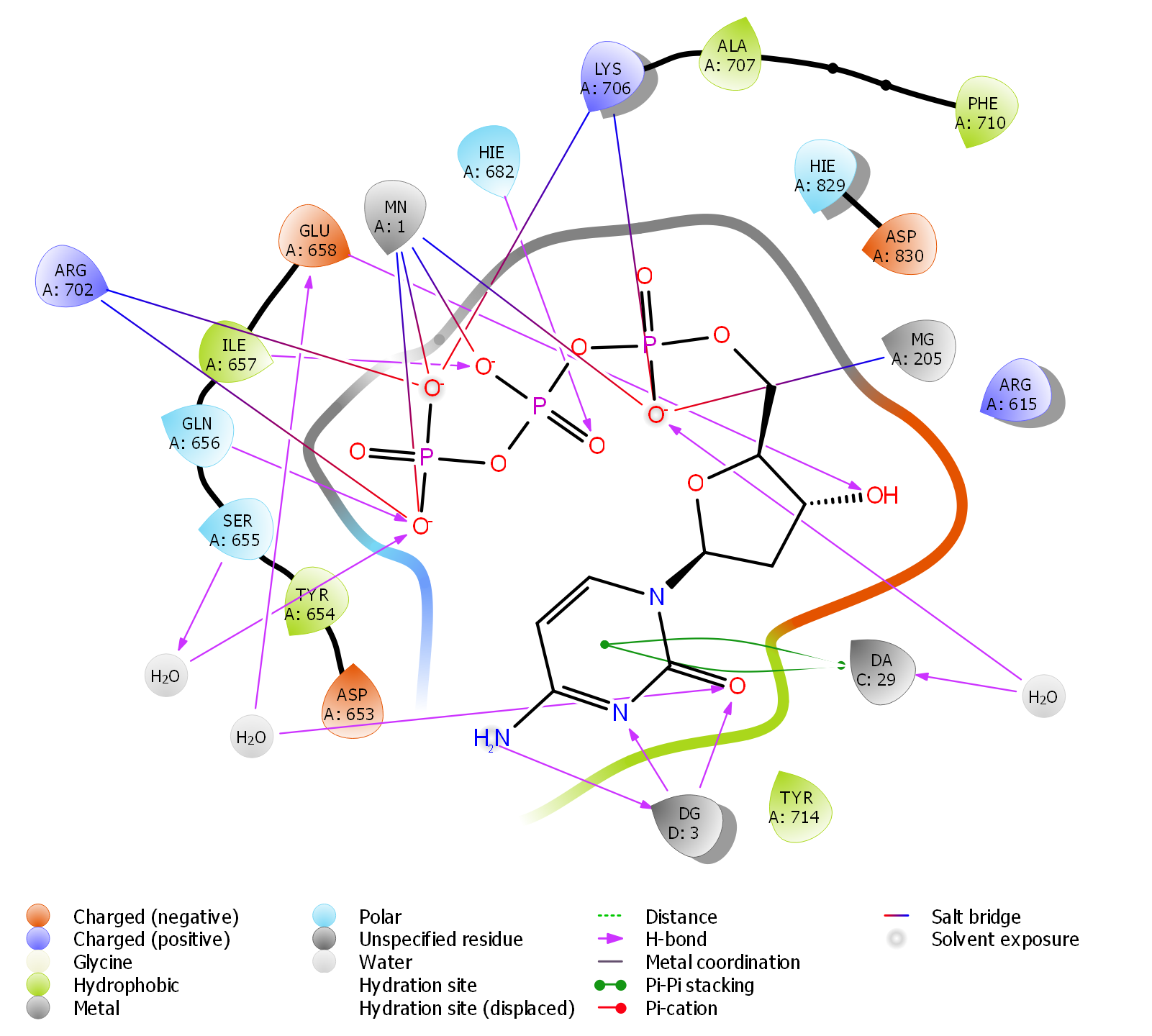

Supplement: Supplementary file 1 [file mmc1.zip › Cd_Mg.png]

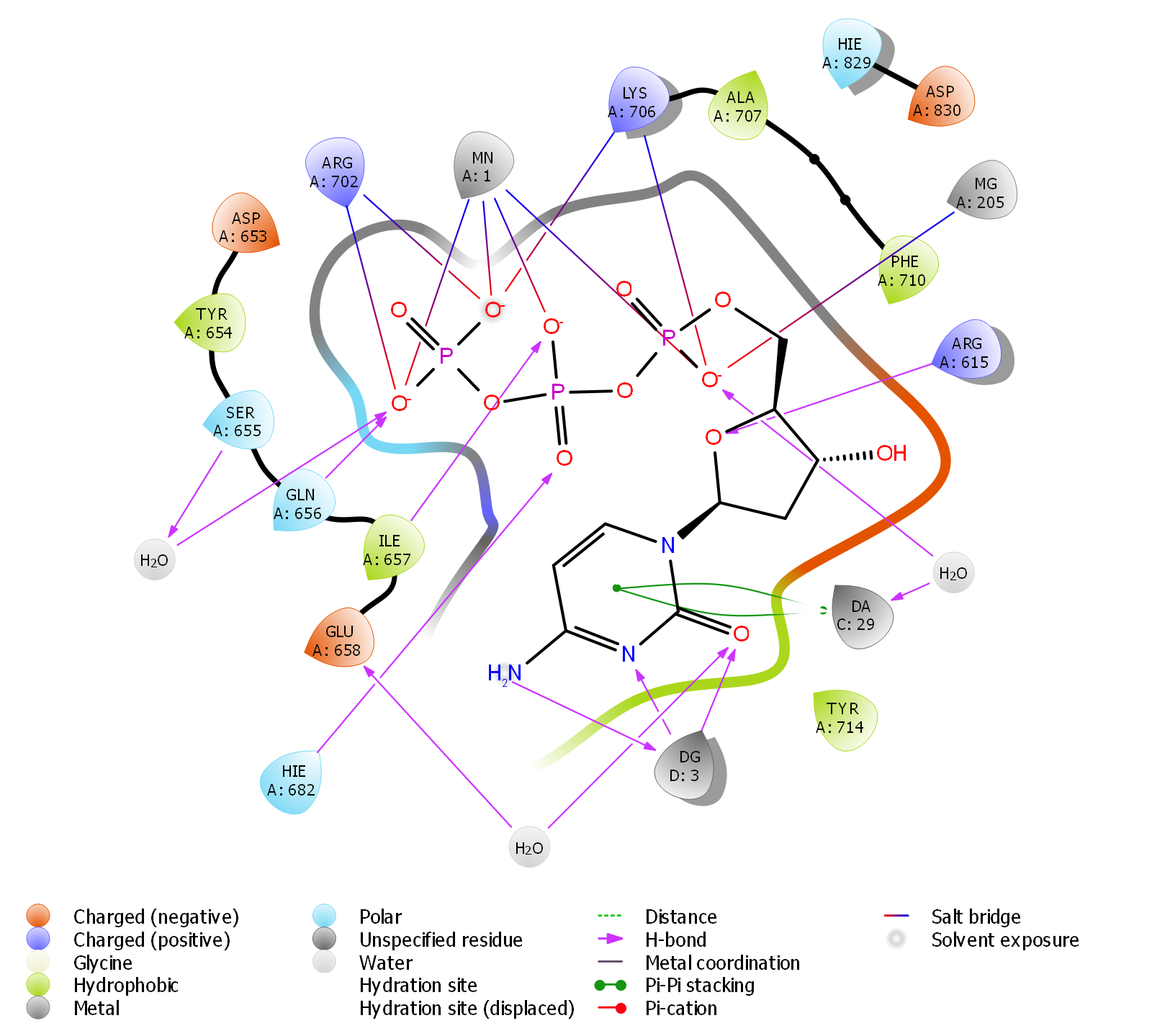

Supplement: Supplementary file 1 [file mmc1.zip › Co_Mg.png]

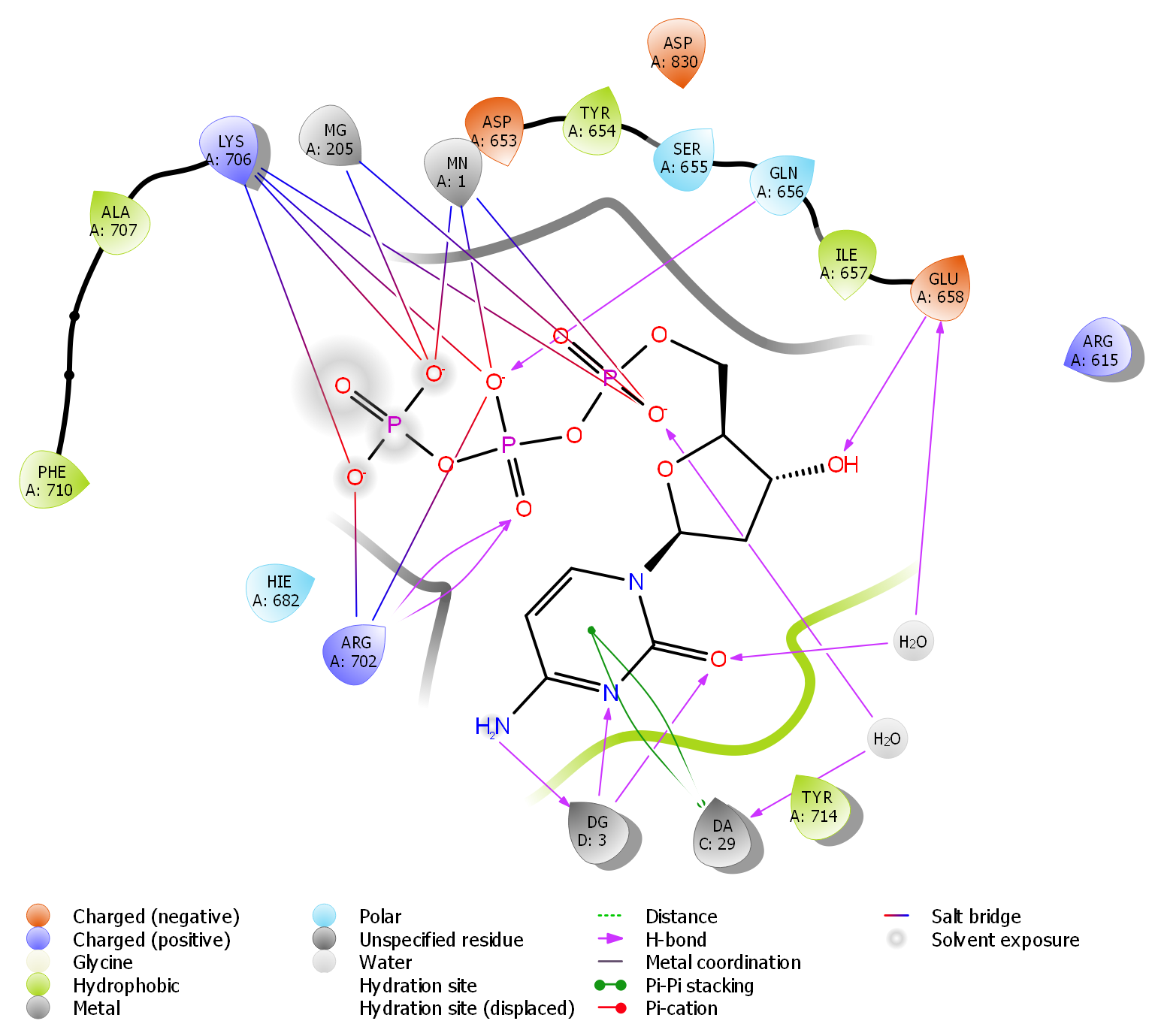

Supplement: Supplementary file 1 [file mmc1.zip › Cu_Mg.png]

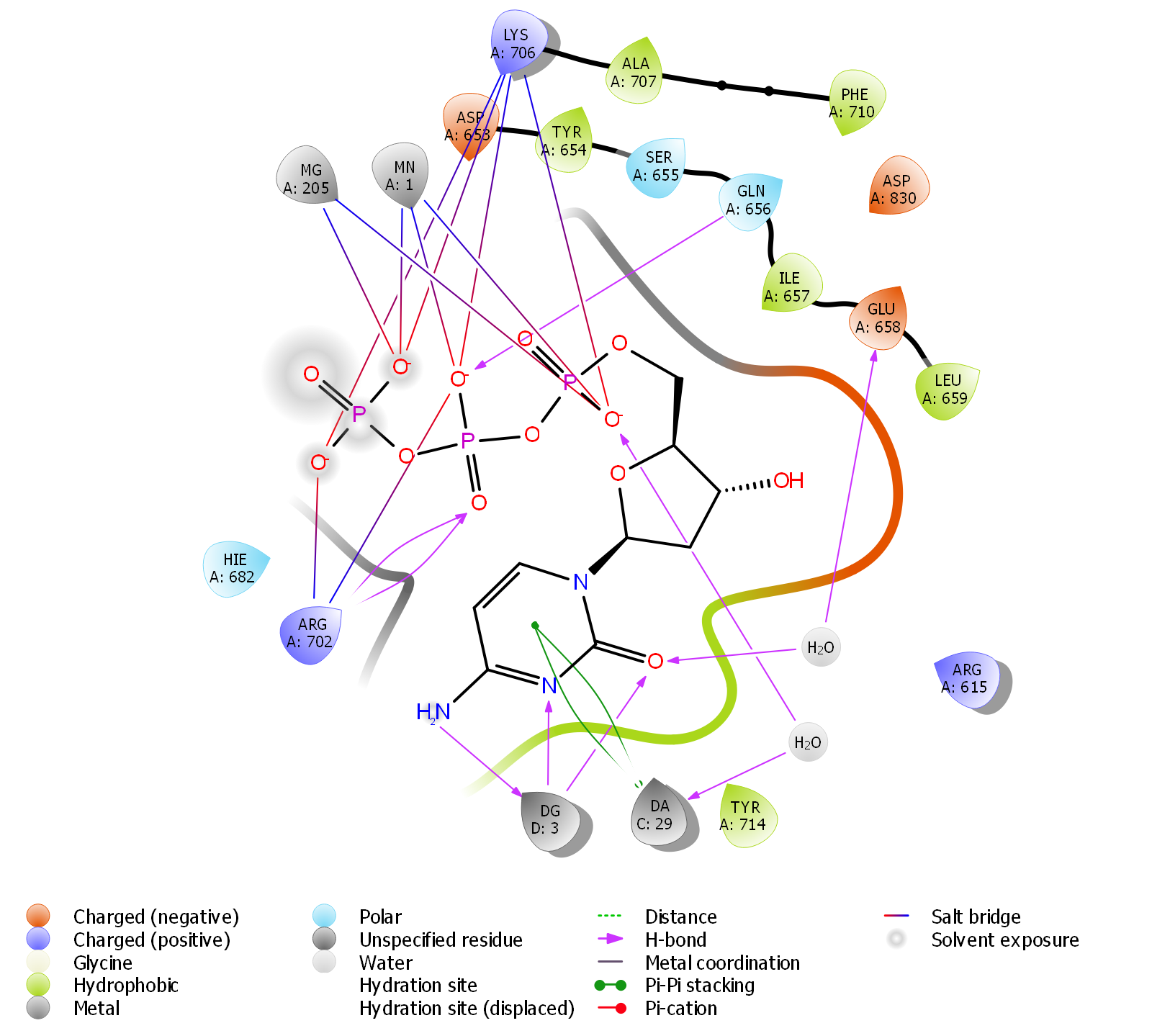

Supplement: Supplementary file 1 [file mmc1.zip › Ca_Ni.png]

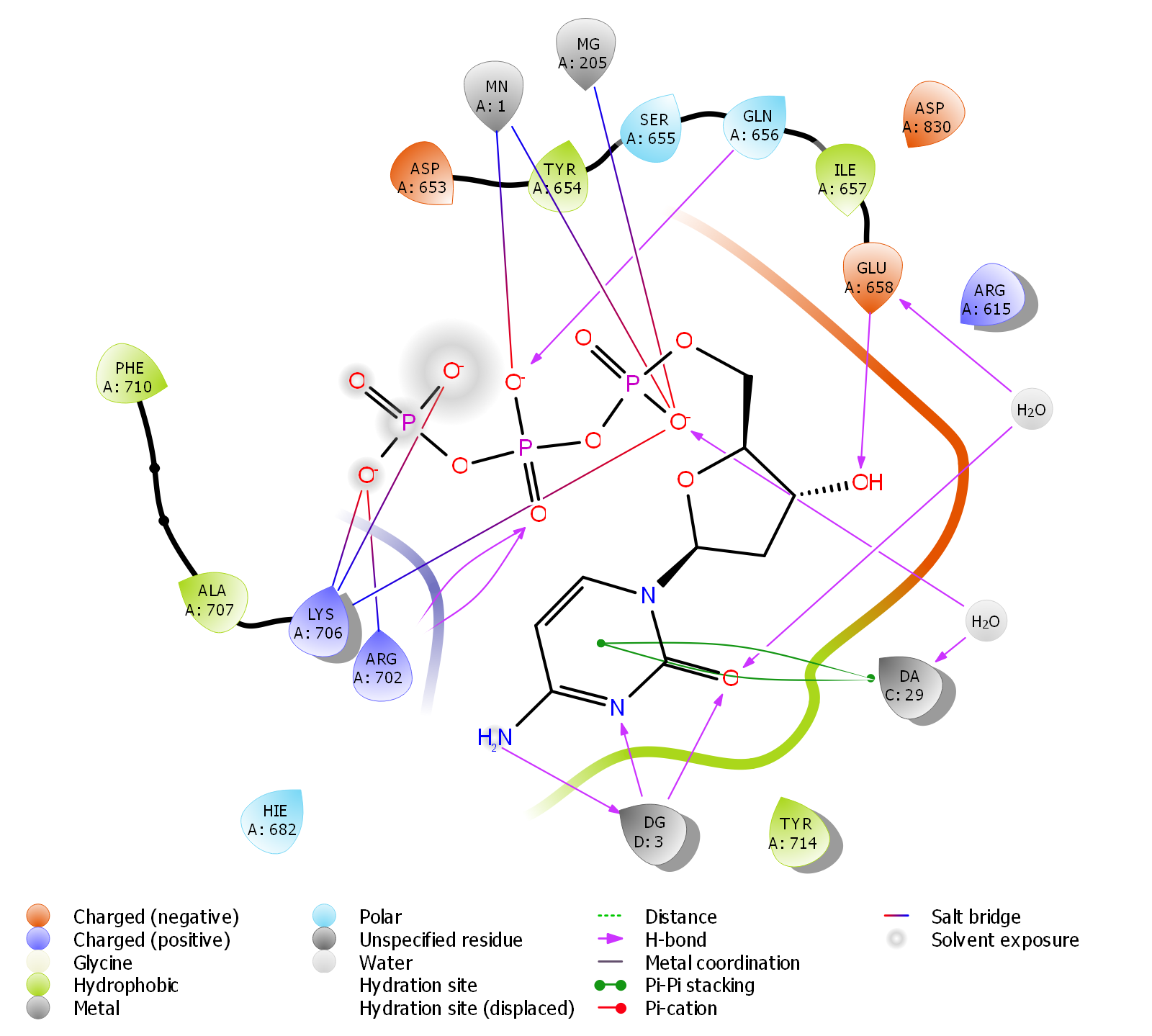

Supplement: Supplementary file 1 [file mmc1.zip › Cd_Ni.png]

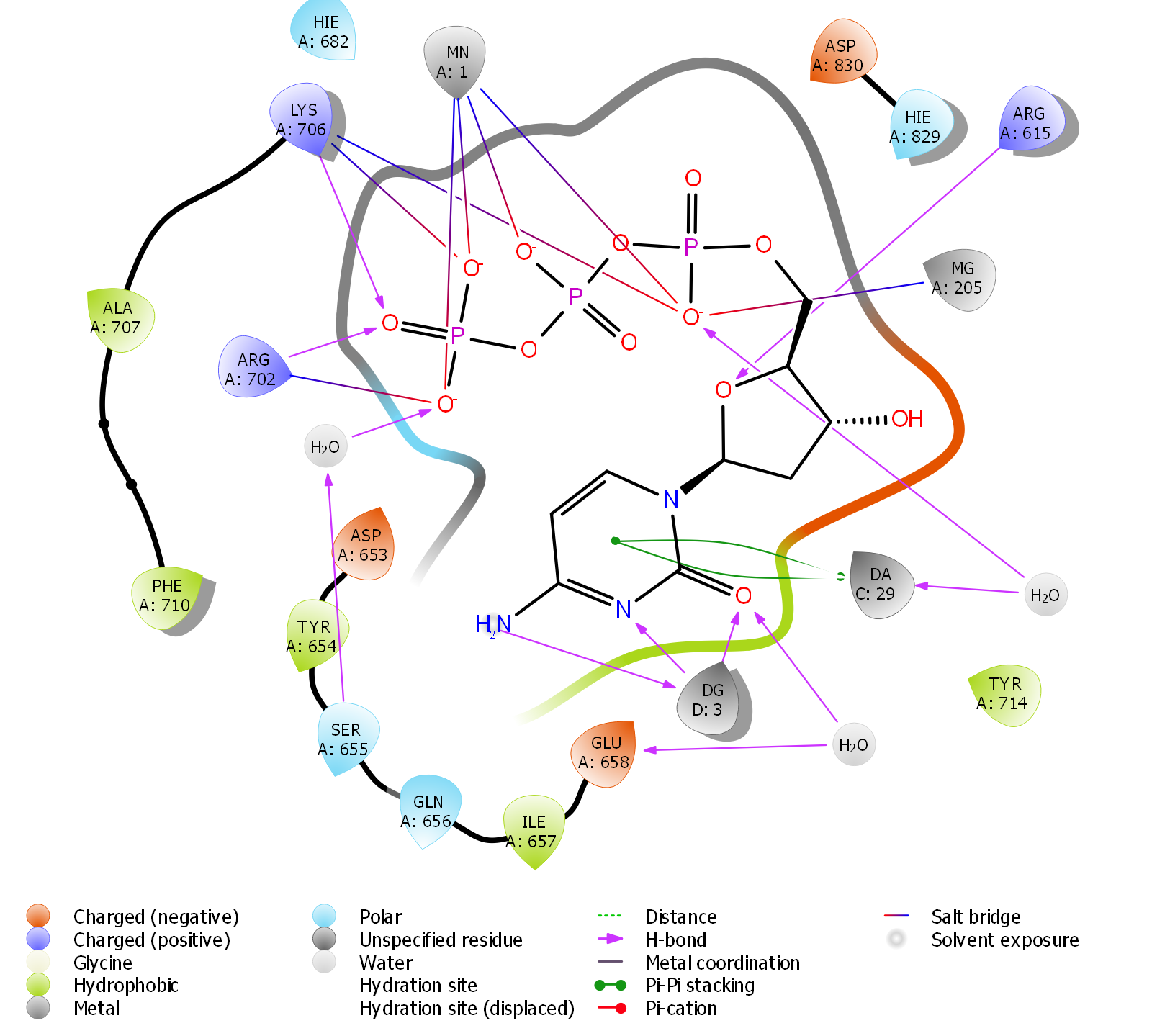

Supplement: Supplementary file 1 [file mmc1.zip › Co_Ni.png]

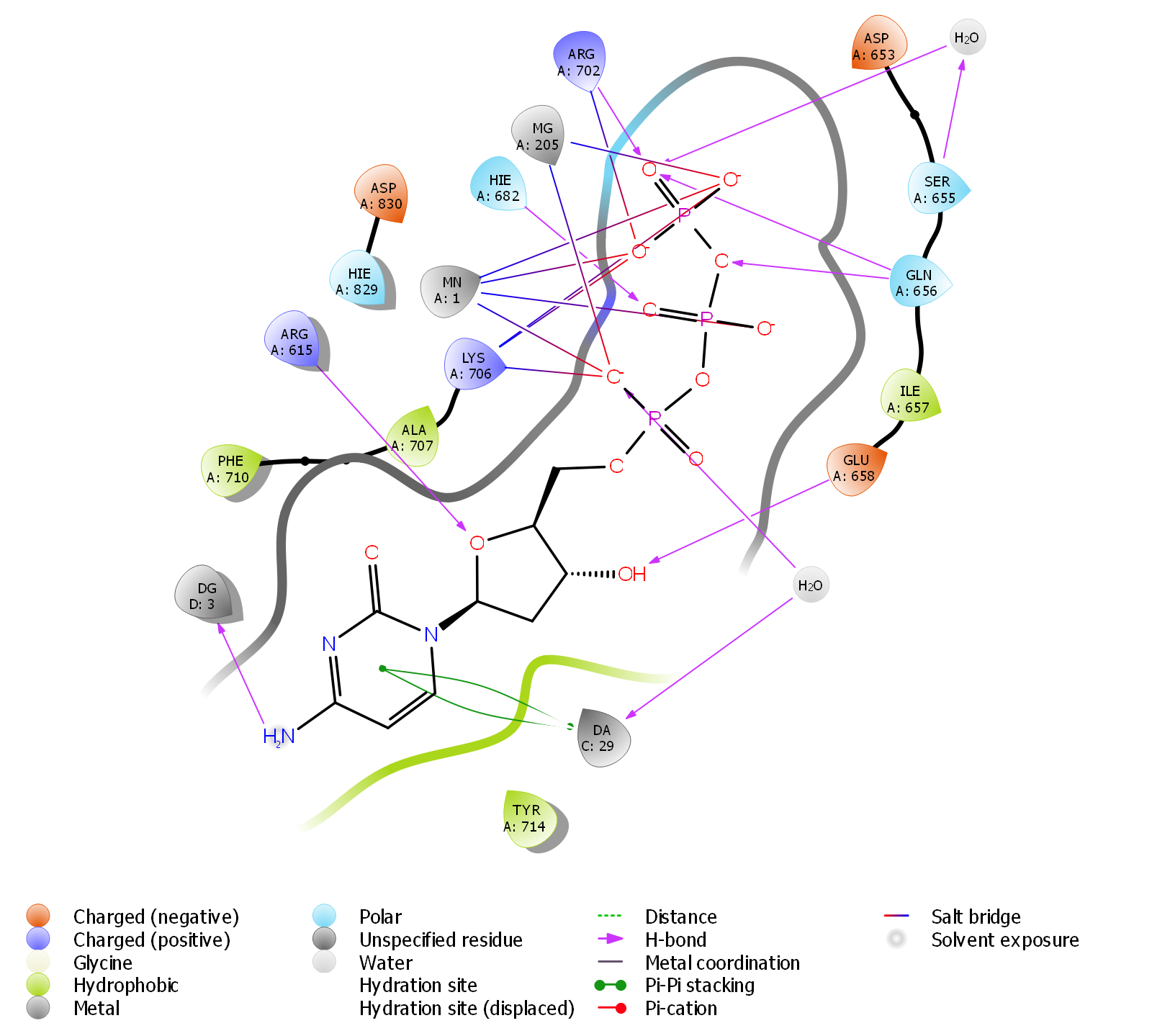

Supplement: Supplementary file 1 [file mmc1.zip › Cu_Ni.png]

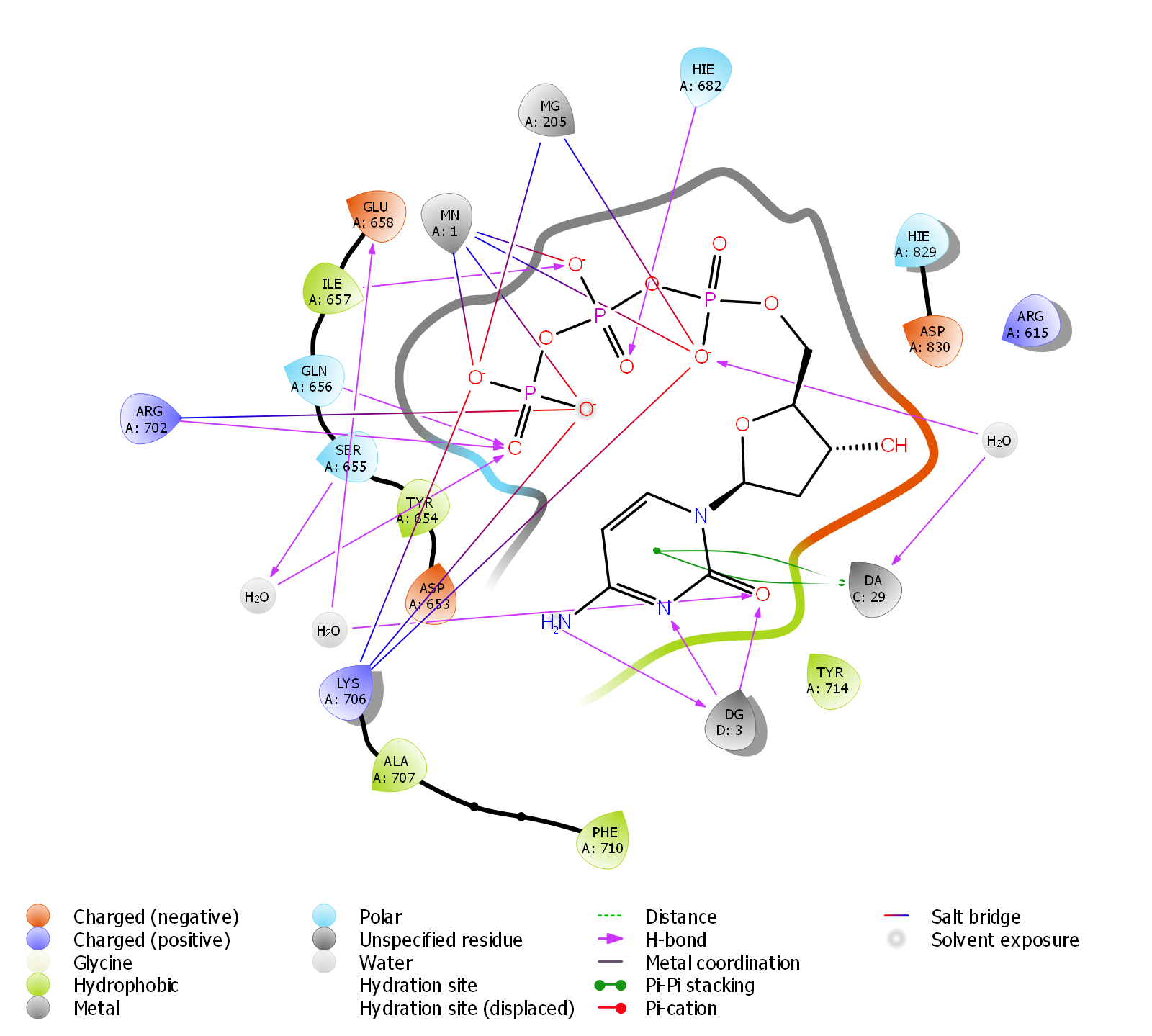

Supplement: Supplementary file 1 [file mmc1.zip › Mn_Ni.png]

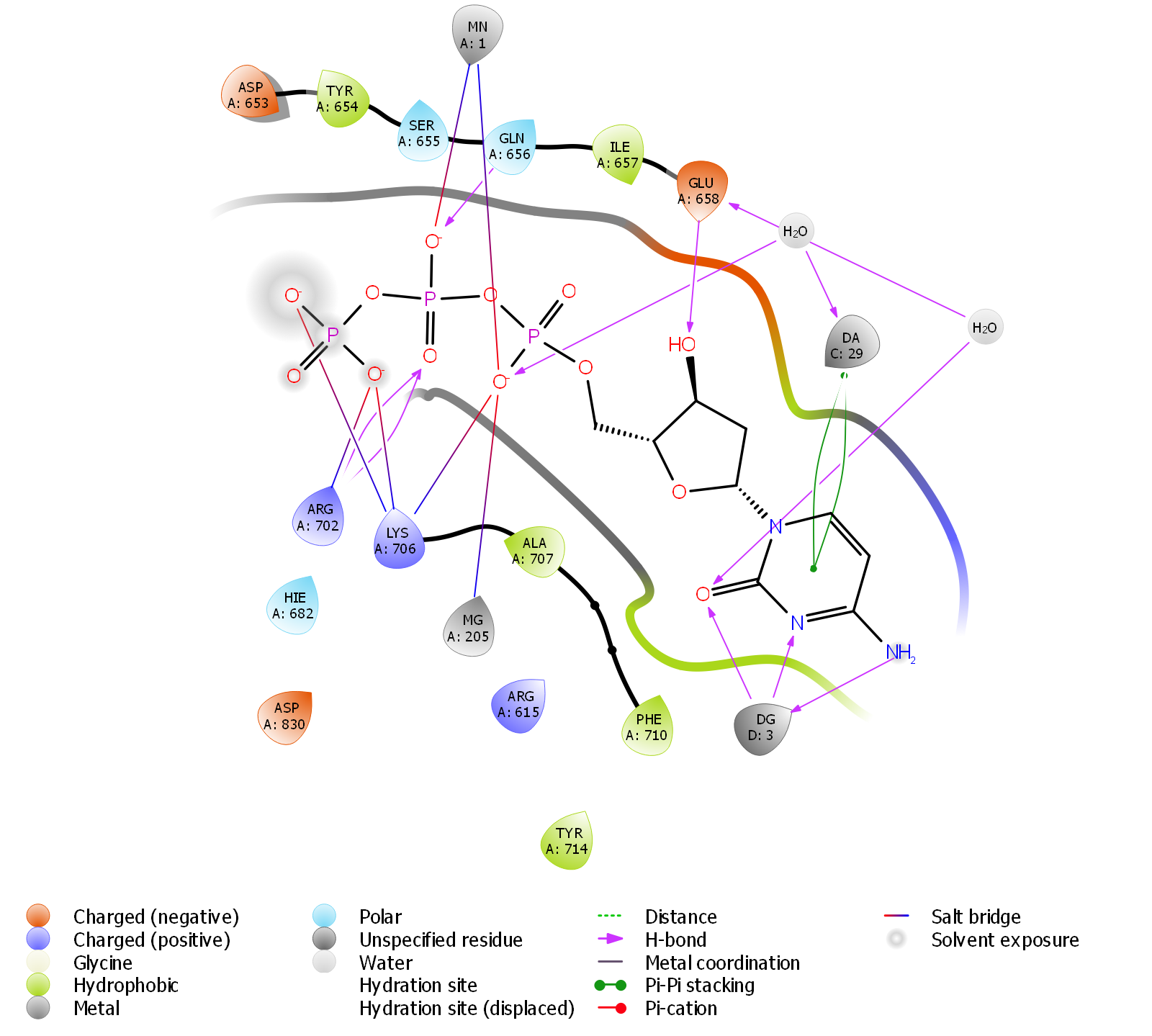

Supplement: Supplementary file 1 [file mmc1.zip › Zn_Ni.png]

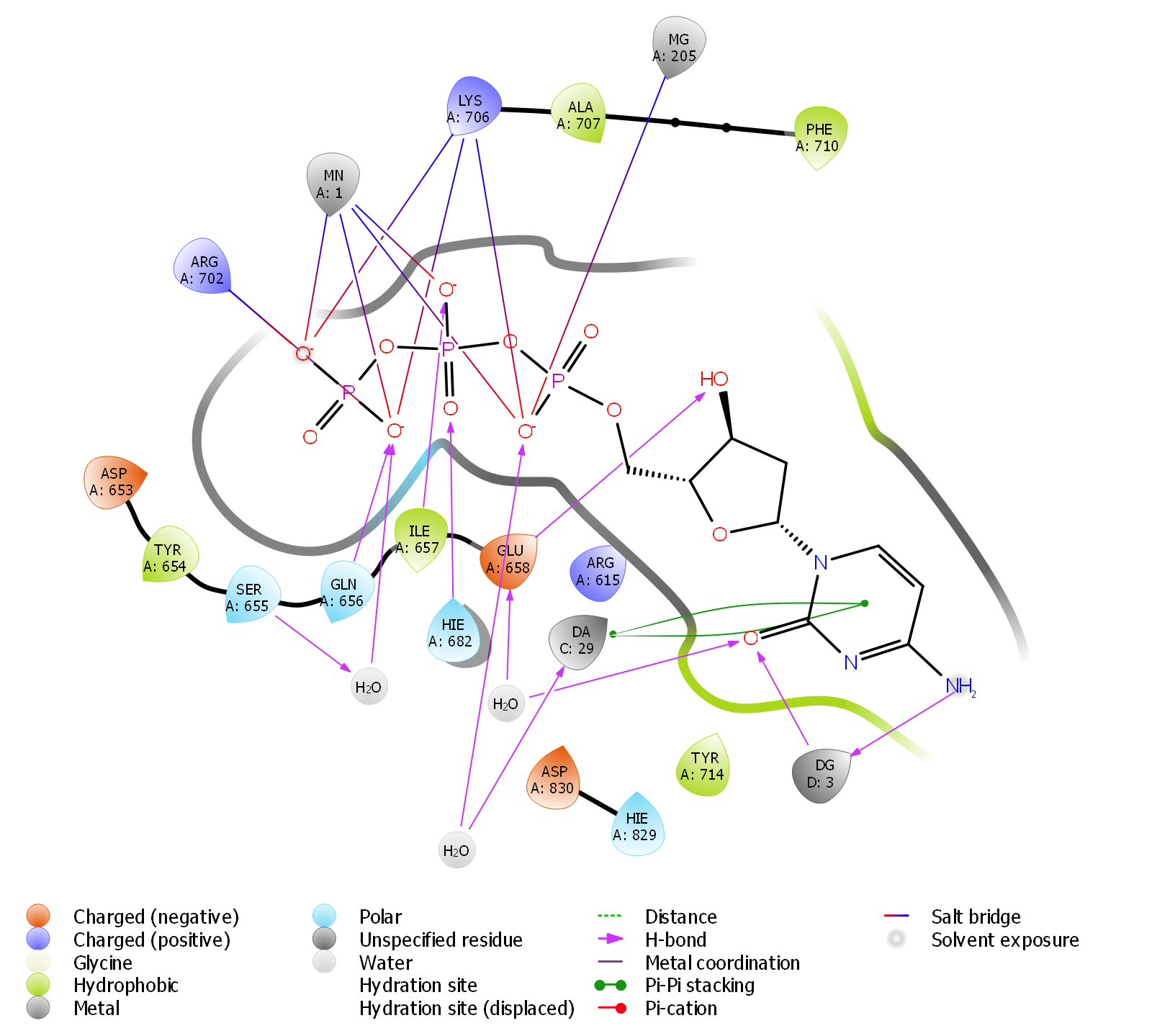

Supplement: Supplementary file 1 [file mmc1.zip › Ca_Zn.png]

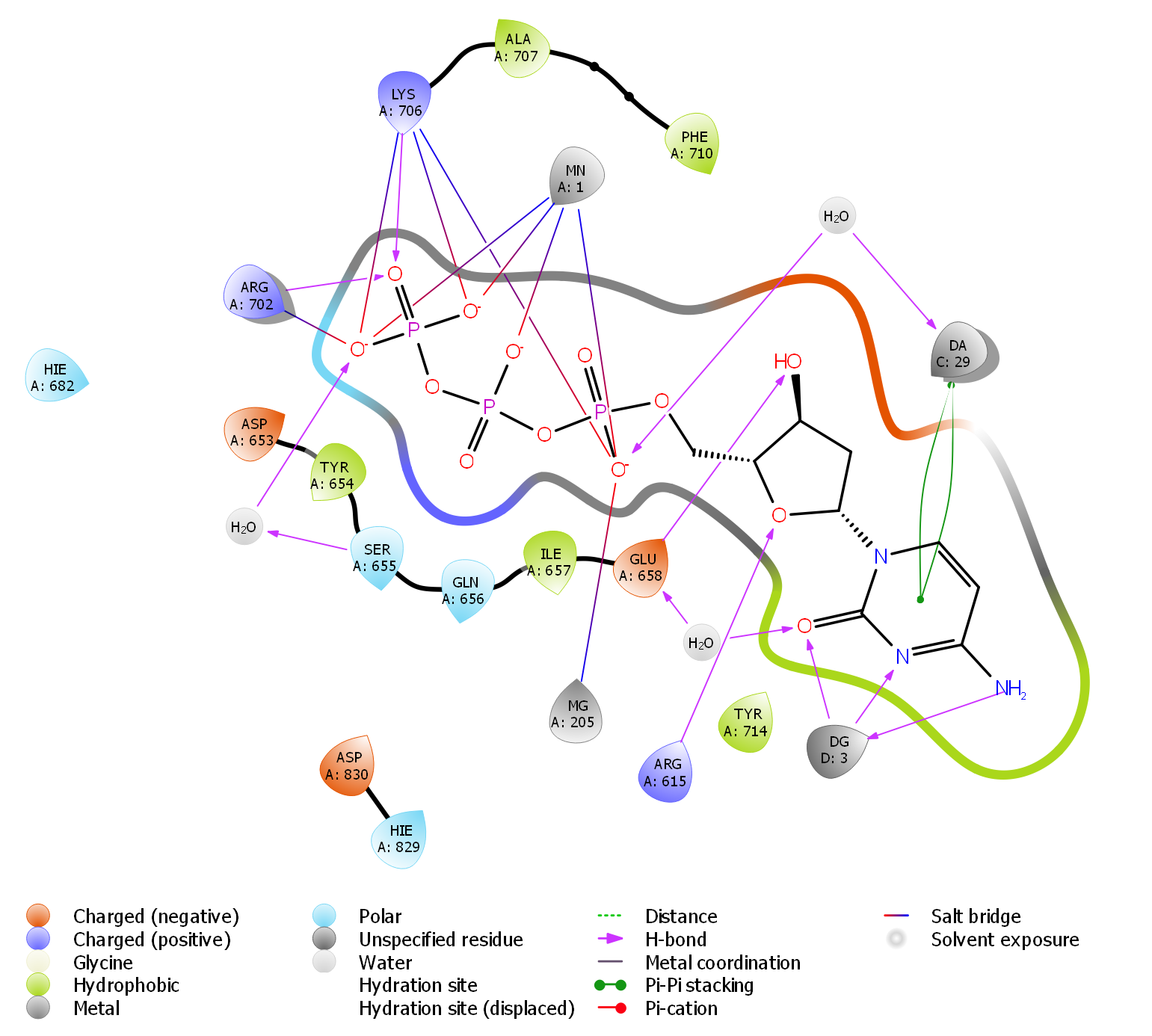

Supplement: Supplementary file 1 [file mmc1.zip › Cd_Zn.png]

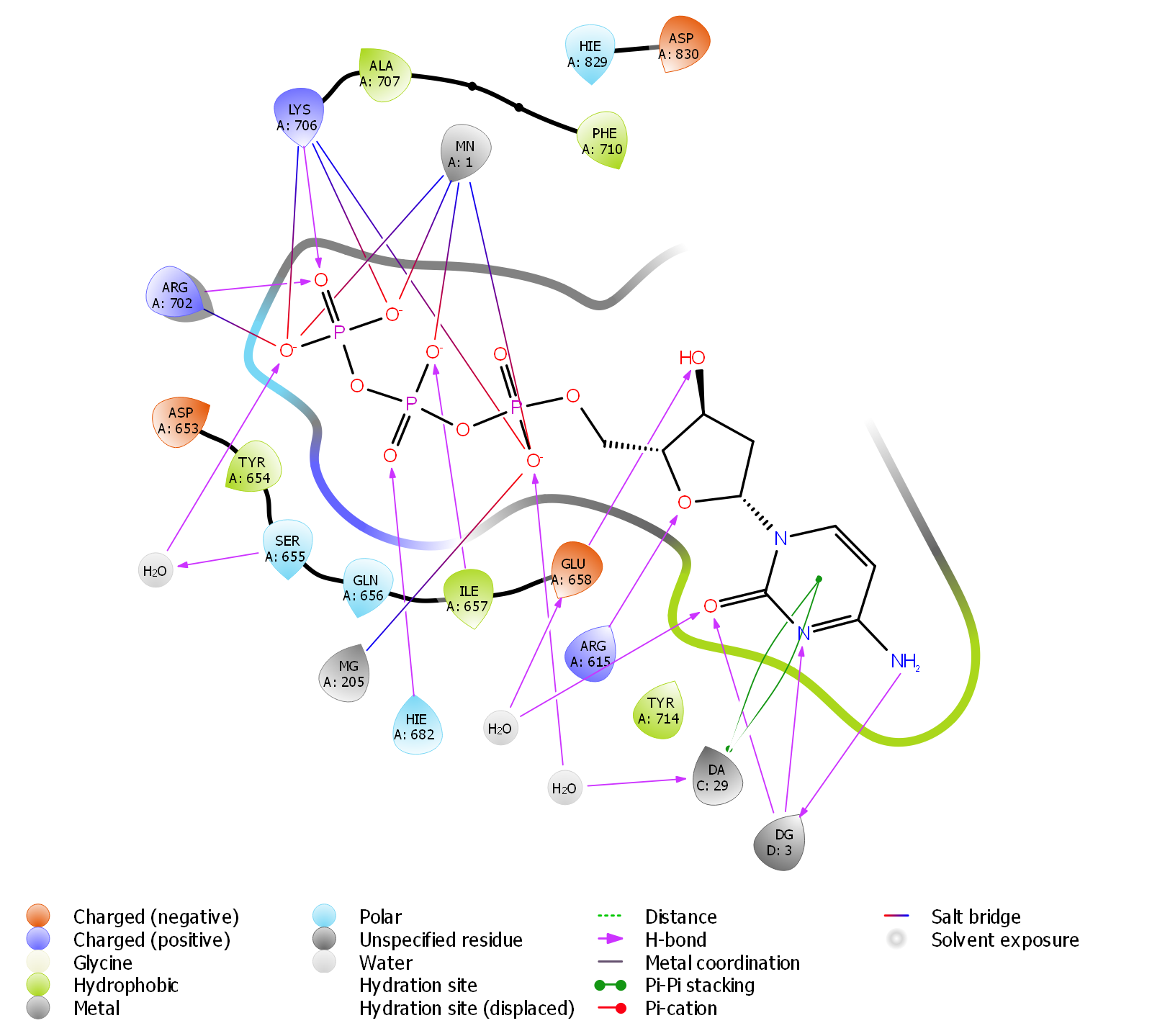

Supplement: Supplementary file 1 [file mmc1.zip › Co_Zn.png]

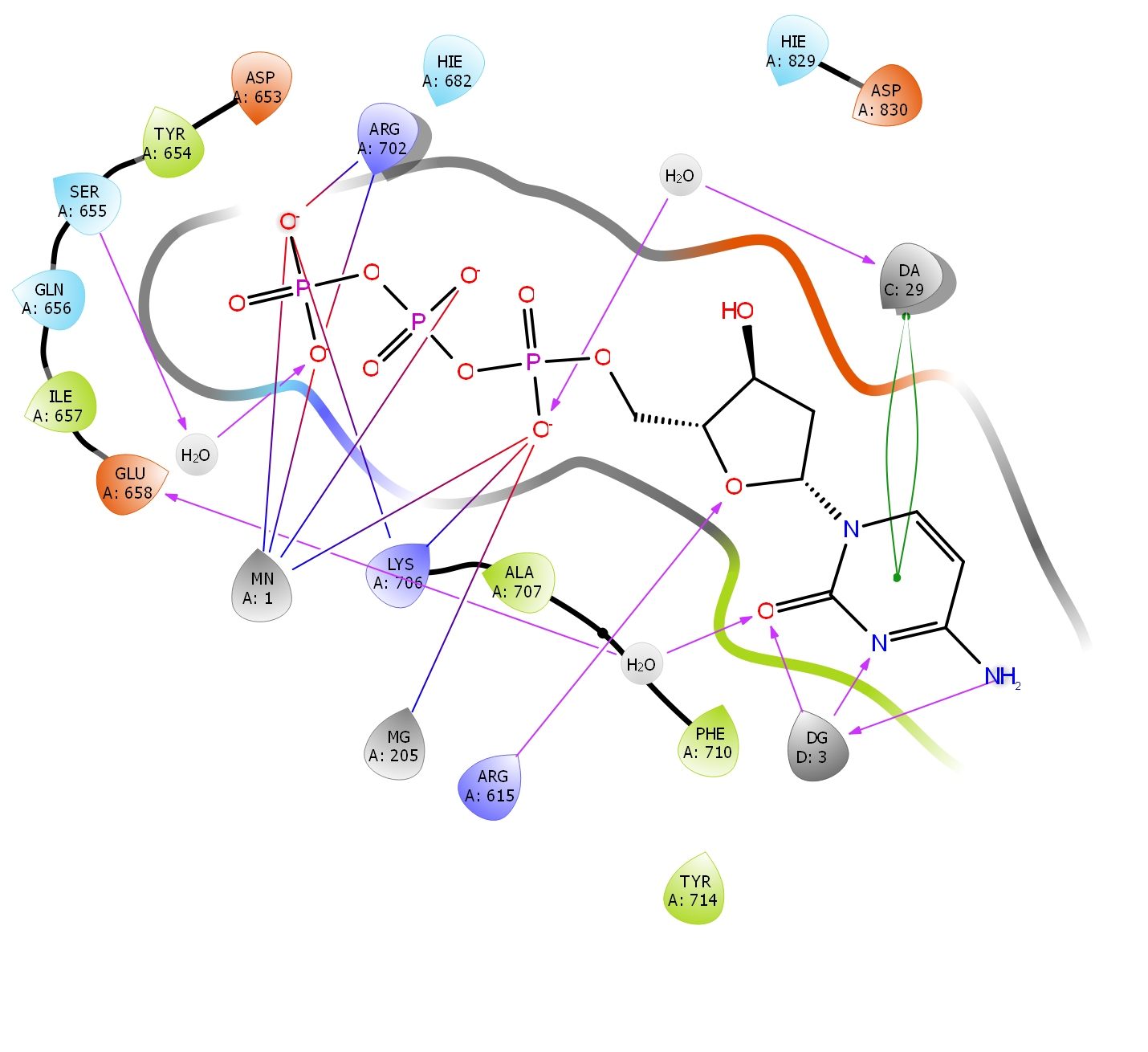

Supplement: Supplementary file 1 [file mmc1.zip › Cu_Zn.png]

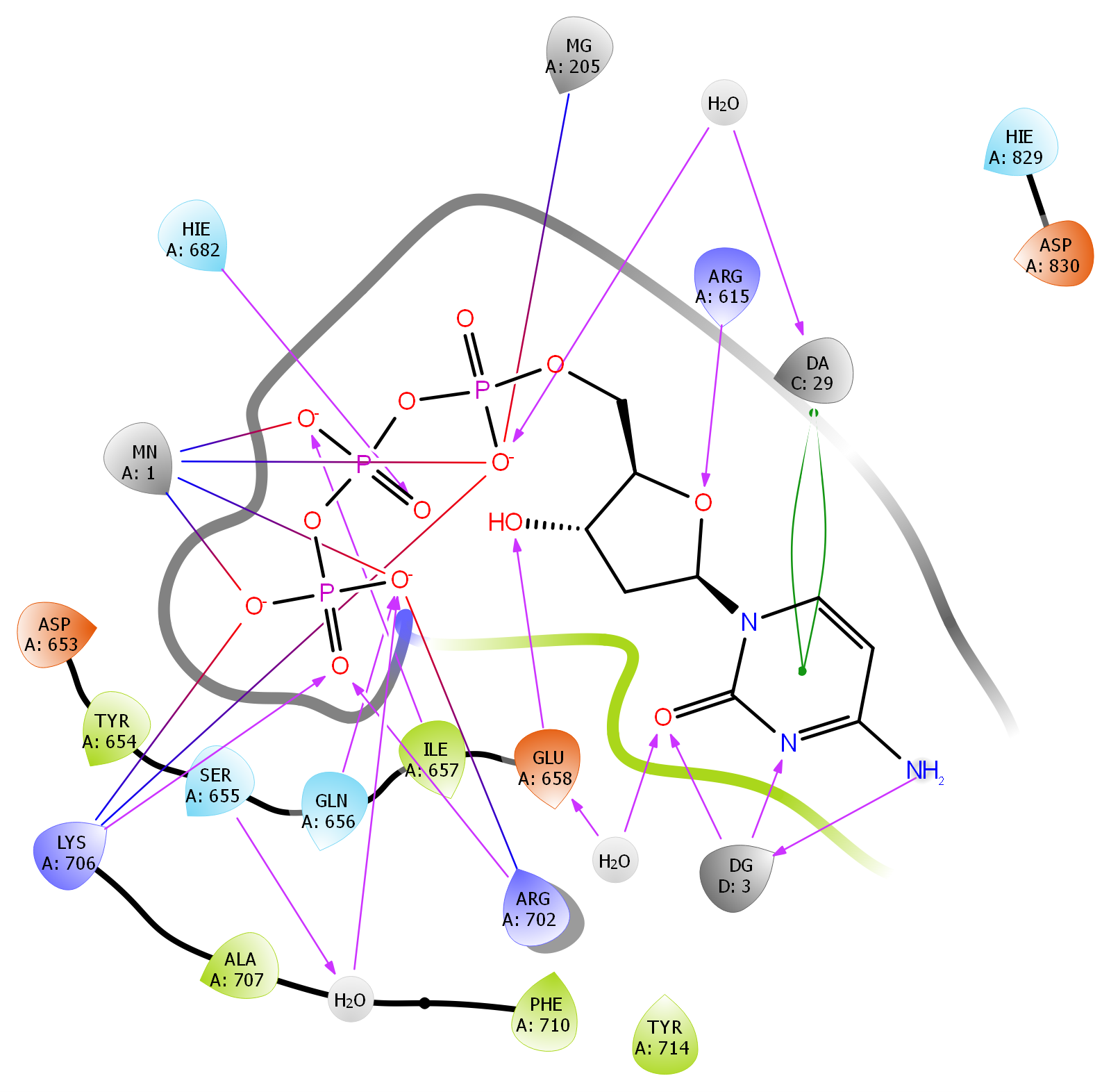

Supplement: Supplementary file 1 [file mmc1.zip › Mn_Zn.png]

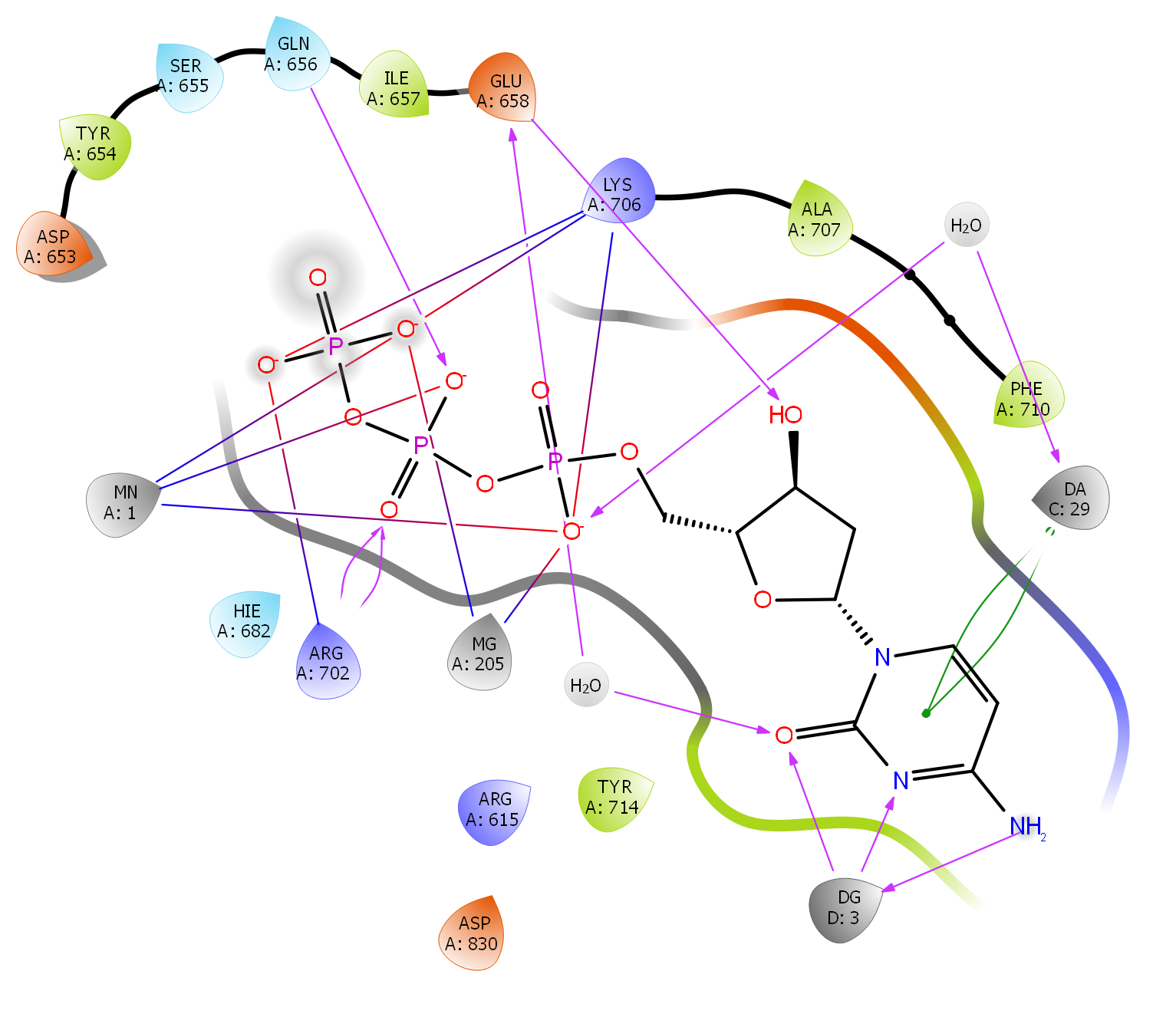

Supplement: Supplementary file 1 [file mmc1.zip › Ni_Zn.png]

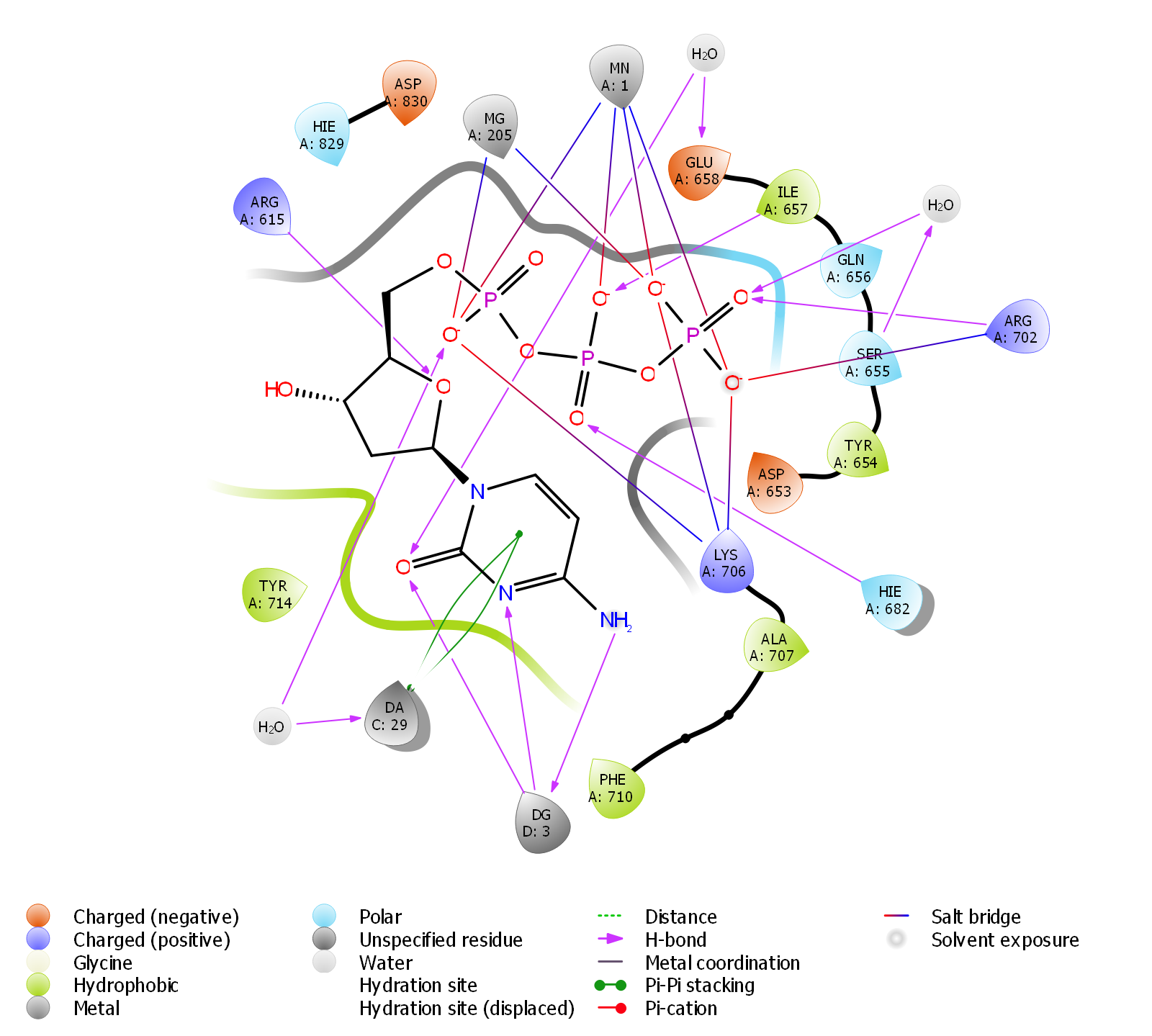

Supplement: Supplementary file 1 [file mmc1.zip › Cd_Cd.png]

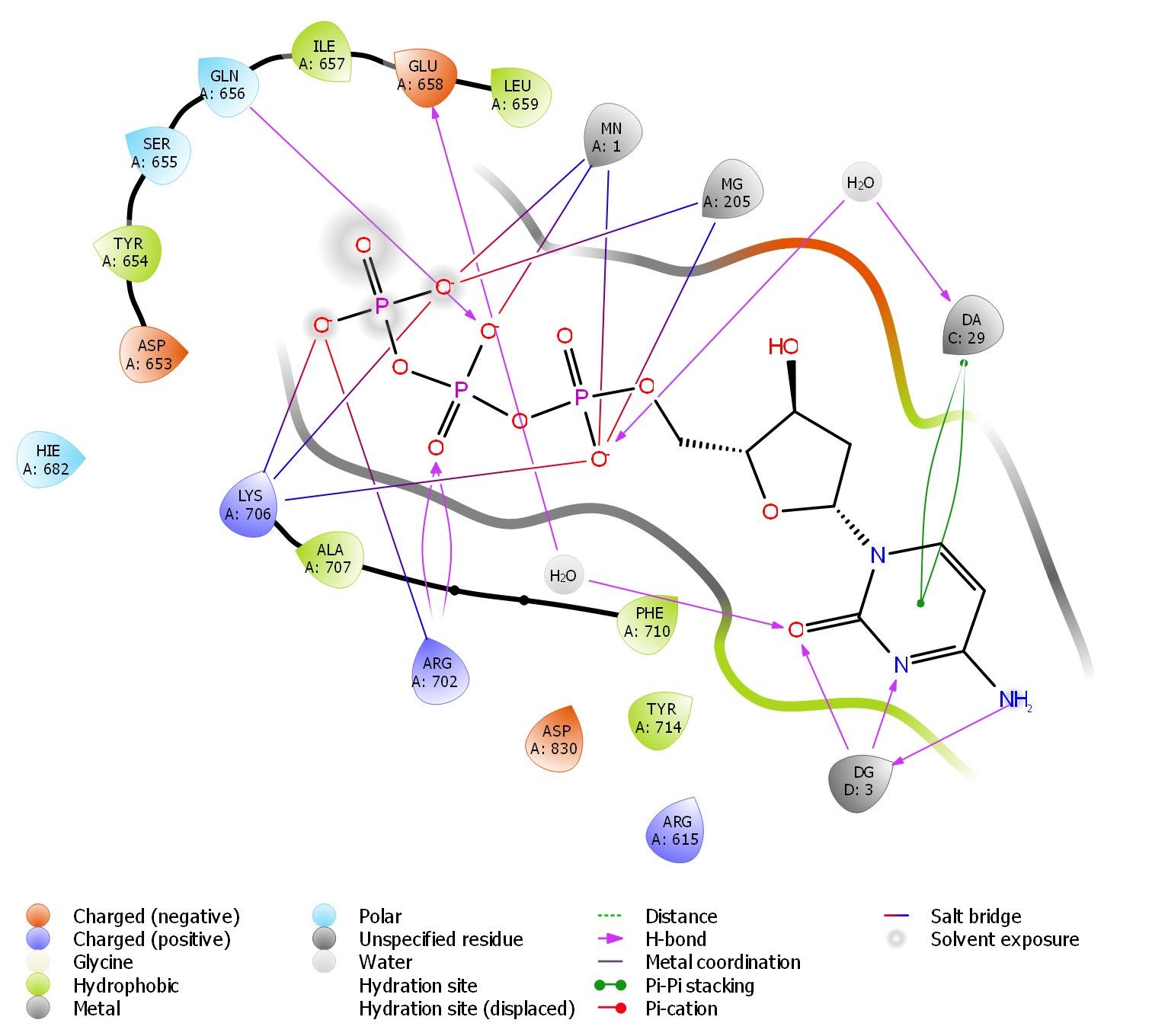

Supplement: Supplementary file 1 [file mmc1.zip › Zn_Mg.png]

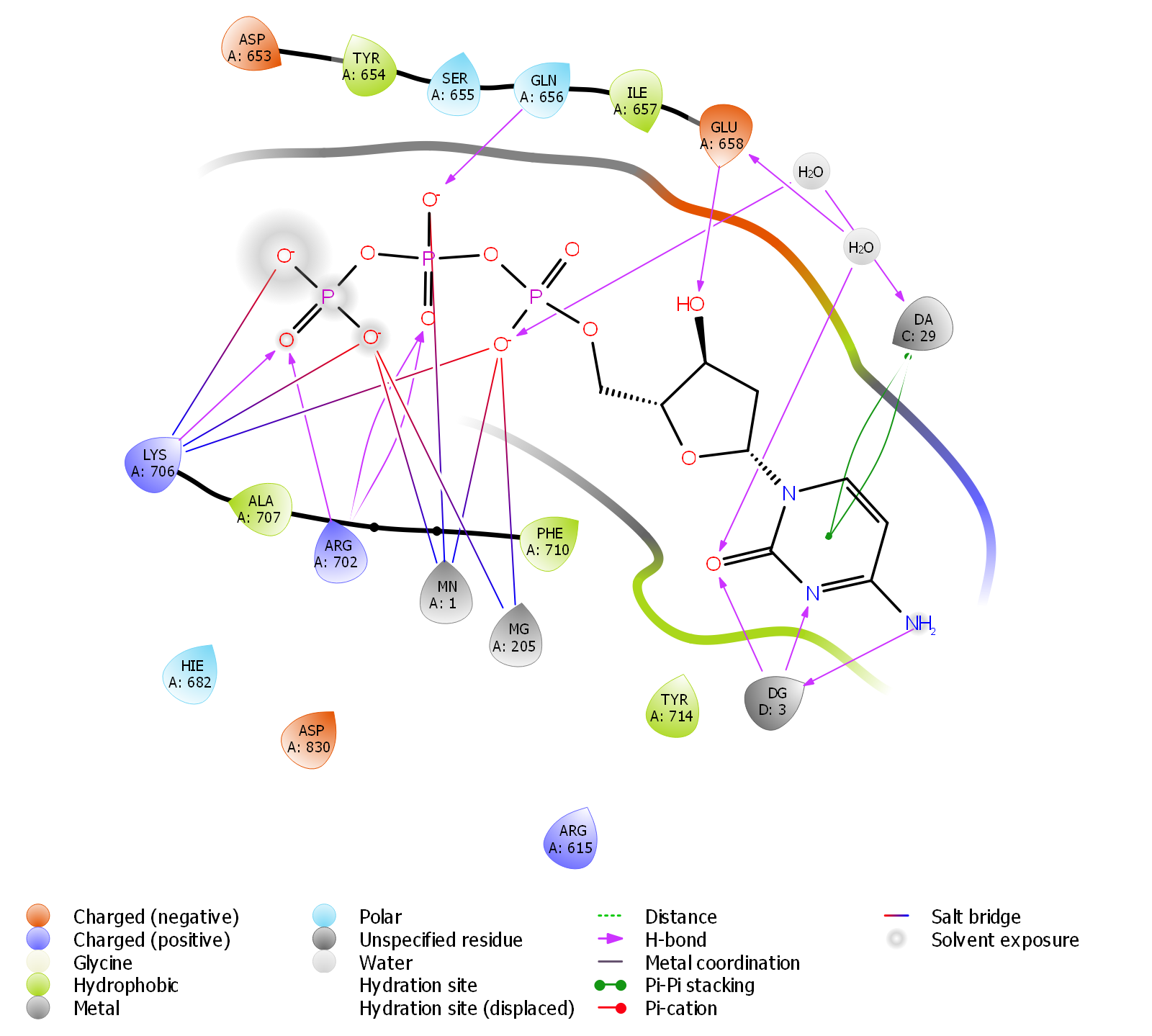

Supplement: Supplementary file 1 [file mmc1.zip › Ni_Mg.png]

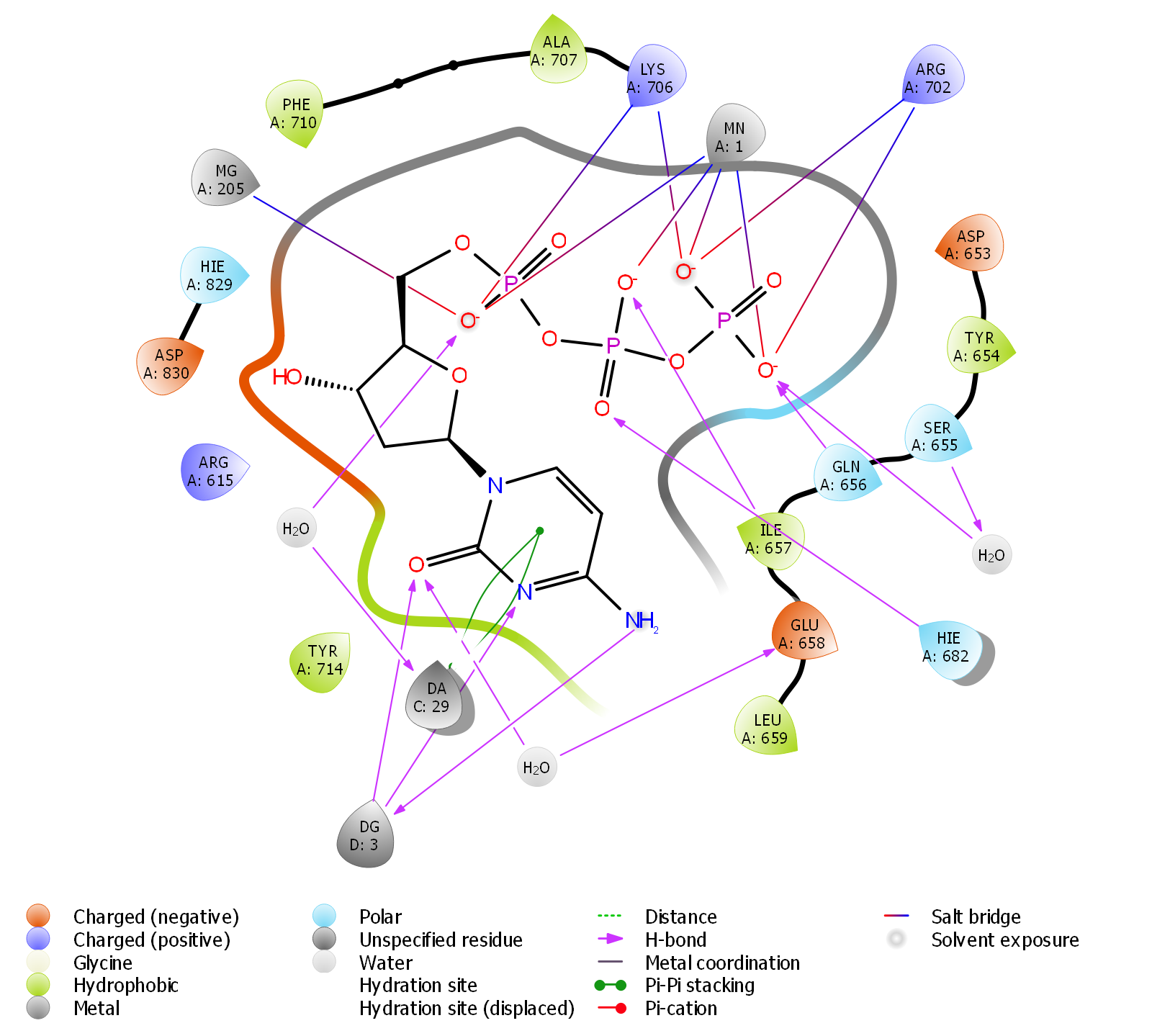

Supplement: Supplementary file 1 [file mmc1.zip › Mn_Mn.png]

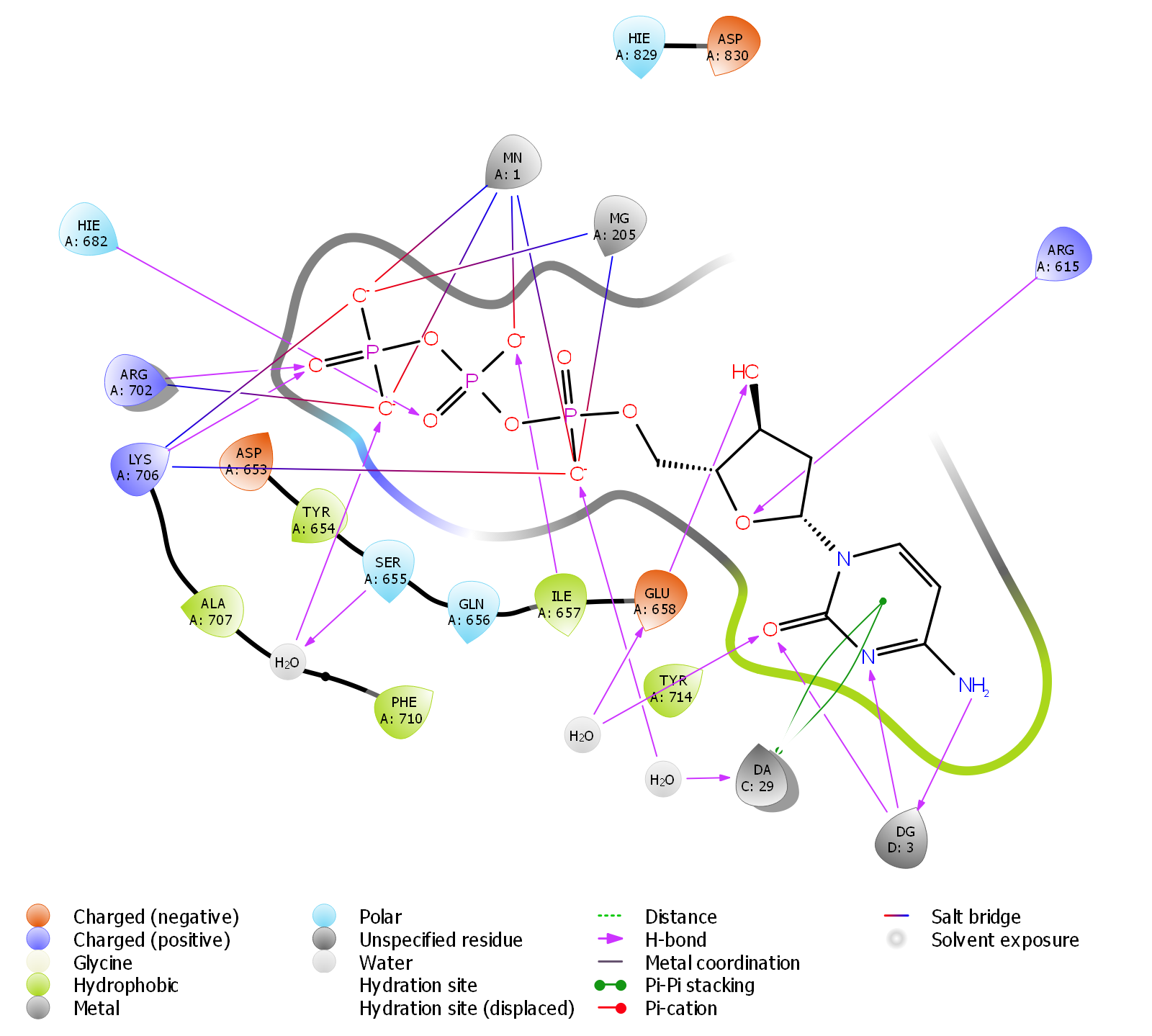

Supplement: Supplementary file 1 [file mmc1.zip › Mn_Mg.png]

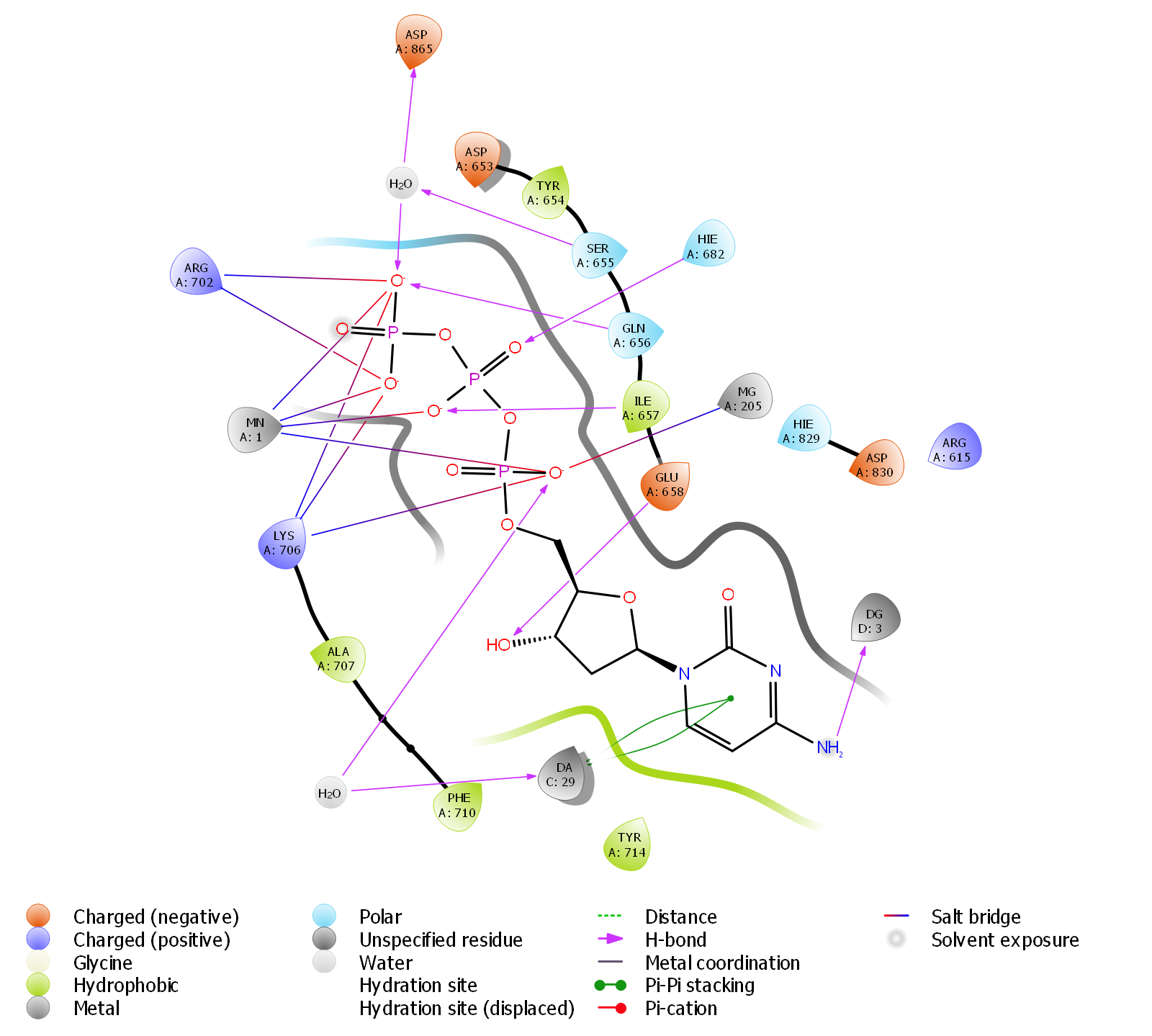

Supplement: Supplementary file 1 [file mmc1.zip › Mg_Zn.png]

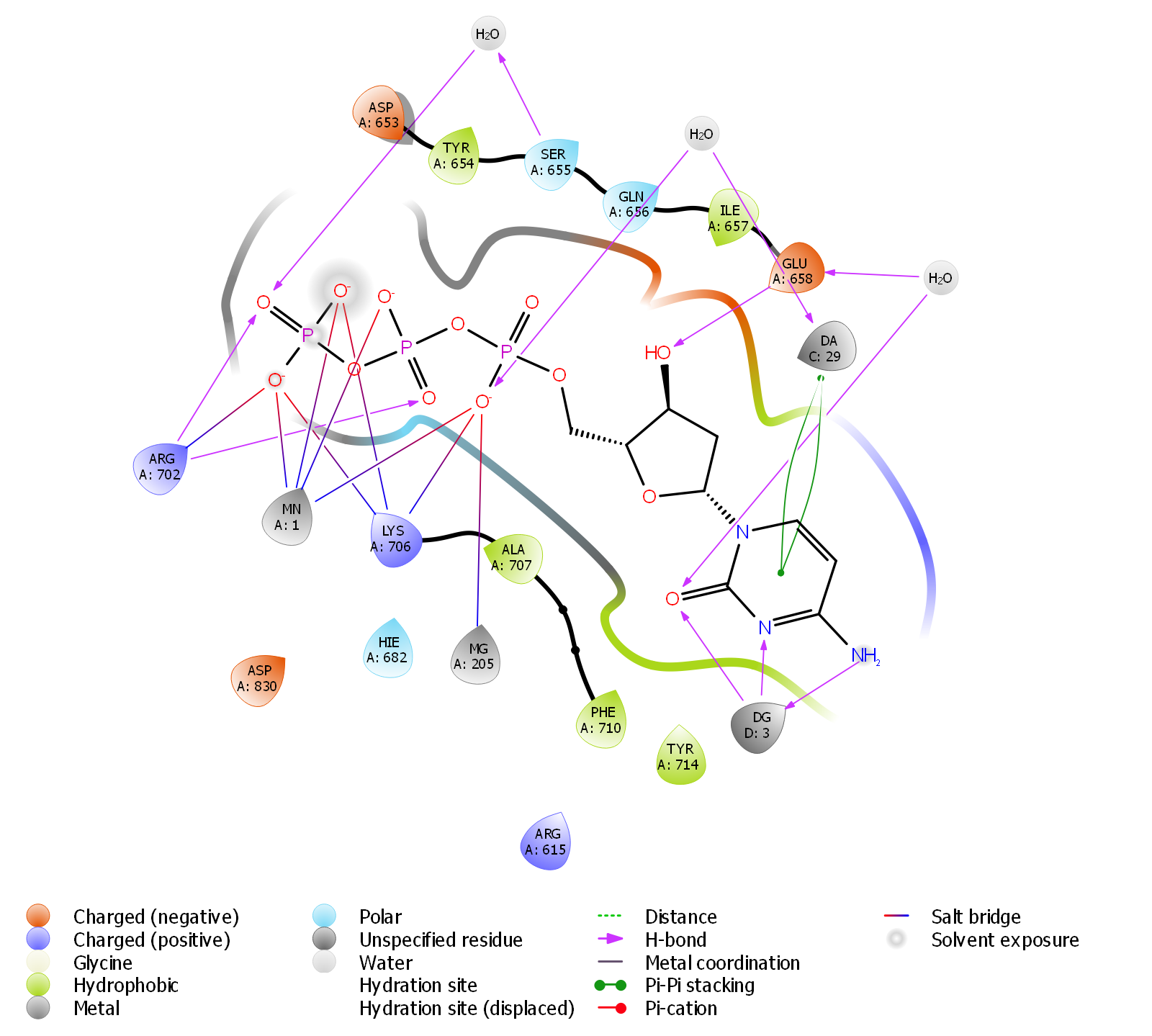

Supplement: Supplementary file 1 [file mmc1.zip › Mg_Ni.png]

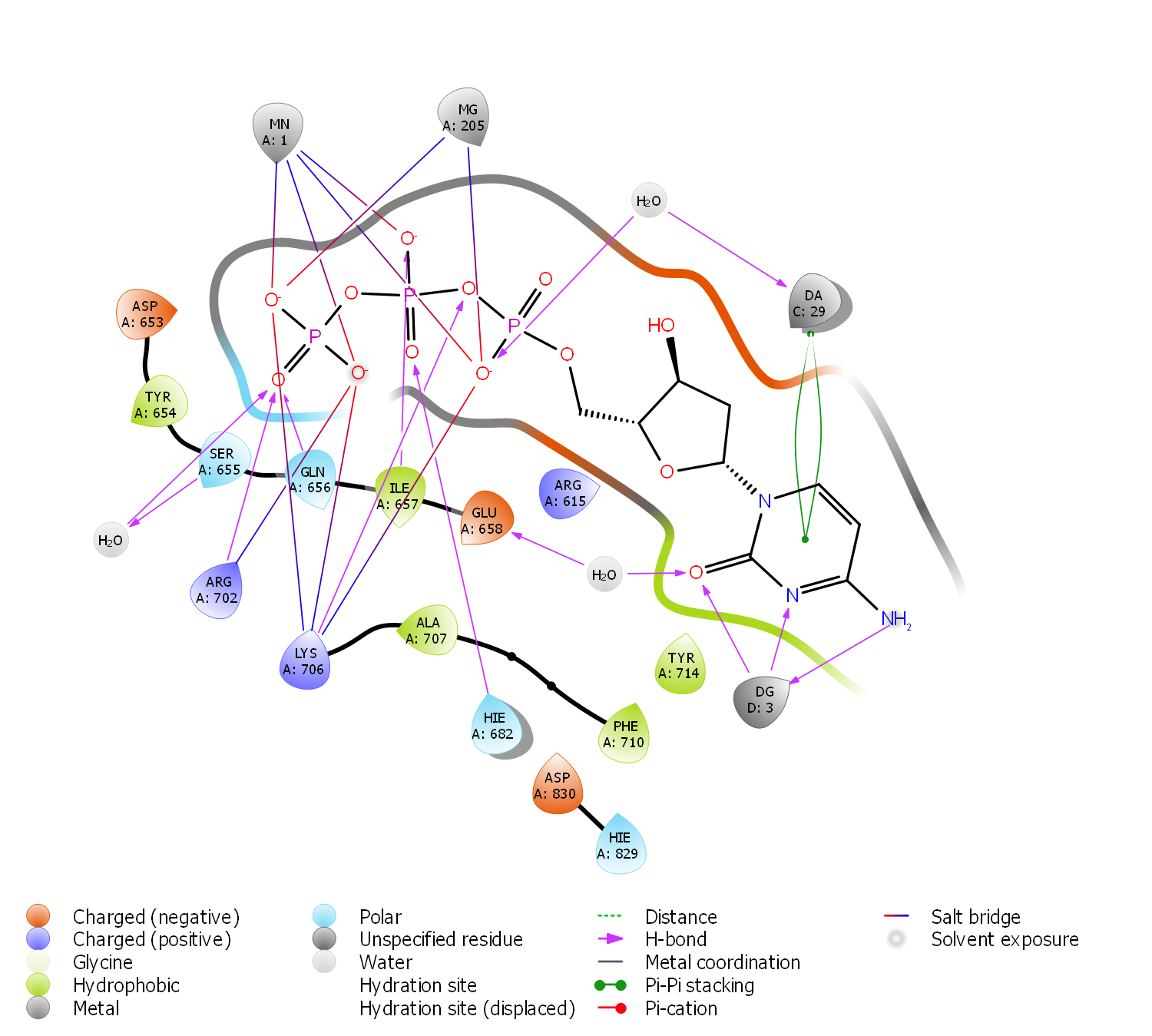

Supplement: Supplementary file 1 [file mmc1.zip › Mg_Mn (1).png]

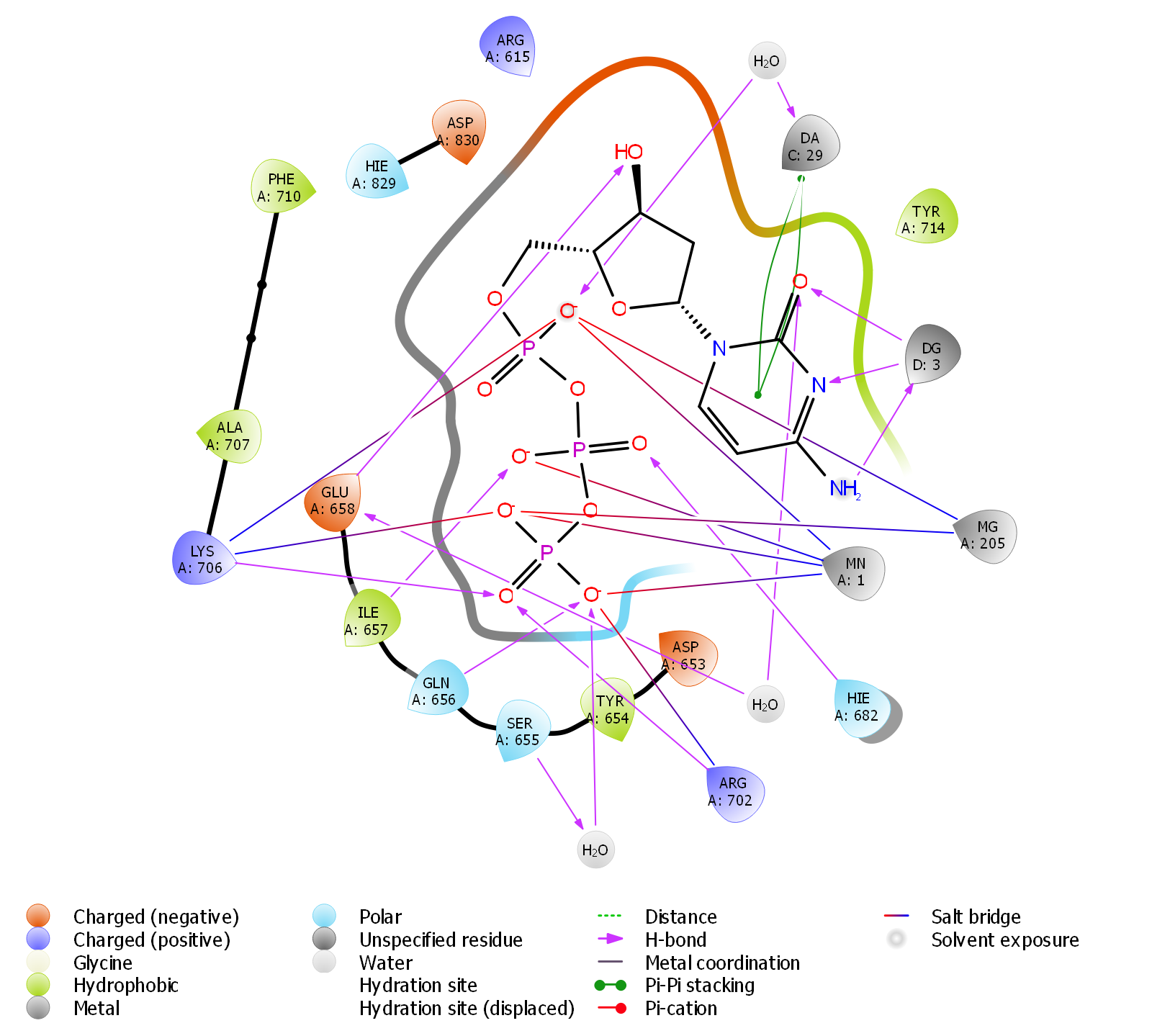

Supplement: Supplementary file 1 [file mmc1.zip › Mg_Mg.png]

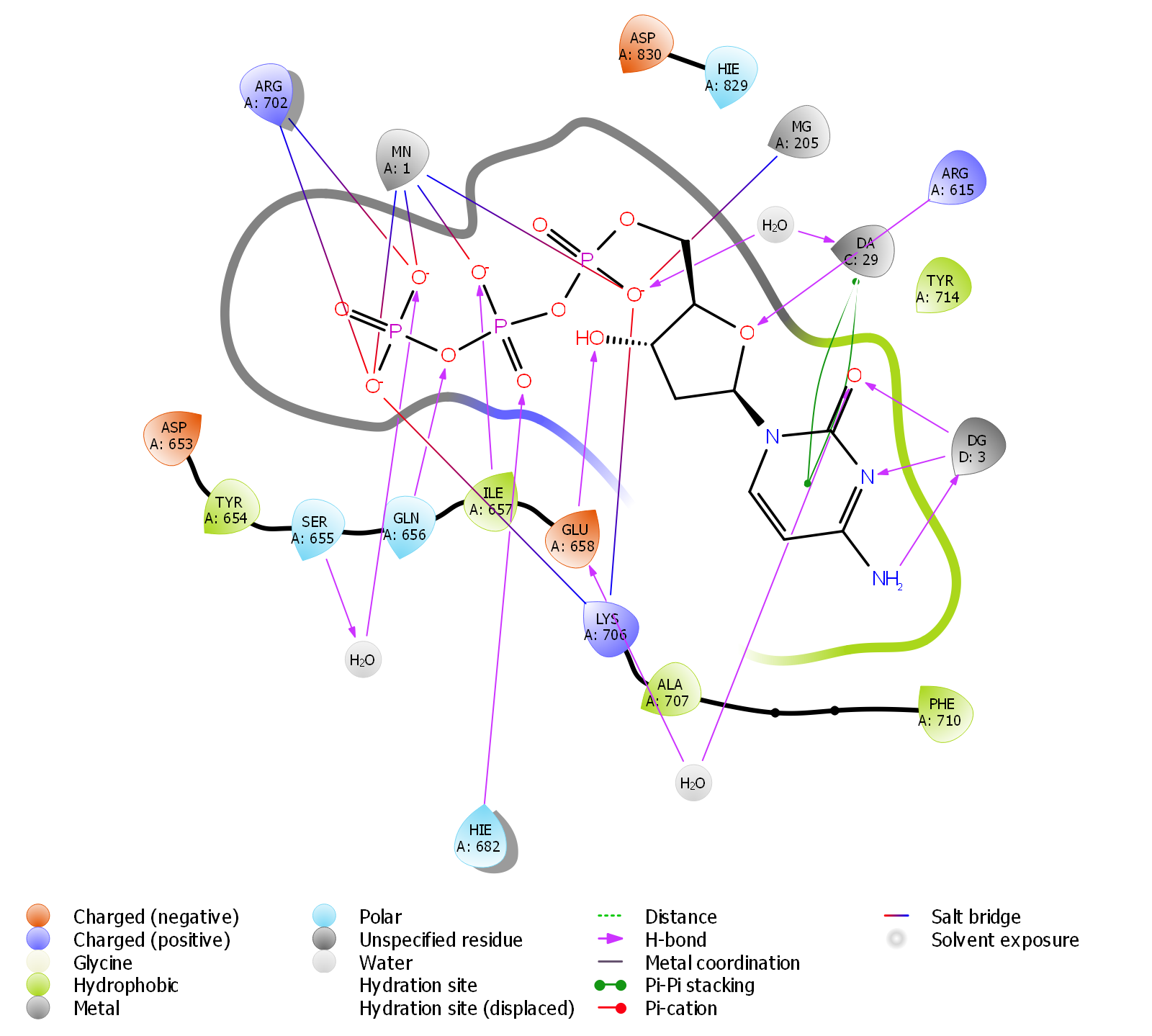

Supplement: Supplementary file 1 [file mmc1.zip › Ca_Ca 2.png]

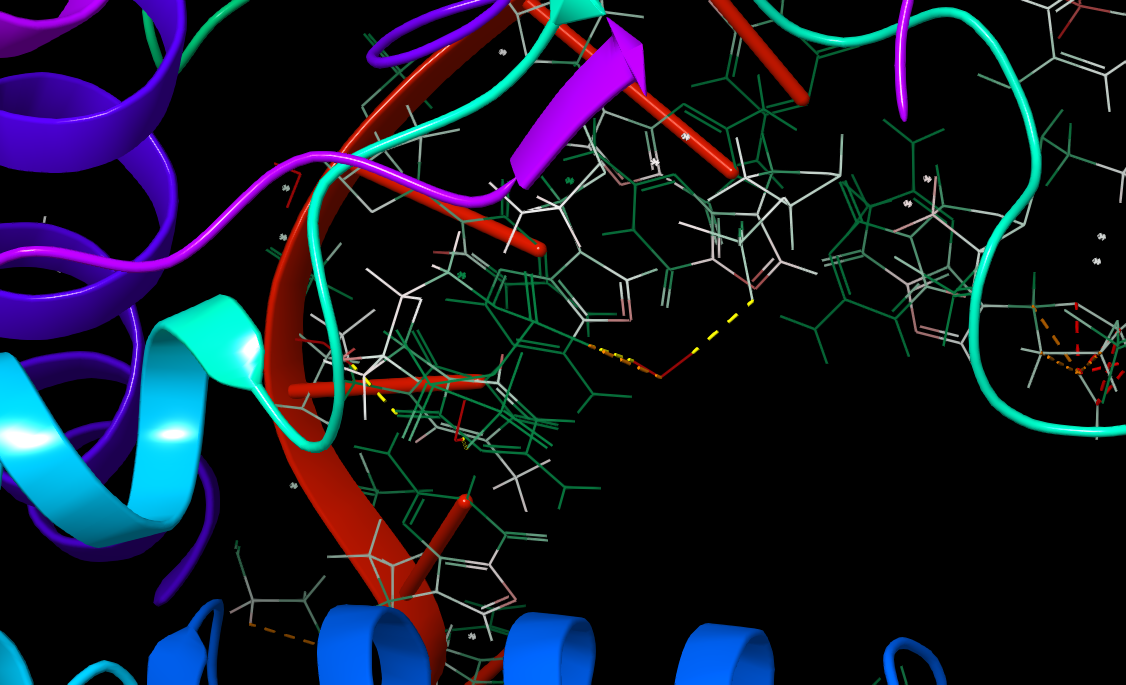

Supplement: Supplementary file 1 [file mmc1.zip › Ca_Ca.png]

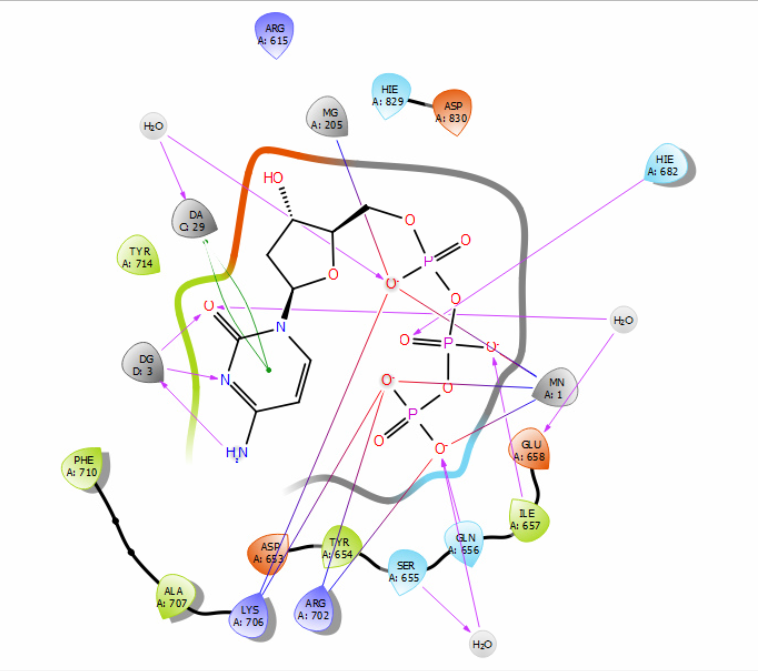

Supplement: Supplementary file 1 [file mmc1.zip › image.png]
